# Principal Component Analyses (PCA)-based findings in population genetic studies are highly biased and must be reevaluated

**DOI:** 10.1038/s41598-022-14395-4

**Published:** 2022-08-29

**Authors:** Eran Elhaik

**Affiliations:** Department of Biology, Lund University, 22362 Lund, Sweden

**Keywords:** Computational models, Population genetics

## Abstract

Principal Component Analysis (PCA) is a multivariate analysis that reduces the complexity of datasets while preserving data covariance. The outcome can be visualized on colorful scatterplots, ideally with only a minimal loss of information. PCA applications, implemented in well-cited packages like EIGENSOFT and PLINK, are extensively used as the foremost analyses in population genetics and related fields (e.g., animal and plant or medical genetics). PCA outcomes are used to shape study design, identify, and characterize individuals and populations, and draw historical and ethnobiological conclusions on origins, evolution, dispersion, and relatedness. The replicability crisis in science has prompted us to evaluate whether PCA results are reliable, robust, and replicable. We analyzed twelve common test cases using an intuitive color-based model alongside human population data. We demonstrate that PCA results can be artifacts of the data and can be easily manipulated to generate desired outcomes. PCA adjustment also yielded unfavorable outcomes in association studies. PCA results may not be reliable, robust, or replicable as the field assumes. Our findings raise concerns about the validity of results reported in the population genetics literature and related fields that place a disproportionate reliance upon PCA outcomes and the insights derived from them. We conclude that PCA may have a biasing role in genetic investigations and that 32,000-216,000 genetic studies should be reevaluated. An alternative mixed-admixture population genetic model is discussed.

## Introduction

The ongoing reproducibility crisis, undermining the foundation of science^1^, raises various concerns ranging from study design to statistical rigor^2,3^. Population genetics is confounded by its utilization of small sample sizes, ignorance of effect sizes, and adoption of questionable study designs. The field is relatively small and may involve financial interests^4–6^ and ethical dilemmas^7,8^. Since biases in the field rapidly propagate to related disciplines like medical genetics, biogeography, association studies, forensics, and paleogenomics in humans and non-humans alike, it is imperative to ask whether and to what extent our most elementary tools satisfy risk criteria.

Principal Component Analysis (PCA) is a multivariate analysis that reduces the data’s dimensionality while preserving their covariance. When applied to genotype bi-allelic data, typically encoded as AA, AB, and BB, PCA finds the eigenvalues and eigenvectors of the covariance matrix of allele frequencies. The data are reduced to a small number of dimensions termed principal components (PCs); each describes a decreased proportion of the genomic variation. Genotypes are then projected onto space spanned by the PC axes, which allows visualizing the samples and their distances from one another in a colorful scatter plot. In this visualization, sample overlap is considered evidence of identity, due to common origin or ancestry^9,10^. PCA’s most attractive property for population geneticists is that the distances between clusters allegedly reflect the genetic and geographic distances between them. PCA also supports the projection of points onto the components calculated by a different dataset, presumably accounting for insufficient data in the projected dataset. Initially adapted for human genomic data in 1963^11^, the popularity of PCA has slowly increased over time. It was not until the release of the SmartPCA tool (EIGENSOFT package)^10^ that PCA was propelled to the front stage of population genetics.

PCA is used as the first analysis of data investigation and data description in most population genetic analyses, e.g., Refs.^12–15^. It has a wide range of applications. It is used to examine the population structure of a cohort or individuals to determine ancestry, analyze the demographic history and admixture, decide on the genetic similarity of samples and exclude outliers, decide how to model the populations in downstream analyses, describe the ancient and modern genetic relationships between the samples, infer kinship, identify ancestral clines in the data, e.g., Refs.^16–19^, detect genomic signatures of natural selection, e.g., Ref.^20^ and identify convergent evolution^21^. PCA or PCA-like tools are considered the ‘gold standard’ in genome-wide studies (GWAS) and GWAS meta-analyses. They are routinely used to cluster individuals with shared genetic ancestry and detect, quantify, and adjust for population structure^22^. PCA is also used to identify cases, controls^23–25^, and outliers (samples or data)^17^, and calculate population structure covariates^26^. The demand for large sample sizes has prompted researchers to “outsource” analyses to direct-to-consumer companies, which employ discretion in their choice of tools, methods, and data—none of which are shared—and return the PCA loadings and other “summary statistics”^27,28^. Loadings are also offered by databases like gnomAD^29^ and the UK Biobank^30^. PCA serves as the primary tool to identify the origins of ancient samples in paleogenomics^14^, to identify biomarkers for forensic reconstruction in evolutionary biology^31^, and geolocalize samples^32^. As of April 2022, 32,000-216,000 genetic papers employed PC scatterplots to interpret genetic data, draw historical and ethnobiological conclusions, and describe the evolution of various taxa from prehistorical times to the present—no doubt Herculean tasks for any scatterplot.

PCA’s widespread use could not have been achieved without several key traits that distinguish it from other tools—all tied to the replicability crisis. PCA can be applied to any numerical dataset, small or large, and it always yields results. It is parameter-free and nearly assumption-free^9^. It does not involve measures of significance, effect size evaluations, or error estimates. It is, by large, a “black box” harboring complex calculations that cannot be traced. Excepting the squared cosines, which is not commonly used, the proportion of explained variance of the data is the single quantity to evaluate the quality of PCA. There is no consensus on the number of PCs to analyze. Price et al.^10^ recommended using 10 PCs, and Patterson et al.^9^ proposed the Tracy–Widom statistic to determine the number of components. However, this statistic is highly sensitive and inflates the number of PCs. In practicality, most authors use the first two PCs, which are expected to reflect genetic similarities that are difficult to observe in higher PCs. The remaining authors use an arbitrary number of PCs or adopt ad hoc strategies to aid their decision, e.g., Ref.^33^. Pardiñas et al.^34^, for example, selected the first five PC “as recommended for most GWAS approaches” and principal components 6, 9, 11, 12, 13, and 19, whereas Wainschtein et al.^35^ preferred the top 280 PCs. There are no proper usage guidelines for PCA, and “innovations” toward less restrictive usage are adopted quickly. Recently, even the practice of displaying the proportion of variation explained by each PC faded as those proportions dwarfed^14^. Since PCA is affected by the choice of markers, samples, populations, the precise implementation, and various flags implemented in the PCA packages—each has an unpredictable effect on the results—replication cannot be expected.

In population genetics, PCA and admixture-like analyses are the de-facto standards used as non-parametric genetic data descriptors. They are considered the hammer and chisel of genetic analyses^36^. Lawson et al.^37^ and Elhaik and Graur^38^ commented on the misuse of admixture-like tools and argued that they should not be used to draw historical conclusions. Thus far, no investigation has thoroughly explored PCA usage and accuracy across most common study designs.

**Figure 1:**
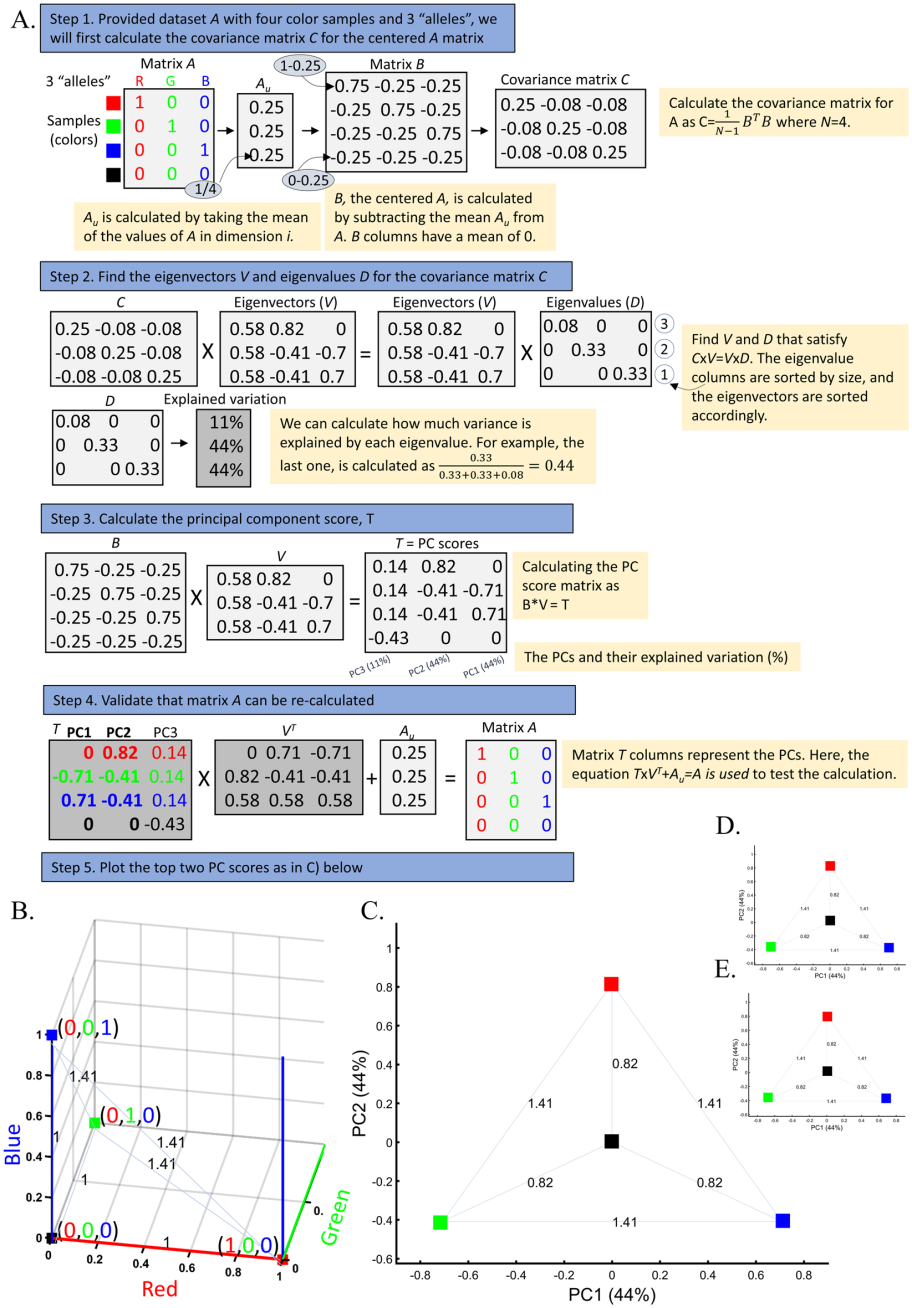
Applying PCA to four color populations. (**A**) An illustration of the PCA procedure (using the singular value decomposition (SVD) approach) applied to a color dataset consisting of four colors (*n*_*All*_ = 1). (**B**) A 3D plot of the original color dataset with the axes representing the primary colors, each color is represented by three numbers (“SNPs”). After PCA is applied to this dataset, the projections of color samples or populations (in their original color) are plotted along their first two eigenvectors (or principal components [PCs]) with (**C**) *n*_*All*_ = 1, (**D**) *n*_*All*_ = 100, and (**E**) *n*_*All*_ = 10,000. The latter two results are identical to those of (**C**). Grey lines and labels mark the Euclidean distances between the color populations calculated across all three PCs.

Because PCA fulfills many of the risk criteria for reproducibility^2^ and its typical usage as a first hypothesis generator in population genetic studies, this study will assess its reliability, robustness, and reproducibility. As PCA is a mathematical model employed to describe the unknown truth, testing its accuracy requires a convincing model where the truth is unambiguous. For that, we developed an intuitive and simple color-based model (Fig. 1A). Because all colors consist of three dimensions—red, green, and blue—they can be plotted in a 3D plot representing the true colors (Fig. 1B). Applied to these data, PCA reduces the dataset to two dimensions that explain most of the variation. This allows us to visualize the true colors (still using their 3D values) in PCA’s 2D scatterplot, measure the distances of the PCs from each other, and compare them to their true 3D distances. We can thereby generate “color populations,” always consisting of 3 variables, analogous to SNPs, to aid us in evaluating the accuracy of PCA. If PCA works well, we expect it to properly represent the true distances of the colors from one another in a 2D plot (i.e., light Green should cluster near Green; Red, Green, and Blue should cluster away from each other). Let us agree that if PCA cannot perform well in this simplistic setting, where subpopulations are genetically distinct (*F*_*ST*_ is maximized), and the dimensions are well separated and defined, it should not be used in more complex analyses and certainly cannot be used to derive far-reaching conclusions about history. In parallel, we analyzed genotype data of modern and ancient human populations. Because the inferred population structure and population history may be debatable, we asked whether and to what extent PCA can generate contradictory results and lead to absurd conclusions (*reductio ad absurdum*), whether seemingly “correct” conclusions can be derived without prior knowledge (*cherry-picking* or *circular reasoning*), and whether PCA grants a posteriori knowledge independent of experience (a priori). Let us also agree that if the answer to any of those questions is negative, PCA is of no use to population geneticists.

We carried out an extensive empirical evaluation of PCA through twelve test cases, each assessing a typical usage of PCA using color and human genomic data. In all the cases, we applied PCA according to the standards in the literature but modulated the choice of populations, sample sizes, and, in one case, the selection of markers. The PCA tool used here yields near-identical results to the PCA implemented in EIGENSOFT (Supplementary Figs. S1–S2). To illustrate the way PCA can be used to support multiple opposing arguments in the same debate, we constructed fictitious scenarios with parallels to many investigations in human ancestry that are shown in boxes. We reasoned that if PCA results are irreproducible, contradictory, or absurd, and if they can be manipulated, directed, or controlled by the experimenter, then PCA must not be used for genetic investigations, and an incalculable number of findings based on its results should be reevaluated. We found that this is indeed the case.

## Results

### The near-perfect case of dimensionality reduction

Applying principal component analysis (PCA) to a dataset of four populations sampled evenly: the three primary colors (Red, Green, and Blue) and Black illustrate a near-ideal dimension reduction example. PCA condensed the dataset of these four samples from a 3D Euclidean space (Fig. 1B) into three principal components (PCs), the first two of which explained 88% of the variation and can be visualized in a 2D scatterplot (Fig. 1C). Here, and in all other color-based analyses, the colors represent the true 3D structure, whereas their positions on the 2D plots are the outcome of PCA. Although PCA correctly positioned the primary colors at even distances from each other and Black, it distorted the distances between the primary colors and Black (from 1 in 3D space to 0.82 in 2D space). Thereby, even in this limited and near-perfect demonstration of data reduction, the observed distances do not reflect the actual distances between the samples (which are impossible to recreate in a 2D dataset). In other words, distances between samples in a reduced dimensionality plot do not and cannot be expected to represent actual genetic distances. Evenly increasing all the sample sizes yields identical results irrespective of the sample size (Fig. 1D,E).

**Figure 2:**
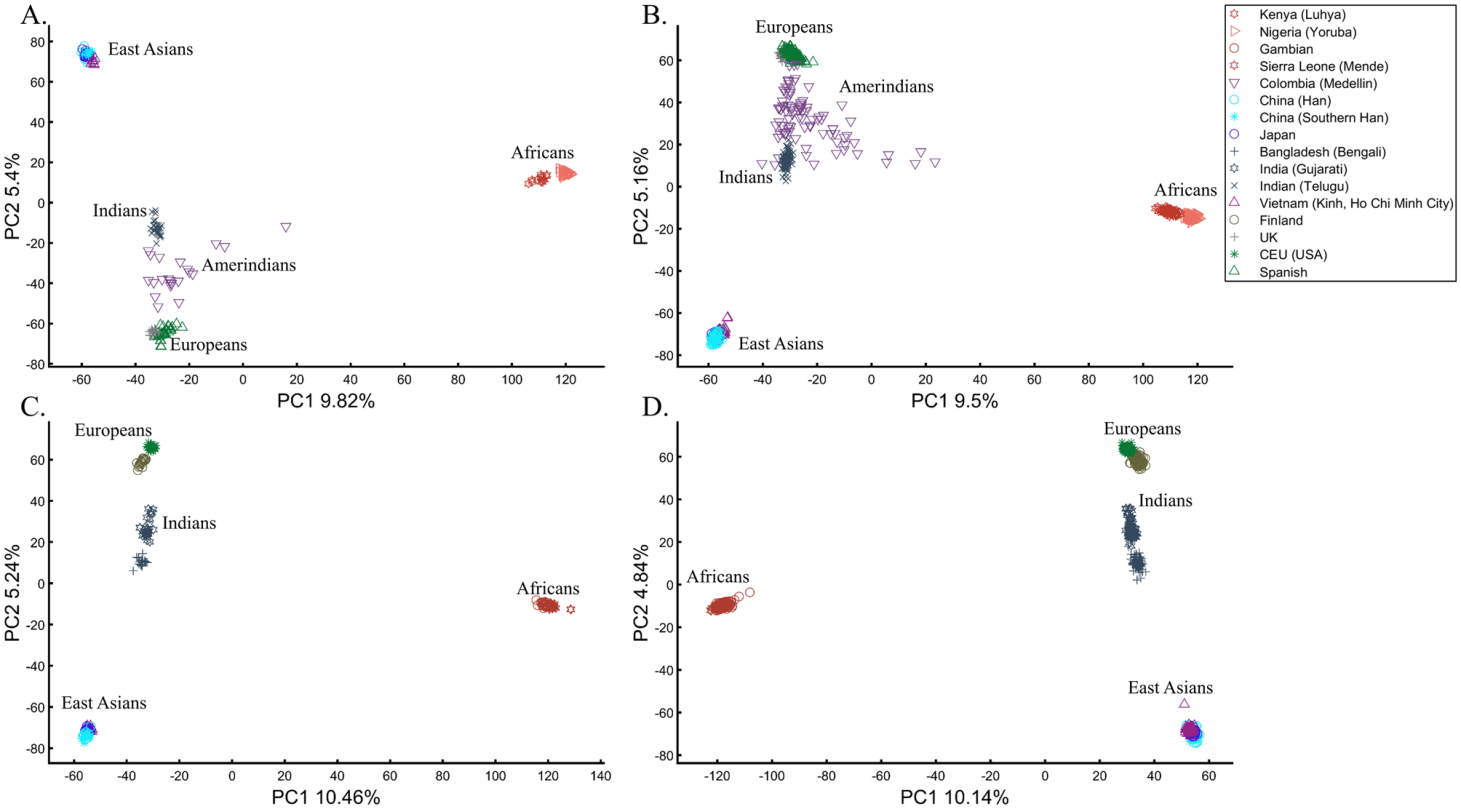
Testing the effect of even-sample sizes using two population sets. The top plots show nine populations with *n* = 50 (**A**) and *n* = 188 (**B**). The bottom plots show a different set of nine populations with *n* = 50 (**C**) and *n* = 192 (**D**). In both cases, increasing the sample size did not alter the PCs (the y-axis flip between (**C**) and (**D**) is a known phenomenon).

When analyzing human populations, which harbor most of the genomic variation between continental populations (12%) with only 1% of the genetic variation distributed within continental populations^39^, PCA tends to position Africans, Europeans, and East Asians at the corners of an imaginary triangle, which closely resembles our color-population model and illustration. Analyzing continental populations, we obtained similar results for two even-sized sample datasets (Fig. 2A,C) and their quadrupled counterparts (Fig. 2B,D). As before, the distances between the populations remain similar (Fig. 2A–D), demonstrating that for same-sized populations, sample size does not contribute to the distortion of the results if the increase in size is proportional.

### The case of different sample sizes

The extent to which different-sized populations produce results with conflicting interpretations is illustrated through a typical study case in Box 1.

**Figure 3:**
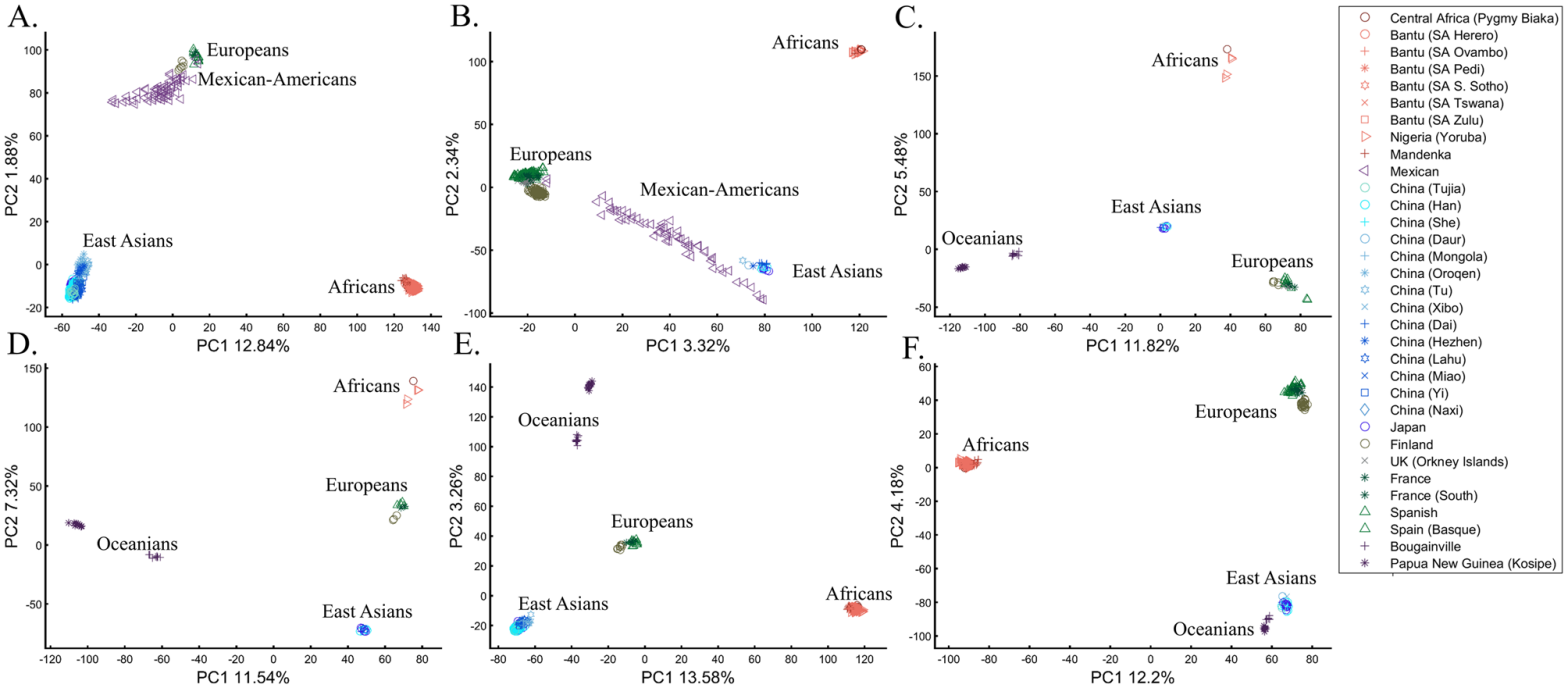
PCA of uneven-sized African (Af), European (Eu), Asian (As), and Mexican-Americans (Ma) or Oceanian (Oc) populations. Fixing the sample size of Mexican-Americans and altering the sample sizes of other populations: (**A**) *n*_*Af*_ = 198; *n*_*Eu*_ = 20; *n*_*As*_ = 483; *n*_*Ma*_ = 64 and (**B**) *n*_*Af*_ = 20; *n*_*Eu*_ = 343; *n*_*Ma*_ = 20; *n*_*Am*_ = 64 changes the results. An even more dramatic change can be seen when repeating this analysis on Oceanians: (**C**) *n*_*Af*_ = 5; *n*_*Eu*_ = 25; *n*_*As*_ = 10; *n*_*Oce*_ = 20 and (**D**) *n*_*Afr*_ = 5; *n*_*Eu*_ = 10; *n*_*As*_ = 15; *n*_*Oc*_ = 20 and when altering their sample sizes: (**E**) *n*_*Af*_ = 98; *n*_*Eu*_ = 25; *n*_*As*_ = 150; *n*_*Oc*_ = 24 and (**F**) *n*_*Af*_ = 98; *n*_*Eu*_ = 83; *n*_*As*_ = 30; *n*_*Oc*_ = 15.

**Figure 4:**
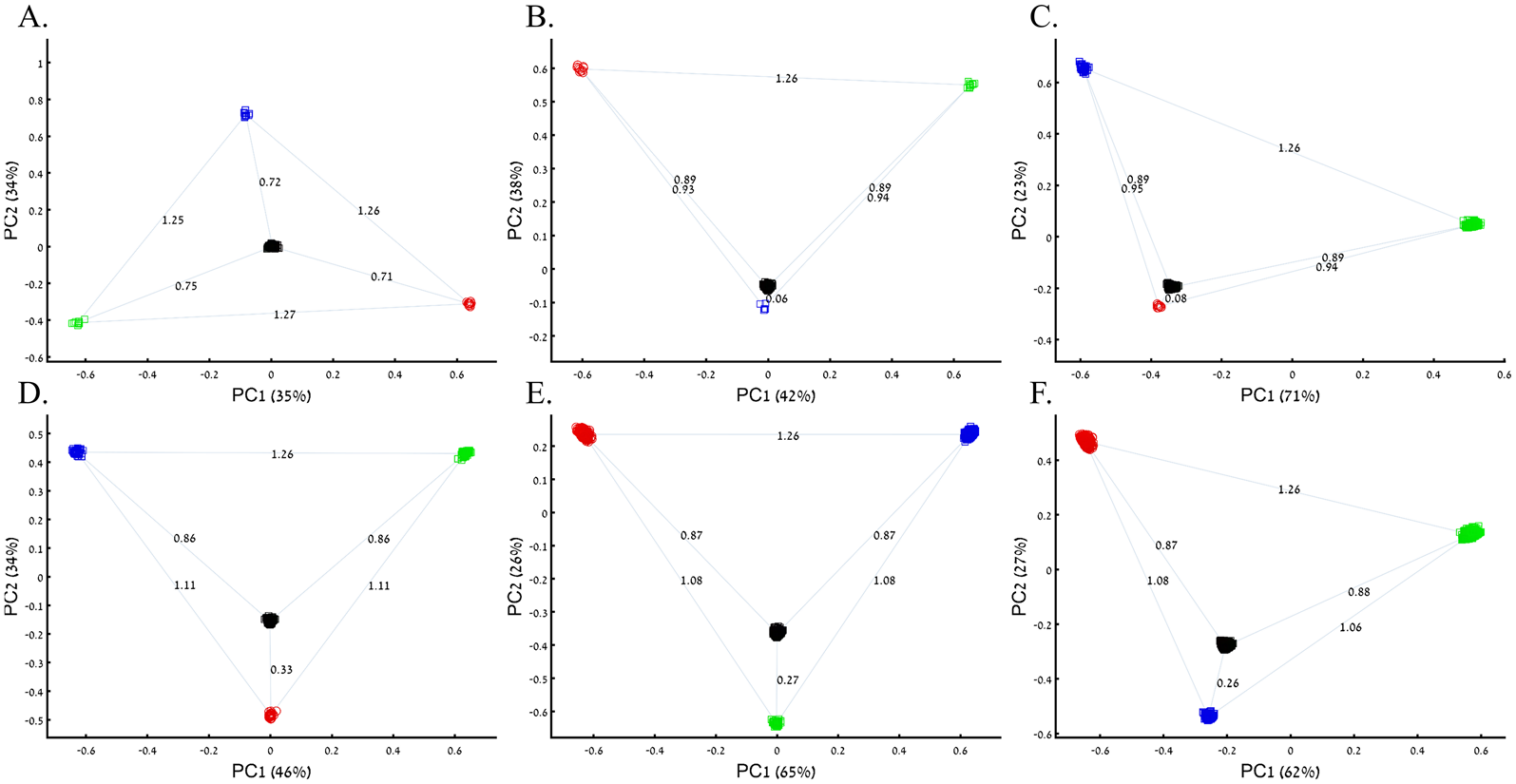
PCA of uneven-sized samples of four color populations. (**A**) *n*_*Red*_ = *n*_*Green*_ = *n*_*Blue*_ = 10; *n*_*Black*_ = 200, (**B**) *n*_*Red*_ = *n*_*Green*_ = 10; *n*_*Blue*_ = 5; *n*_*Black*_ = 200, (**C**) *n*_*Red*_ = 10; *n*_*Green*_ = 200; *n*_*Blue*_ = 50; *n*_*Black*_ = 200 (**D**) *n*_*Red*_ = 25; *n*_*Green*_ = *n*_*Blue*_ = 50; *n*_*Black*_ = 200, (**E**) *n*_*Red*_ = 300; *n*_*Green*_ = 200; *n*_*Blue*_ = *n*_*Black*_ = 300, and (**F**) *n*_*Red*_ = 1000; *n*_*Green*_ = 2000; *n*_*Blue*_ = 300; *n*_*Black*_ = 2000. Scatter plots show the top two PCs. The numbers on the grey bars reflect the Euclidean distances between the color populations over all PCs. Colors include Red [1,0,0], Green [0,1,0], Blue [0,0,1], and Black [0,0,0].

Note that unlike in Figs. 1C and 3A, where Black is in the middle, in other figures, the overrepresentation of certain “alleles” (e.g., Fig. 4B) shifts Black away from (0,0). Intuitively, this can be thought of as the most common “allele” (Green in Fig. 4B) repelling Black, which has three null or alternative “alleles”.

PCA is commonly reported as yielding a stable differentiation of continental populations (e.g., Africans vs. non-Africans, Europeans vs. Asians, and Asians vs. Native Americans or Oceanians, on the primary PCs^40–43^). This prompted prehistorical inferences of migrations and admixture, viewing the PCA results that position Africans, East Asians, and Europeans in three corners of an imaginary triangle as representing the post Out Of Africa event followed by multiple migrations, differentiation, and admixture events. Inferences for Amerindians or Aboriginals typically follow this reconstruction. For instance, Silva-Zolezzi et al.^42^ argued that the Zapotecos did not experience a recent admixture due to their location on the Amerindian PCA cluster at the Asian end of the European-Asian cline.

Here we show that the appearance of continental populations at the corners of a triangle is an artifact of the sampling scheme since variable sample sizes can easily create alternative results as well as alternative “clines”. We first replicated the triangular depiction of continental populations (Fig. 3A,B) before altering it (Fig. 3C–F). Now, East Asians appear as a three-way admixed group of Africans, Europeans, and Melanesians (Fig. 3C), whereas Europeans appear on an African-East Asian cline (Fig. 3D). Europeans can also be made to appear in the middle of the plot as an admixed group of Africans-Asians-Oceanians origins (Fig. 3E), and Oceanians can cluster with (Fig. 3F) or without East Asians (Fig. 3E). The latter depiction maximizes the proportion of explained variance, which common wisdom would consider the correct explanation. According to some of these results, only Europeans and Oceanians (Fig. 3C) or East Asians and Oceanians (Fig. 3D) experienced the Out of Africa event. By contrast, East Asians (Fig. 3C) and Europeans (Fig. 3D) may have remained in Africa. Contrary to Silva-Zolezzi et al.’s^42^ claim, the same Mexican–American cohort can appear closer to Europeans (Fig. 3A) or as a European-Asian admixed group (Fig. 3B). It is easy to see that none of those scenarios stand out as more or less correct than the other ones.

Reich et al.^44^ presented further PCA-based “evidence” to the ‘out of Africa’ scenario. Applying PCA to Africans and non-Africans, they reported that non-Africans cluster together at the center of African populations when PC1 was plotted against PC4 and that this “rough cluster[ing]” of non-Africans is “about what would be expected if all non-African populations were founded by a single dispersal ‘out of Africa.’” However, observing PC1 and PC4 for Supplementary Fig. S3, we found no “rough cluster” of non-Africans at the center of Africans, contrary to Reich et al.’s^44^ claim. Remarkably, we found a “rough cluster” of Africans at the center of non-Africans (Supplementary Fig. S3C), suggesting that Africans were founded by a single dispersal ‘into Africa’ by non-Africans. We could also infer, based on PCA, either that Europeans never left Africa (Supplementary Fig. S3D), that Europeans left Africa through Oceania (Supplementary Fig. S3B), that Asians and Oceanians never left Europe (or the other way around) (Supplementary Fig. S3F), or, since all are valid PCA results, all of the above. Unlike Reich et al.^44^, we do not believe that their example “highlights how PCA methods can provide evidence of important migration events”. Instead, our examples (Fig. 3, Supplementary Fig. S3) show how PCA can be used to generate conflicting and absurd scenarios, all mathematically correct but, obviously, biologically incorrect and cherry-pick the most favorable solution. This is an example of how vital a priori knowledge is to PCA. It is thereby misleading to present one or a handful of PC plots without acknowledging the existence of many other solutions, let alone while not disclosing the proportion of explained variance.

Box 1: Studying the origin of Black using the primary colors Three research groups sought to study the origin of Black. A previous study that employed even sample-sized color populations alluded that Black is a mixture of all colors (Fig. 1B–D). A follow-up study with a larger sample size (*n*_*Red*_ = *n*_*Green*_ = *n*_*Blue*_ = 10) and enriched in Black samples (*n*_*Black*_ = 200) (Fig. 4A) reached the same conclusion. However, the Black-is-Blue group suspected that the Blue population was mixed. After QC procedures, the Blue sample size was reduced, which decreased the distance between Black and Blue and supported their speculation that Black has a Blue origin (Fig. 4B). The Black-is-Red group hypothesized that the underrepresentation of Green, compared to its actual population size, masks the Red origin of Black. They comprehensively sampled the Green population and showed that Black is very close to Red (Fig. 4C). Another Black-is-Red group contributed to the debate by genotyping more Red samples. To reduce the bias from other color populations, they kept the Blue and Green sample sizes even. Their results replicated the previous finding that Black is closer to Red and thereby shares a common origin with it (Fig. 4D). A new Black-is-Green group challenged those results, arguing that the small sample size and omission of Green samples biased the results. They increased the sample sizes of the populations of the previous study and demonstrated that Black is closer to Green (Fig. 4E). The Black-is-Blue group challenged these findings on the grounds of the relatively small sample sizes that may have skewed the results and dramatically increased all the sample sizes. However, believing that they are of Purple descent, Blue refused to participate in further studies. Their relatively small cohort was explained by their isolation and small effective population size. The results of the new sampling scheme confirmed that Black is closer to Blue (Fig. 4F), and the group was praised for the large sample sizes that, no doubt, captured the actual variation in nature better than the former studies.

### The case of one admixed population

The question of who the ancestors of admixed populations are and the extent of their contribution to other groups is at the heart of population genetics. It may not be surprising that authors hold conflicting views on interpreting these admixtures from PCA. Here, we explore how an admixed group appears in PCA, whether its ancestral groups are identifiable, and how its presence affects the findings for unmixed groups through a typical study case (Box 2).

To understand the impact of parameter choices on the interpretation of PCA, we revisited the first large-scale study of Indian population history carried out by Reich et al.^45^. The authors applied PCA to a cohort of Indians, Europeans, Asians, and Africans using various sample sizes that ranged from 2 (Srivastava) (out of 132 Indians) to 203 (Yoruban) samples. After applying PCA to Indians and the three continental populations to exclude “outliers” that supposedly had more African or Asian ancestries than other samples, PCA was applied again in various settings.

At this point, the authors engaged in circular logic as, on the one hand, they removed samples that appeared via PCA to have experienced gene flow from Africa (their Note 2, *iii*) and, on the other hand, employed a priori claim (unsupported by historical documents) that “African history has little to do with Indian history” (which must stand in sharp contrast to the rich history of gene flow from Utah (US) residents to Indians, which was equally unsupported). Reich et al. provided no justification for the exact protocol used or any discussion about the impact of using different parameter values on resulting clusters. They then generated a plethora of conflicting PCA figures, never disclosing the proportion of explained variance along with the first four PCs examined. They then inferred based on PCA that Gujarati Americans exhibit no “unusual relatedness to West Africans (YRI) or East Asians (CHB or JPT)” (Supplementary Fig. S4)^45^. Their concluding analysis of Indians, Asians, and Europeans (Fig. 4)^45^ showed Indians at the apex of a triangle with Europeans and Asians at the opposite corners. This plot was interpreted as evidence of an “ancestry that is unique to India” and an “Indian cline”. Indian groups were explained to have inherited different proportions of ancestry from “Ancestral North Indians” (ANI), related to western Eurasians, and “Ancestral South Indians” (ASI), who split from Onge. The authors then followed up with additional analyses using Africans as an outgroup, supposedly confirming the results of their selected PCA plot. Indians have since been described using the terms ANI and ASI.

**Figure 5:**
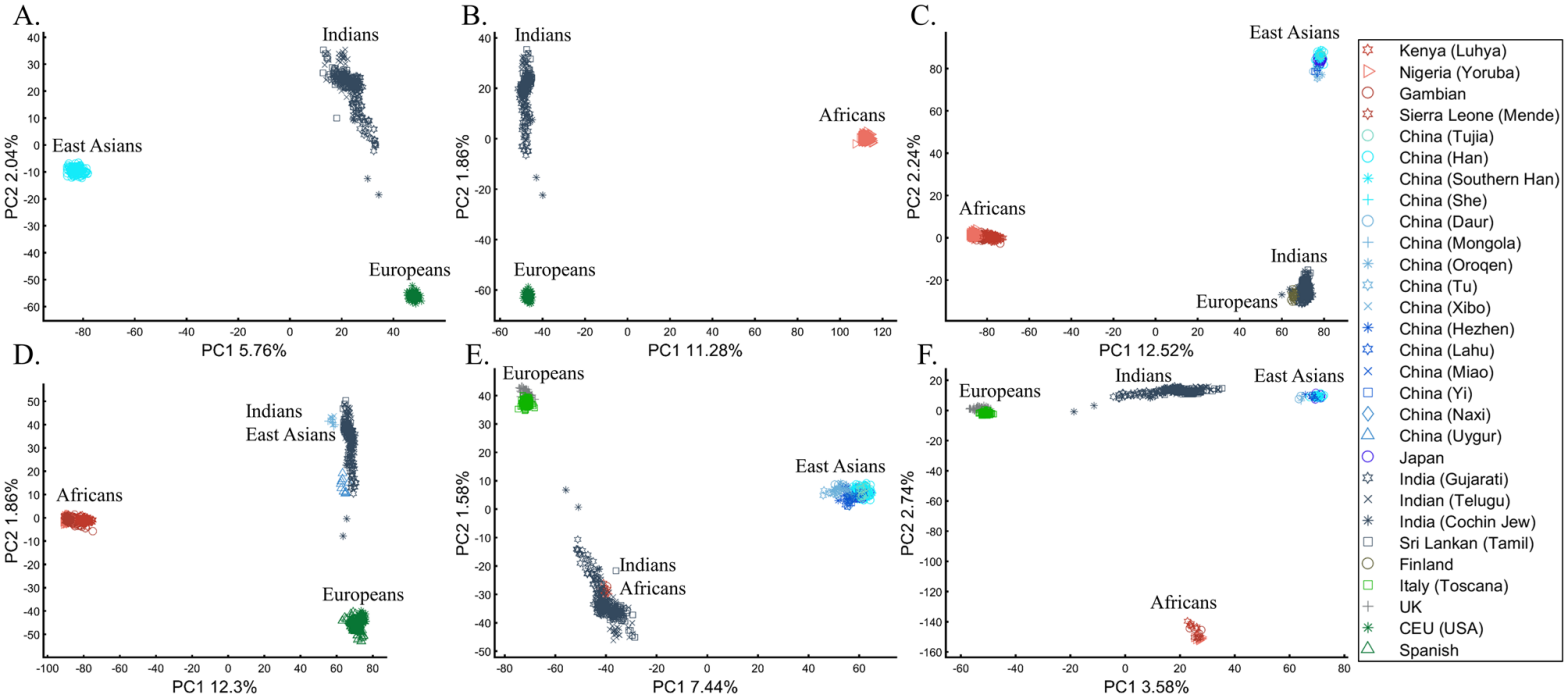
Studying the origin of Indians using PCA. (**A**) Replicating Reich et al.’s ^45^ results using *n*_*Eu*_ = 99; *n*_*As*_ = 146; *n*_*Ind*_ = 321. Generating alternative PCA scenarios using: (**B**) *n*_*Af*_ = 178; *n*_*Eu*_ = 99; *n*_*Ind*_ = 321, (**C**) *n*_*Af*_ = 400; *n*_*Eu*_ = 40; *n*_*As*_ = 100; *n*_*Ind*_ = 321, (**D**) *n*_*Af*_ = 477; *n*_*Eu*_ = 253; *n*_*As*_ = 23; *n*_*Ind*_ = 321, (**E**) *n*_*Af*_ = 25; *n*_*Eu*_ = 220; *n*_*As*_ = 490; *n*_*Ind*_ = 320, and (**F**) *n*_*Af*_ = 30; *n*_*Eu*_ = 200; *n*_*As*_ = 50; *n*_*Ind*_ = 320.

In evaluating the claims of Reich et al.^45^ that rest on PCA, we first replicated the finding of the alleged “Indian cline” (Fig. 5A). We next garnered support for an alternative cline using Indians, Africans, and Europeans (Fig. 5B). We then demonstrated that PCA results support Indians to be European (Fig. 5C), East Asians (Fig. 5D), and Africans (Fig. 5E), as well as a genuinely European-Asian, admixed population (Fig. 5F). Whereas the first two PCs of Reich et al.’s primary figure explain less than 8% of the variation (according to our Fig. 5A, Reich et al.’s Fig. 4 does not report this information), four out of five of our alternative depictions explain 8–14% of the variation. Our results also expose the arbitrariness of the scheme used by Reich et al. and show how radically different clustering can be obtained merely by manipulating the non-Indian populations used in the analyses. Our results also question the authors’ choice in using an analysis that explained such a small proportion of the variation (let alone not reporting it), yielded no support for a unique ancestry to India, and cast doubt on the reliability and usefulness of the ANI-ASI model to describe Indians provided their exclusive reliability on a priori knowledge in interpreting the PCA patters. Although supported by downstream analyses, the plurality of PCA results could not be used to support the authors’ findings because using PCA, it is impossible to answer a priori whether Africa is in India or the other way around (Fig. 5E). We speculate tat the motivation for Reich et al.'s strategy was to declare Africans an outgroup, an essential component of D-statistics. Clearly, PCA-based a posteriori inferences can lead to errors of Colombian magnitude.

**Figure 6:**
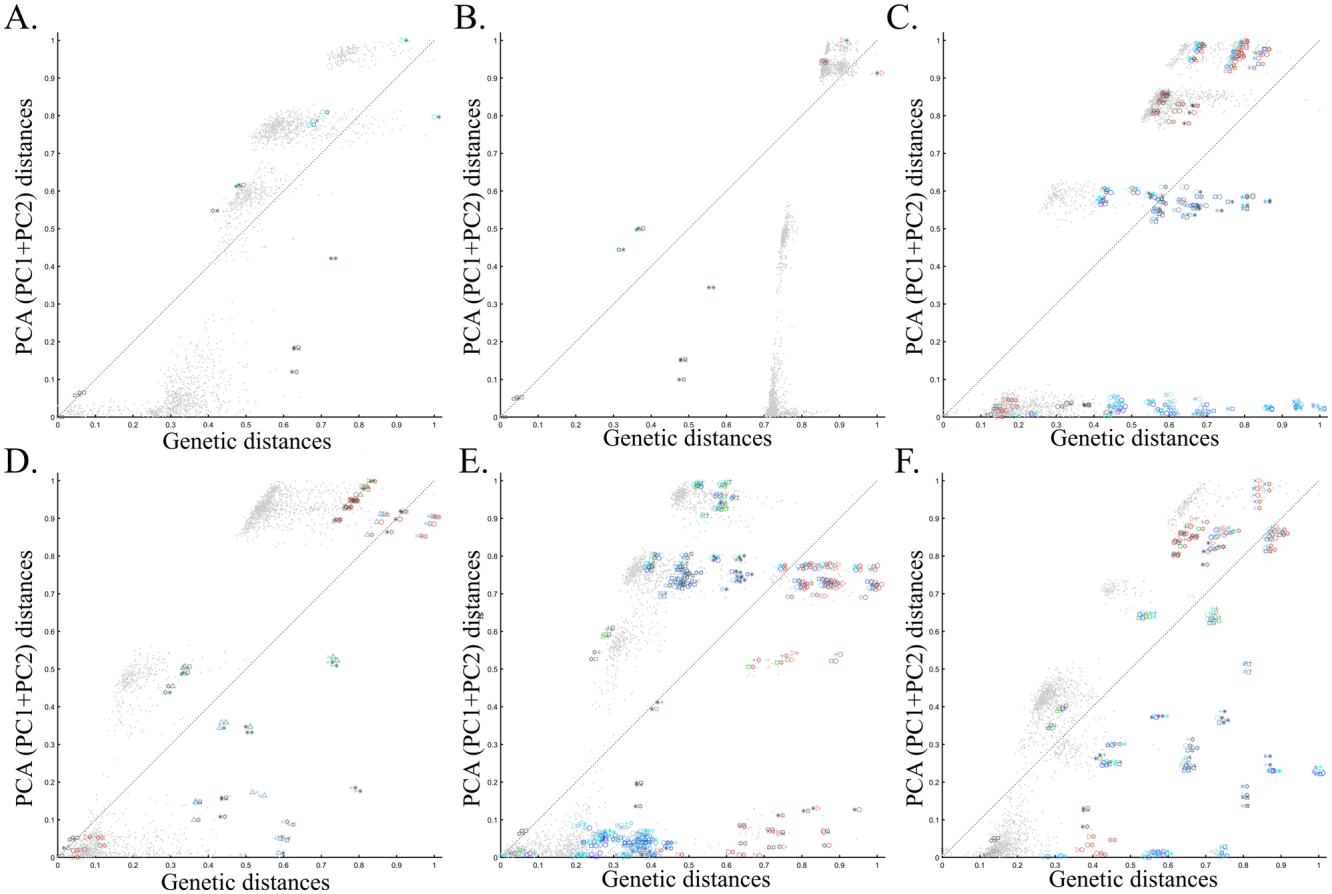
Comparing the genetic distances with PCA-based distances for the corresponding datasets of Fig. 5. Genetic and PCA (PC1 + PC2) distances between populations pairs (symbol pairs) and 2000 random individual pairs (grey dots) were calculated using Euclidean distances and normalized to range from 0 to 1. Population and individual pairs whose PC distances reflect their genetic distances are shown along the x = y dotted line. Note that the position of heterogeneous populations on the plot may deviate from that of their samples and that some populations are very small.

To evaluate the extent of deviation of PCA results from genetic distances, we adopted a simple genetic distance scheme where we measured the Euclidean distance between allelic counts (0,1,2) in the same data used for PCA calculations. We are aware of the diversity of existing genetic distance measures. However, to the best of our knowledge, no study has ever shown that PCA outcomes numerically correlate with any genetic distance measure, except in very simple scenarios and tools like ADMIXTURE-like tools, which, like PCA, exhibit high design flexibility. Plotting the genetic distances against those obtained from the top two PCs shows the deviation between these two measures for each dataset. We found that all the PC projections (Fig. 6) distorted the genetic distances in unexpected ways that differ between the datasets. PCA correctly represented the genetic distances for a minority of the populations, and just like the most poorly represented populations—none were distinguishable from other populations. Moreover, populations that clustered under PCA exhibited mixed results, questioning the accuracy of PCA clusters. Although it remains unclear which sampling scheme to adopt, neither scheme is genetically accurate. These results further question the genetic validity of the ANI-ASI model.

We are aware that PCA disciples may reject our *reductio ad absurdum* argument and attempt to read into these results, as ridiculous as they may be, a valid description of Indian ancestry. For those readers, demonstrating the ability of the experimenter to generate near-endless contradictory historical scenarios using PCA may be more convincing or at least exhausting. For brevity, we present six more such scenarios that show PCA support for Indians as a heterogeneous group with European admixture and Mexican-Americans as an Indian-European mixed population (Supplementary Fig. S4A), Mexican–American as an admixed African-European group with Indians as a heterogeneous group with European admixture (Supplementary Fig. S4B), Indians and Mexican-Americans as European-Japanese admixed groups with common origins and high genetic relatedness (Supplementary Fig. S4C), Indians and Mexican-Americans as European-Japanese admixed groups with no common origins and genetic relatedness (Supplementary Fig. S4D), Europans as Indian and Mexican-Americans admixed group with Japanese fully cluster with the latter (Supplementary Fig. S4E), and Japanese and Europeans cluster as an admixed Indian and Mexican-Americans groups (Supplementary Fig. S4F). Readers are encouraged to use our code to produce novel alternative histories. We suspect that almost any topology could be obtained by finding the right set of input parameters. In this sense, any PCA output can reasonably be considered meaningless.

Contrary to Reich et al.'s claims, a more common interpretation of PCA is that the populations at the corners of the triangle are ancestral or are related to the mixed groups within the triangle, which are the outcome of admixture events, typically referred to as “gradient” or “clines^45^”. However, some authors held different opinions. Studying the African component of Ethiopian genomes, Pagani et al.^46^ produced a PC plot showing Europeans (CEU), Yoruba (western African), and Ethiopians (Eastern Africans) at the corners of a triangle (Supplementary Fig. S4)^46^. Rather than suggesting that the populations within the triangle (e.g., Egyptians, Spaniards, Saudi) are mixtures of these supposedly ancestral populations, the authors argued that Ethiopians have western and eastern African origins, unlike the central populations with “different patterns of admixture”. Obviously, neither interpretation is correct. Reich et al.’s interpretation does not explain why CEUs are not an Indian-African admix nor why Africans are not a European-Indian admix and is analogous to arguing that Red has Green and Blue origins (Fig. 1). Pagani et al.’s interpretation is a tautology, ignores the contribution of non-Africans, and is analogous to arguing that Red has Red and Green origins. We carried out forward simulations of populations with various numbers of ancestral populations and found that admixture cannot be inferred from the positions of samples in a PCA plot (Supplementary Text 1).

In a separate effort to study the origins of AJs, Need et al.^47^ applied PCA to 55 Ashkenazic Jews (AJs) and 507 non-Jewish Caucasians. Their PCA plot showed that AJs (marked as “Jews”) formed a distinct cluster from Europeans (marked as “non-Jews”). Based on these results, the authors suggested that PCA can be used to detect linkage to Jewishness. A follow-up PCA where Middle Eastern (Bedouin, Palestinians, and Druze) and Caucasus (Adygei) populations were included showed that AJs formed a distinct cluster that nested between the Adygei (and the European cluster) and Druze (and the Middle Eastern cluster). The authors then concluded that AJs might have mixed Middle Eastern and European ancestries. The proximity to the Adygei cluster was noted as interesting but dismissed based on the small sample size of the Adygei (*n* = 17). The authors concluded that AJ genomes carry an “unambiguous signature of their Jewish heritage, and this seems more likely to be due to their specific Middle Eastern ancestry than to inbreeding”. A similar strategy was employed by Bray et al.^48^ to claim that PCA “confirmed that the AJ individuals cluster distinctly from Europeans, aligning closest to Southern European populations along with the first principal component, suggesting a more southern origin, and aligning with Central Europeans along the second, consistent with migration to this region.” Other authors^49,50^ made similar claims.

**Figure 7:**
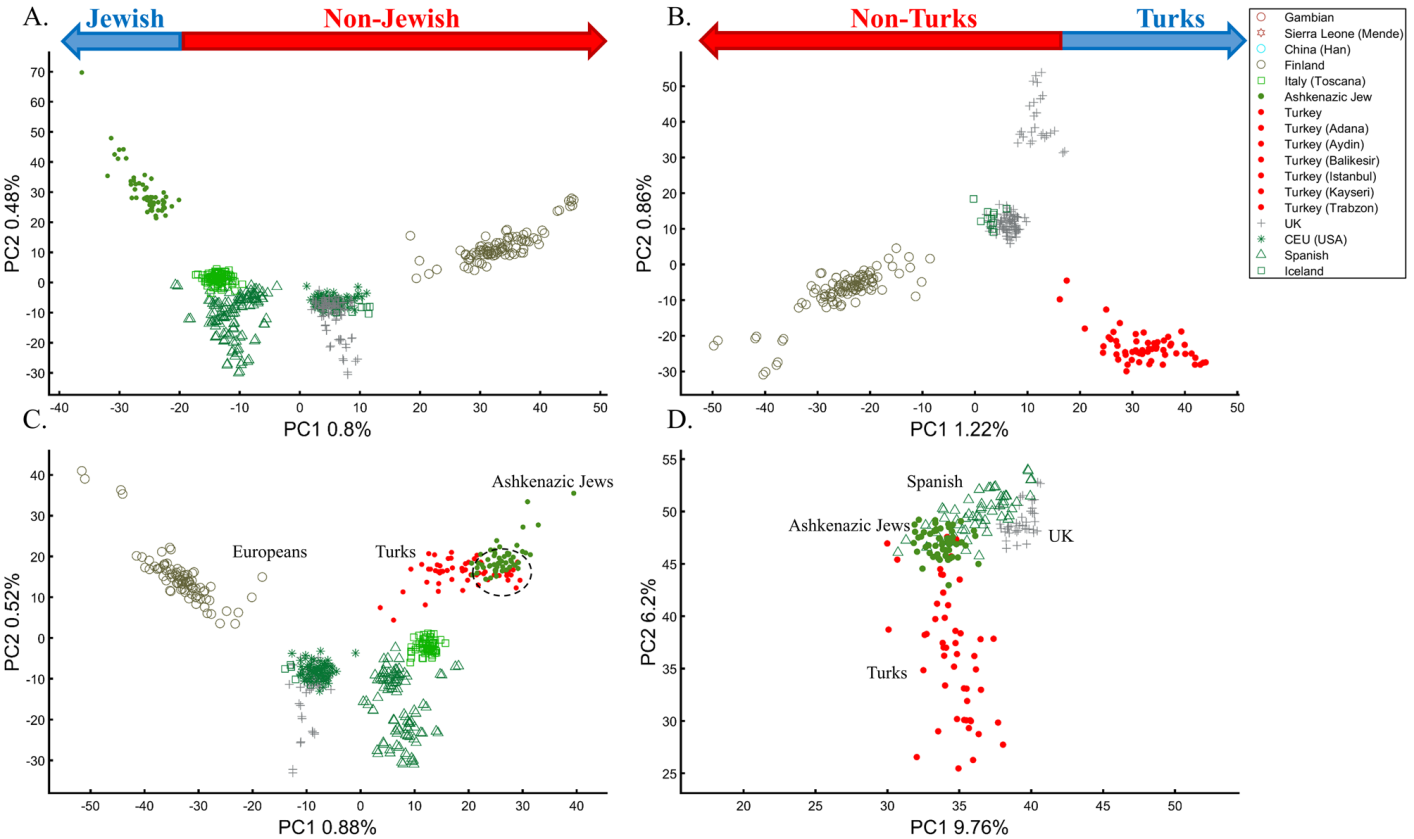
Studying the origin of 55 AJs using PCA. (**A**) Replicating Need et al.’s results using *n*_*Eu*_ = 507; Generating alternative PCA scenarios using: (**B**) *n*_*Eu*_ = 223; *n*_*Turks*_ = 56; (**C**) *n*_*Eu*_ = 400; *n*_*Turks*+*Caucasus*_ = 56, and (**D**) *n*_*Af*_ = 100, *n*_*As*_ = 100 (Africans and Asians are not shown), *n*_*Eu*_ = 100; and *n*_*Turks*_ = 50. Need et al.'s faulty terminology was adopted in **A** and **B**.

It is easy to show why PCA cannot be used to reach such conclusions. We first replicated Need et al.’s^47^ primary results (Fig. 7A), showing that AJs cluster separately from Europeans. However, such an outcome is typical when comparing Europeans and non-European populations like Turks (Fig. 7B). It is not unique to AJs, nor does it prove that they are genetically detectable. A slightly modified design shows that most AJs overlap with Turks in support of the Turkic (or Near Eastern) origin of AJs (Fig. 7C). We can easily refute our conclusion by including continental populations and showing that most AJs cluster with Iberians rather than Turks (Fig. 7D). This last design explains more of the variance than all the previous analyses together, although, as should be evident by now, it is not indicative of accuracy. This analysis questions PCA's use as a discriminatory genetic utility and to infer genetic ancestry.

There are several more oddities with the report of Need et al.^47^. First, they did not report the variance explained by their sampling scheme (it is, likely, ~1%, as in Fig. 7A). Second, they misrepresented the actual populations analyzed. AJs are not the only Jews, and Europeans are not the only non-Jews (Figs. 1, 7A)^47^. Finally, their dual interpretations of AJs as a mixed population of Middle Eastern origin are based solely on a priori belief: first, because most of the populations in their PCA are nested between and within other populations, yet the authors did not suggest that they are all admixed and second because AJs nested between Adygii and Druze^51,52^, both formed in the Near Eastern. The conclusions of Need et al.^47^ were thereby obtained based on particular PCA schemes and what may be preconceived ideas of AJs origins that are no more real than the Iberian origin of AJs (Fig. 7D). This is yet another demonstration (discussed in Elhaik^36^) of how PCA can be misused to promote ethnocentric claims due to its design flexibility.

**Figure 8:**
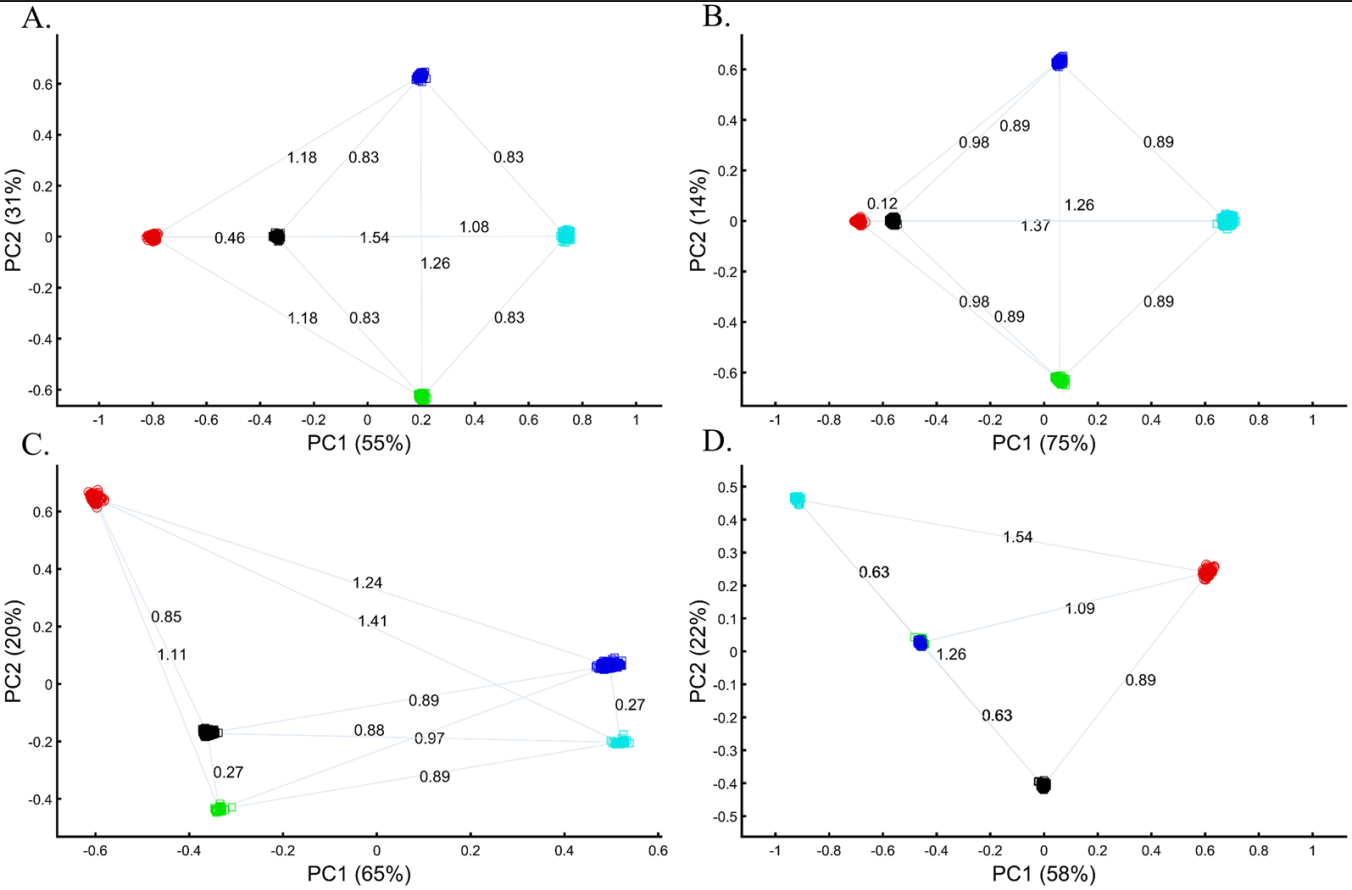
PCA with the primary and mixed color populations. (**A**) *n*_*all*_ = 100; *n*_*Black*_ = 200, (**B**) *n*_*Red*_ = *n*_*Green*_ = *n*_*Blue*_ = 100; *n*_*Black*_ = *n*_*Cyan*_ = 500, (**C**) *n*_*Red*_ = 100; *n*_*Green*_ = *n*_*Cyan*_ = 33; *n*_*Blue*_ = *n*_*Black*_ = 400; and (**D**) *n*_*Red*_ = *n*_*Black*_ = 100; *n*_*Green*_ = *n*_*Blue*_ = *n*_*Cyan*_ = 33; Scatter plots show the top two PCs. The numbers on the grey bars reflect the Euclidean distances between the color populations over all PCs. Colors include Red [1,0,0], Green [0,1,0], Blue [0,0,1], Cyan [0,1,1], and Black [0,0,0].

Box 2: Studying the origin of Black using the primary and one secondary (admixed) color populations Following criticism on the sampling scheme used to study the origin of Black (Box 1), the redoubtable Black-is-Red group genotyped Cyan. Using even sample sizes, they demonstrated that Black is closer to Red (*D*_*Black-Red*_ = 0.46) (Fig. 8A), where *D* is the Euclidean distance between the samples over all three PCs (short distances indicate high similarity). The Black-is-Green school criticized their findings on the grounds that their Cyan samples were biased and their results do not apply to the broad Black cohort. They also reckoned that the even sampling scheme favored Red because Blue is related to Cyan through shared language and customs. The Black-is-Red group responded by enriching their cohort in Cyan and Black (*n*_*Cyan*_, *n*_*Black*_ = 1000) and provided even more robust evidence that Black is Red (*D*_*Black-Red*_ = 0.12) (Fig. 8B). However, the Black-is-Green camp dismissed these findings. Conscious of the effects of admixture, they retained only the most homogeneous Green and Cyan (*n*_*Green*_*, n*_*Cyan*_ = 33), genotyped new Blue and Black (*n*_*Blue*_*, n*_*Black*_ = 400), and analyzed them with the published Red cohort (*n*_*Red*_ = 100). The Black-is-Green results supported their hypothesis that Black is Green (*D*_*Black-Green*_ = 0.27) and that Cyan shared a common origin with Blue (*D*_*Blue-Green*_ = 0.27) (Fig. 8C) and should thereby be considered an admixed Blue population. Unsurprisingly, the Black-is-Red group claimed that these results were due to the under-representation of Black since when they oversampled Black, PCA supported their findings (Fig. 8A). In response, the Black-is-Green school maintained even sample sizes for Cyan, Blue, and Green (*n*_*Blue*_*, n*_*Green*_, *n*_*Cyan*_ = 33) and enriched Black and Red (*n*_*Red*_, *n*_*Black*_ = 100). Not only did their results (*D*_*Black-Green*_ = 0.63 < *D*_*Black-Red*_ = 0.89) support their previous findings, but they also demonstrated that Green and Blue completely overlapped, presumably due to their shared co-ancestry, and that together with Cyan (*D*_*Cyan-Green*_ = 0.63 < *D*_*Cyan-Red*_ = 1.09) (Fig. 8B,D) they represent an antique color clade. They explained that these color populations only appeared separated due to genetic drift. However, they still retained sufficient cryptic genetic information that PCA can uncover if the correct sampling scheme is used. Further analyses by the other groups contested these findings (Supplementary Fig. S5A-D). Among else, it was argued that Black is a Green–Red admixed group (Supplementary Fig. S5C) and that Black and Cyan were the ancestors of Blue and Green (Supplementary Fig. S5D).

### The case of a multi-admixed population

The question of how analyzing admixed groups with multiple ancestral populations affects the findings for unmixed groups is illustrated through a typical study case in Box 3.

To understand how PCA can be misused to study multiple mixed populations, we will investigate other PCA applications to study AJs. Such analyses have a thematic intepretation, where the clustering of AJ samples is evidence of a shared Levantine origin, e.g., Refs.^12,13^, that “short” distances between AJs and Levantines indicate close genetic relationships in support of a shared Levantine past, e.g., Ref.^12^, whereas the “short” distances between AJs and Europeans are evidence of admixture^13^. Finally, as a rule, the much shorter distances between AJs and the Caucasus or Turkish populations, observed by all recent studies, were ignored^12,13,47,48^. Bray et al.^48^ concluded that not only do AJs have a “more southern origin” but that their alignment with Central Europeans is “consistent with migration to this region”. In these studies, "short" and “between” received a multitude of interpretations. For example, Gladstein and Hammer's^53^ PCA plot that showed AJs in the extreme edge of the plot with Bedouins and French in the other edges was interpreted as AJs clustering “tightly between European and Middle Eastern populations”. The authors interpreted the lack of “outliers” among AJs (which were never defined) as evidence of common AJ ancestry.

**Figure 9:**
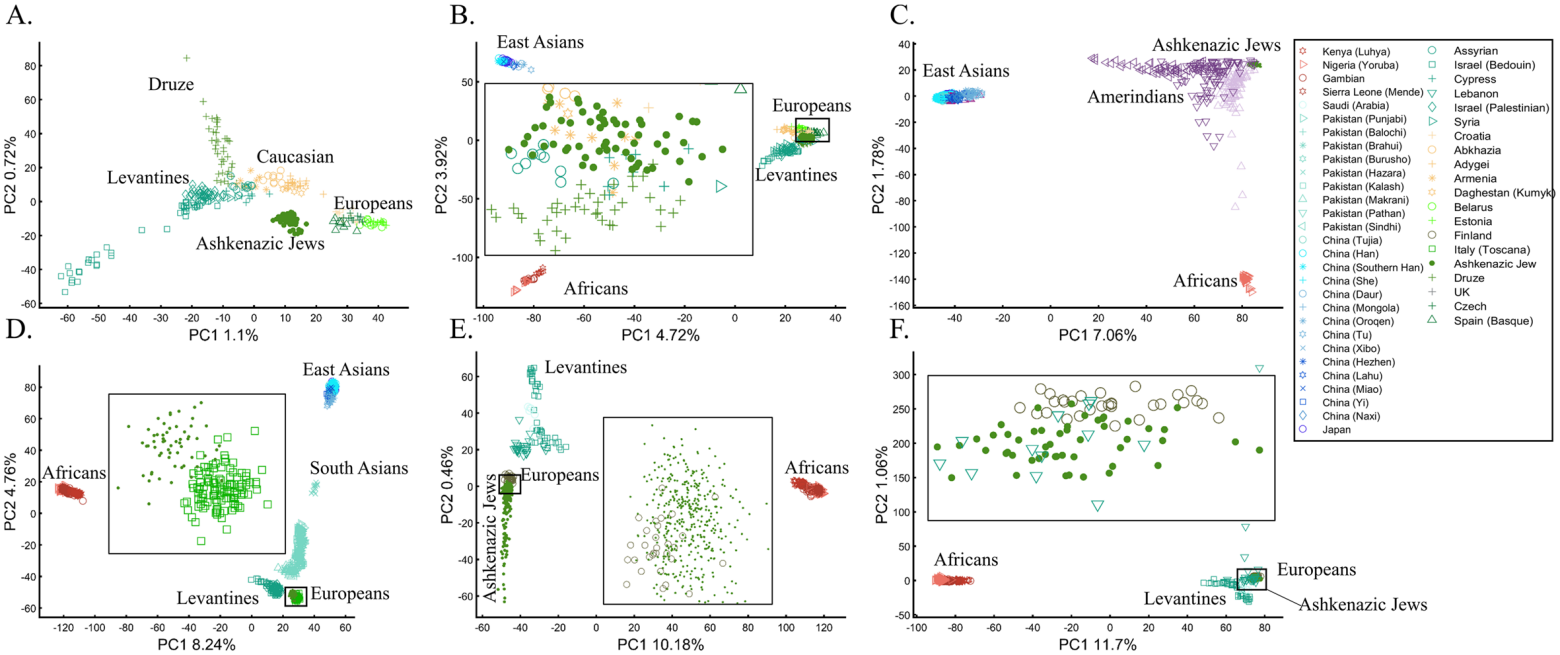
An in-depth study of the origin of AJs using PCA in relation to Africans (Af), Europeans (Eu), East Asians (Ea), Amerindians (Am), Levantines (Le), and South Asians (Sa). (**A**) *n*_*Eu*_ = 159; *n*_*AJ*_ = 60; *n*_*Le*_ = 82, (**B**) *n*_*Af*_ = 30; *n*_*Eu*_ = 159; *n*_*Ea*_ = 50; *n*_*AJ*_ = 60; *n*_*Le*_ = 60, (**C**) *n*_*Af*_ = 30; *n*_*Ea*_ = 583; *n*_*AJ*_ = 60; *n*_*Am*_ = 255; (**D**) *n*_*Af*_ = 200; *n*_*Eu*_ = 115; *n*_*Ea*_ = 200; *n*_*AJ*_ = 60; *n*_*Le*_ = 235; *n*_*Sa*_ = 88, (**E**) *n*_*Af*_ = 200; *n*_*Eu*_ = 30; *n*_*AJ*_ = 400, *n*_*Le*_ = 80 (**F**) *n*_*Af*_ = 200; *n*_*Eu*_ = 30; *n*_*AJ*_ = 50; *n*_*Le*_ = 160. Large square indicate insets.

Following the rationale of these studies, it is easy to show how PCA can be orchestrated to yield a multitude origins for AJs. We replicated the observation that AJs are “population isolate,” i.e., AJs form a distinct group, separated from all other populations (Fig. 9A), and are thereby genetically distinguishable^47^. We also replicated the most common yet often-ignored observation, that AJs cluster tightly with Caucasus populations (Fig. 9B). We next produced novel results where AJs cluster tightly with Amerindians due to the north Eurasian or Amerindian origins of both groups (Fig. 9C). We can also show that AJs cluster much closer to South Europeans than Levantines (Fig. 9D), and overlap Finns entirely, in solid evidence of AJ’s ancient Finnish origin (Fig. 9E). Last, we wish to refute our previous finding and show that only half of the AJs are of Finnish origin. The remaining analysis supports the lucrative Levantine origin (Fig. 9F)—a discovery touted by all the previous reports though never actually shown. Excitingly enough, the primary PCs of this last Eurasian Finnish-Levantine mixed origin depiction explained the highest amount of variance. An intuitive interpretation of those results is a recent migration of the Finnish AJs to the Levant, where they experienced high admixture with the local Levantine populations that altered their genetic background. These examples demonstrate that PCA plots generate nonsensical results for the same populations and no a posteriori knowledge.

**Figure 10:**
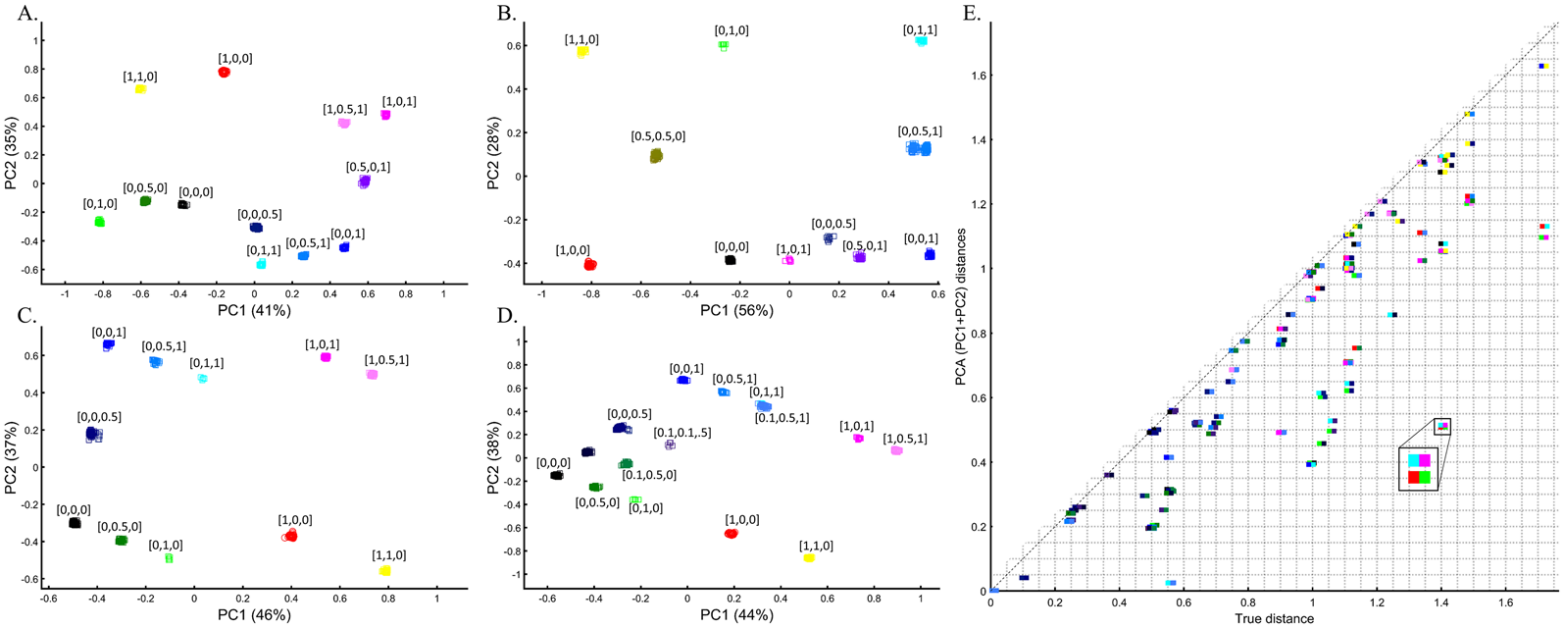
PCA with the primary and multiple mixed color populations. (**A**) *n*_*all*_ = 50, (**B**) *n*_*all*_ = 50 or 10, (**C**,**D**) *n*_*All*_ = [50, 5, 100, or 25]. Scatter plots show the top two PCs. Colors codes are shown. (**E**) The difference between the true distances calculated over a 3D plane between every color population pair (shown side by side) from (**D**) and their Euclidean distances calculated from the top two PCs. Pairs whose PC distances from each other reflect their true 3D distances are shown along the x = y dotted line. One of the largest PCA distortions is the distances between the Red and Green populations (inset). The true Red-Green distance is 1.41 (x-axis), but the PCA distance is 0.5 (y-axis).

Box 3: Studying the origin of Black using the primary and multiple mixed colors The value of using mixed color populations to study origins prompted new analyses using even (Fig. 10A) and variable sample sizes (Fig. 10B–D). Using this novel sampling scheme, the Black-is-Green school reaffirmed that Black is the closest to Green (Fig. 10A, 10C, and 10D) in a series of analyses, but using a different cohort yielded a novel finding that Black is closest to Pink (Fig. 10B).

The extent to which PCA distances obtained by the top two PCs reflect the true distances among color population pairs is shown in Fig. 10E. PCA distorted the distances between most color populations, but the distortion was uneven among the pairs, and while a minority of the pairs are correctly projected via PCA, most are not. Identifying which pairs are correctly projected is impossible without a priori information. For example, some shades of blue and purple were less biased than similar shades. We thereby show that PCA inferred distances are biased in an unpredicted manner and thereby uninformative for clustering.

### The case of multiple admixed populations without “unmixed” populations

Unlike stochastic models that possess inherent randomness, PCA is a deterministic process, a property that contributes to its perceived robustness. To explore the behavior of PCA, we tested whether the *same computer code* can produce similar or different results when the only variable that changes is the standard randomization technique used throughout the paper to generate the individual samples of the color populations (to avoid clutter).

**Figure 11:**
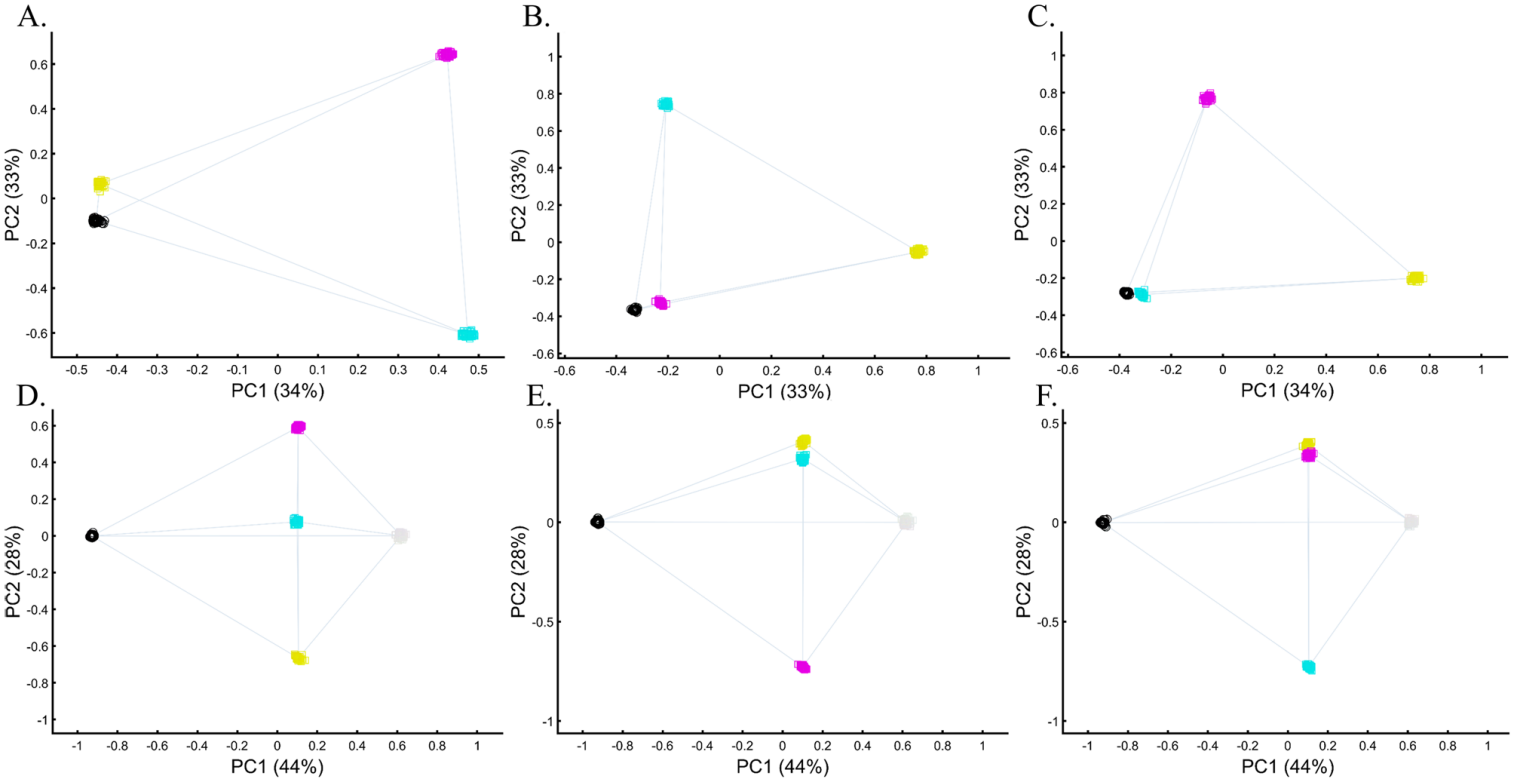
Studying the effects of minor sample variation on PCA results using color populations (*n*_*all*_ = 50). (**A**–**C**) Analyzing secondary colors and Black. (**D**–**E**) Analyzing secondary colors, White, and Black. Scatter plots show the top two PCs. Colors include Cyan [0,1,1], Purple [1,0,1], Yellow [1,1,0], White [1,1,0], and Black [0,0,0].

We evaluated two color sets. In the first set, Black was the closest to Yellow (Fig. 11A), Purple (Fig. 11C), and Cyan (Fig. 11D,E). When adding White, in the second set, Black behaved as an outgroup as the distances between the secondary colors largely deviated from the expectation and produced false results (Fig. 11D–F). These results illustrate the sensitivity of PCA to tiny changes in the dataset, unrelated to the populations or the sample sizes.

**Figure 12:**
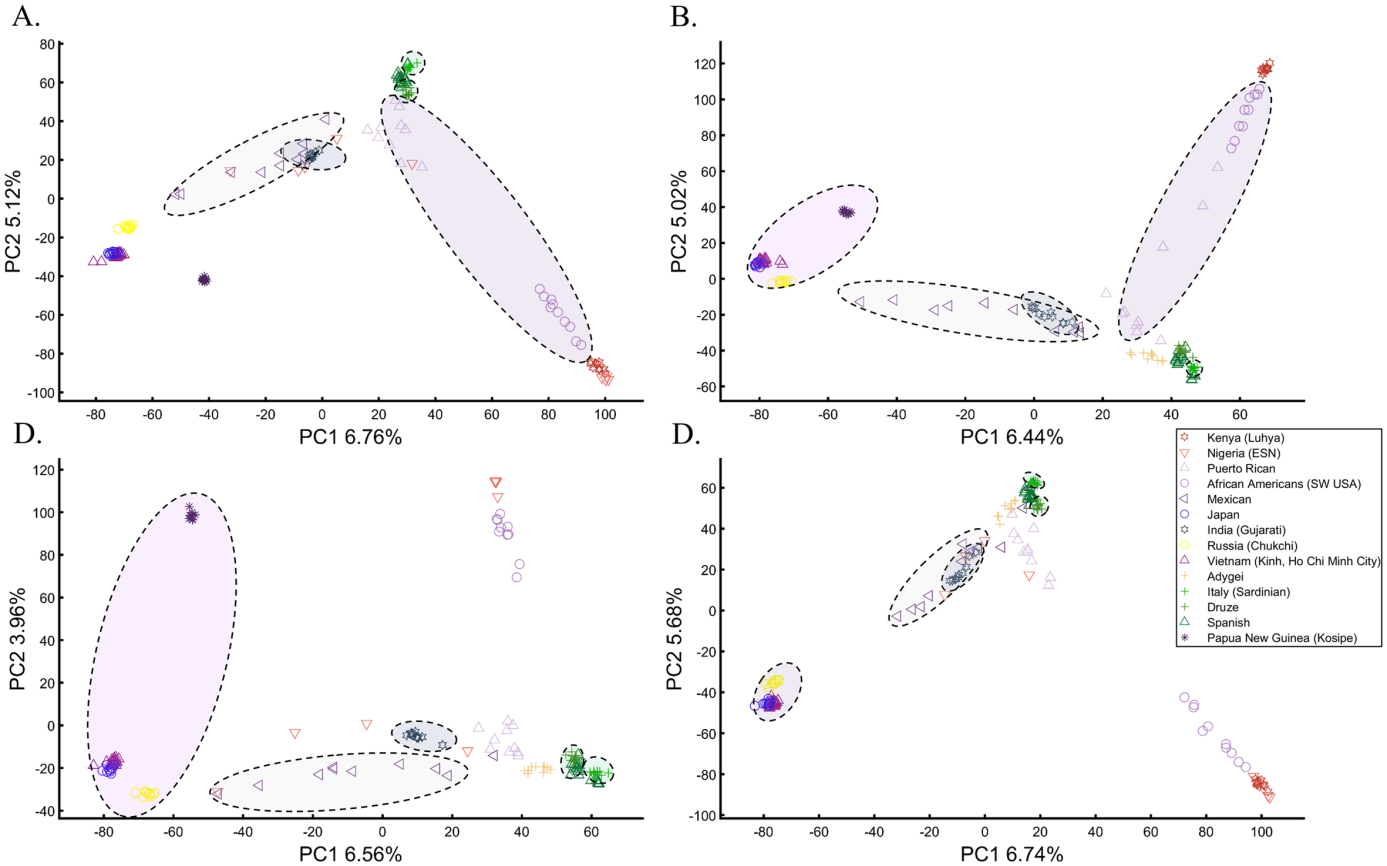
Studying the effect of sampling on PCA results. A cohort of 16 worldwide populations (see legend) was selected. In each analysis, a random population was excluded. Populations were represented by random samples (*n* = 10). The clusters highlight the most notable differences.

To explore this effect on human populations, we curated a cohort of 16 populations. We carried out PCA on ten random individuals from 15 random populations. We show that these analyses result in spurious and conflicting results (Fig. 12). Puerto Ricans, for instance, clustered close to Europeans (A), between Africans and Europeans (B), close to Adygei (C), and close to Europe and Adygei (D). Indians clustered with Mexicans (A, B, and D) or apart from them (C). Mexicans themselves cluster with (A and D) or without (B and C) Africans. Papuans and Russians cluster close (B) or afar (C) from East Asian populations. More robust clustering was observed for East Asians, Caucasians, and Europeans, as well as Africans. However, these were not only indistinguishable from the less robust clustering but also failed to replicate over multiple runs (results not shown). These examples show that PCA results are unpredictable and irreproducible even when 94% of the populations are the same. Note that the proportion of explained variance was similar in all the analyses, demonstrating that it is not an indication of accuracy or robustness.

We found that although a deterministic process, PCA behaves unexpectedly, and minor variations can lead to an ensemble of different outputs that appear stochastic. This effect is more substantial when continental populations are excluded from the analysis.

### The cases of case–control matching and GWAS

**Figure 13:**
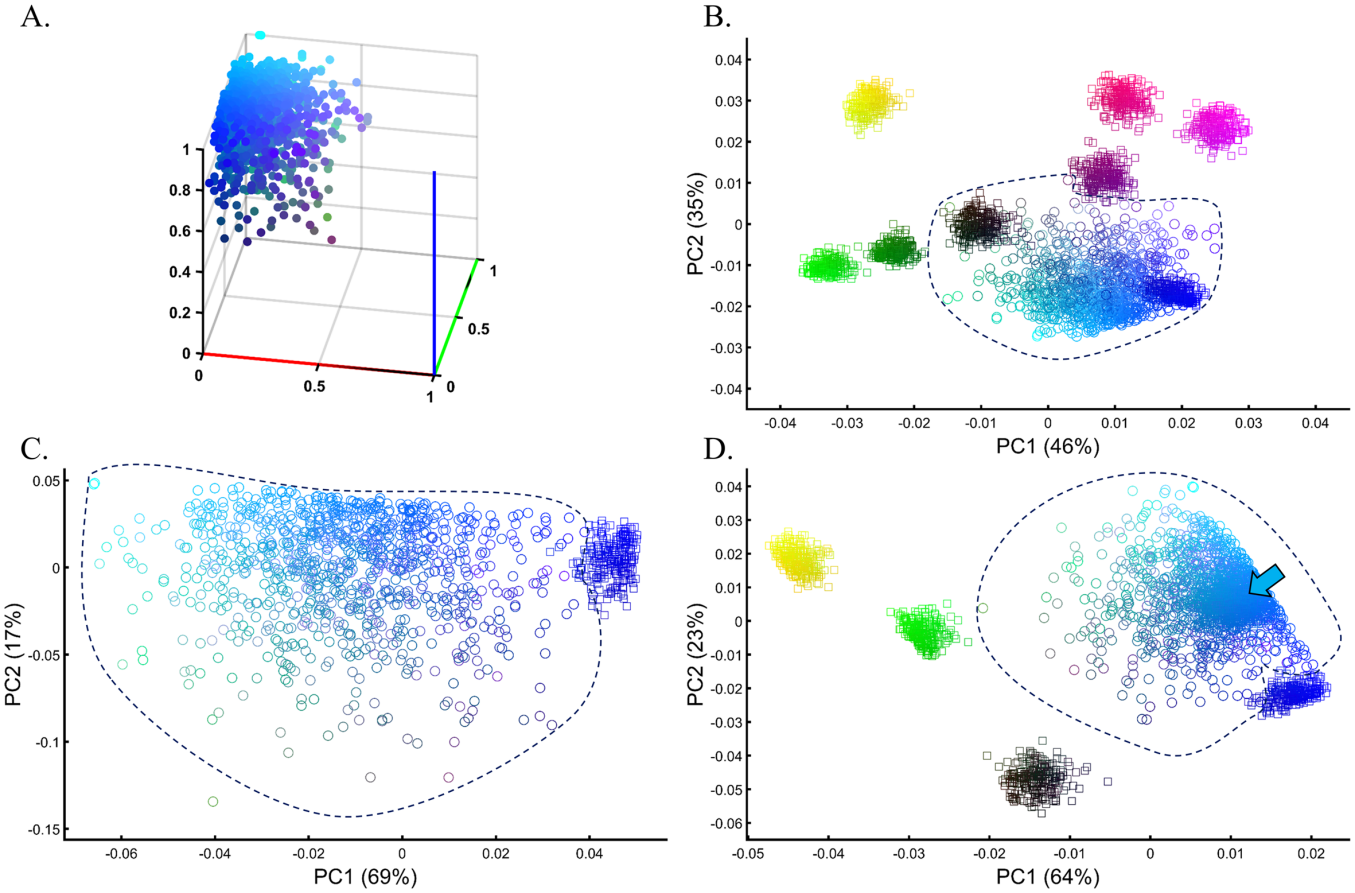
Evaluating the accuracy of PCA clustering for a heterogeneous test population in a simulation of a GWAS setting. (**A**) The true distribution of the test Cyan population (*n* = 1000). (**B**) PCA of the test population with eight even-sized (*n* = 250) samples from reference populations. (**C**) PCA of the test population with Blue from the previous analysis shows a minimal overlap between the cohorts. (**D**) PCA of the test population with five even-sized (*n* = 250) samples from reference populations, including Cyan (marked by an arrow). Colors (**B**) from top to bottom and left to right include: Yellow [1,1,0], light Red [1,0,0.5], Purple [1,0,1], Dark Purple [0.5,0,0.5], Black [0,0,0], dark Green [0,0.5,0], Green [0,1,0], and Blue [1,0,0].

Samples of unknown ancestry or self-reported ancestry are typically identified by applying PCA to a cohort of test samples combined with reference populations of known ancestry (e.g., 1000 Genomes), e.g., Refs.^22,54–56^. To test whether using PCA to identify the ancestry of an unknown cohort with known samples is feasible, we simulated a large and heterogeneous Cyan population (Fig. 13A, circles) of self-reported Blue ancestry. Following a typical GWAS scheme, we carried out PCA for these individuals and seven known and distinct color populations. PCA grouped the Cyan individuals with Blue and Black individuals (Fig. 13B), although none of the Cyan individuals were Blue or Black (Fig. 13A), as a different PCA scheme confirmed (Fig. 13C). A case–control assignment of this cohort to Blue or Black based on the PCA result (Fig. 13B) produced poor matches that reduced the power of the analysis. When repeating the analysis with different reference populations (Fig. 13D), the simulated individuals exhibited minimal overlap with Blue, no overlap with Black, and overlapped mostly with the Cyan reference population present this time. We thereby showed that the clustering with Blue and Black is an artifact due to the choice of reference populations. In other words, the introduction of reference populations with mismatched ancestries respective to the unknown samples biases the ancestry inference of the latter.

**Figure 14:**
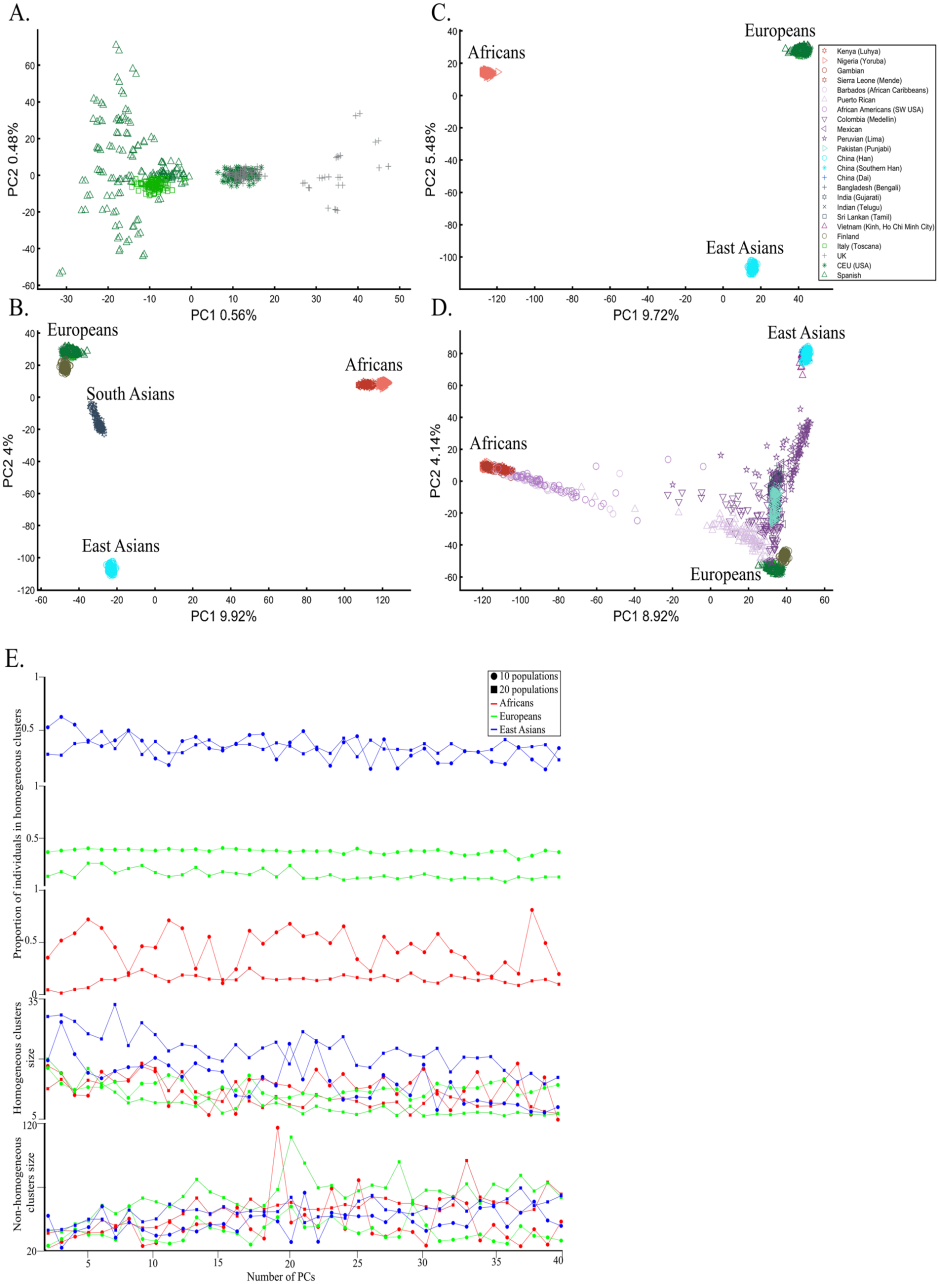
Evaluating the cluster homogeneity of European samples. PCA was applied to the four European populations (Tuscan Italians [TSI], Northern and Western Europeans from Utah [CEU], British [GBR], and Spanish [IBS]) alone (**A**), together with an African and Asian population (**B**), as well as South Asian population (**C**), and finally with all the 1000 Genomes Populations (**D**). (**E**) Evaluating the usefulness of PCA-based clustering. The bottom two plots show the sizes of non-homogeneous and homogeneous clusters, and the top three plots show the proportion of individuals in homogeneous clusters. Each plot shows the results for 10 or 20 random African, European, or Asian populations for the same PCs (*x*-axis).

We next asked whether PCA results can group Europeans into homogeneous clusters. Analyzing four European populations yielded 43% homogeneous clusters (Fig. 14A). Adding Africans and Asians and then South Asian populations decreased the European cluster homogeneity to 14% and 10%, respectively (Fig. 14B,C). Including the 1000 Genome populations, as customarily done, yielded 14% homogeneous clusters (Fig. 14D). Although the Europeans remained the same, the addition of other continental populations resulted in a three to four times decrease in the homogeneity of their clusters.

The number of PCs analyzed in the literature ranges from 2 to, at least, 280^35^, which raises the question of whether using more PCs increases cluster homogeneity or is another *cherry-picking* strategy. We calculated the cluster homogeneity for different PCs for either 10 or 20 African (*n*_*10*_ = 337, *n*_*20*_ = 912), Asian (*n*_*10*_ = 331, *n*_*20*_ = 785), and European (*n*_*10*_ = 440, *n*_*20*_ = 935) populations of similar sample sizes (Fig. 14E). Even in this favorable setting that included only continental populations, on average, the homogeneous clusters identified using PCA were significantly smaller than the non-homogeneous clusters (*µ*_*Homogeneous*_ = 12.5 samples; *σ*_*Non-homogeneous*_ = 42.6 samples; *µ*_*Homogeneous*_ = 12.5 samples; *µ*_*Non-homogeneous*_ = 42.6 samples; Kruskal–Wallis test [*n*_*Homogeneous*_ = *n*_*Non-homogeneous*_ = 238 samples, *p* = 1.95 × 10^–75^, Chi-square = 338]) and included a minority of the individuals when 20 populations were analyzed. Analyzing higher PCs decreased the size of the homogeneous clusters and increased the size of the non-homogeneous ones. The maximum number of individuals in the homogeneous clusters fluctuated for different populations and sample sizes. Mixing other continental populations with each cohort decreased the homogeneity of the clusters and their sizes (results now shown). Overall, these examples show that PCA is a poor clustering tool, particularly as sample size increases, in agreement with Elhaik and Ryan^57^, who reported that PCA clusters are neither genetically nor geographical homogeneous and that PCA does not handle admixed individuals well. Note that the cluster homogeneity in this limited setting should not be confused with the amount of variance explained by additional PCs.

**Figure 15:**
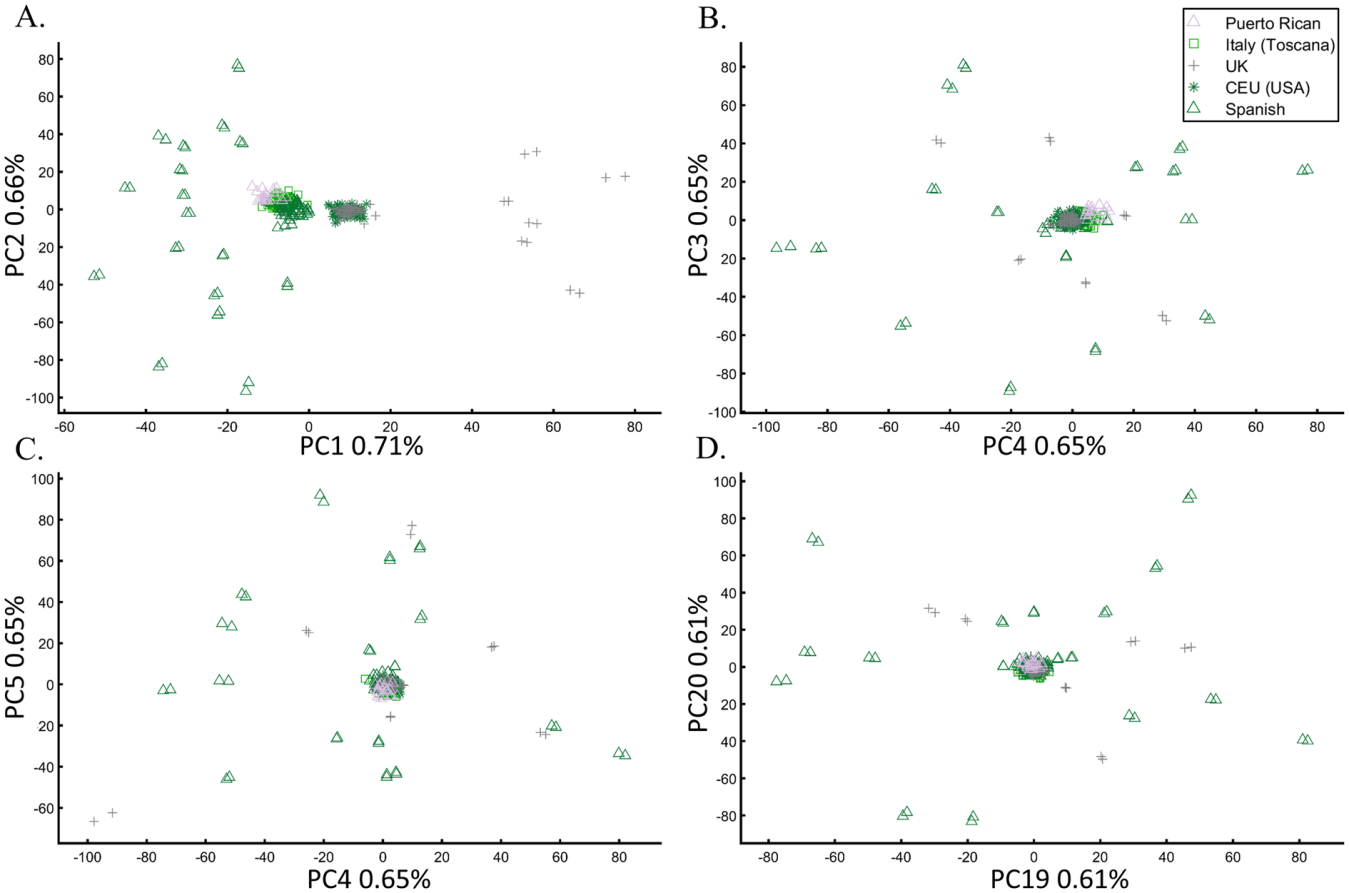
PCA of 20 Puerto Ricans and 300 random Europeans from the 1000 Genomes. The results are shown for various PCs.

To further assess whether PCA clustering represents shared ancestry or biogeography, two of the most common applications of PCA, e.g., Ref.^22^, we applied PCA to 20 Puerto Ricans (Fig. 15) and 300 Europeans. The Puerto Ricans clustered indistinguishably with Europeans (by contrast to Fig. 12) using the first two and higher PCs (Fig. 15). The Puerto Ricans represented over 6% of the cohort, sufficient to generate a stratification bias in an association study. We tested that by randomly assigning case–control labels to the European samples with all the Puerto Ricans as controls. We then generated causal alleles to the evenly-sized cohorts and computed the association before and after PCA adjustment. We repeated the analysis with randomly assigned labels to all the samples. In all our 12 case–control analyses, the outcome of the PCA adjustment for 2 and 10 PCs were worse than the unadjusted results, i.e., PCA adjusted results had more false positives, fewer true positives, and weaker *p*-values than the unadjusted results (Supplementary Text 3).

**Figure 16:**
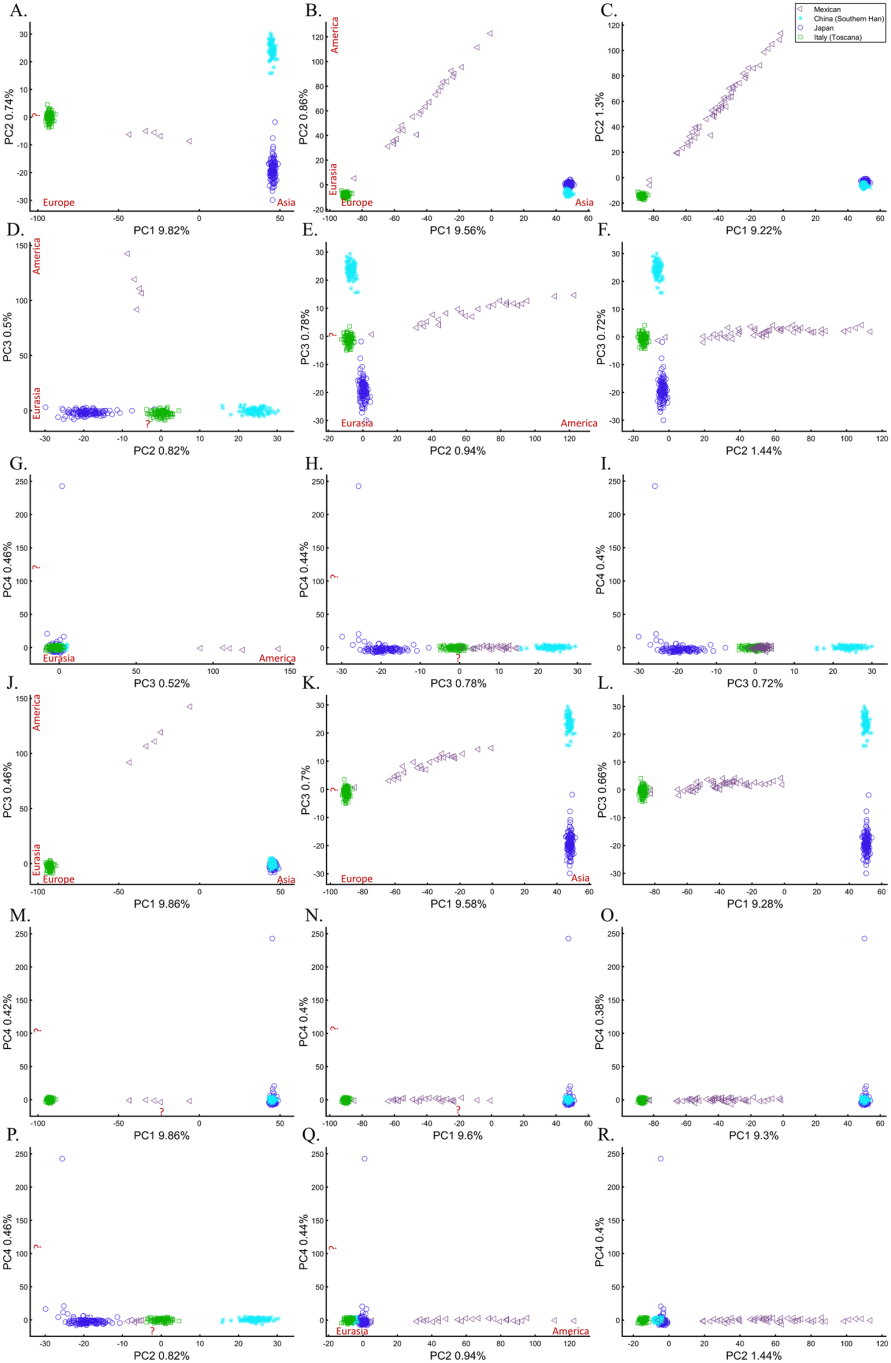
The effect of varying the number of Mexican–American on the inference of genetic distances between Chinese and Japanese using various PCs. We analyzed a fixed number of 135 Han Chinese (CHB), 133 Japanese (JPT), 115 Italians (TSI), and a variable number of Mexicans (MXL), including 5 (left column), 25 (middle column), and 50 (right column) individuals over the top four PCs. We found that the overlap between Chinese and Japanese in PC scatterplots, typically used to infer genomic distances, was unexpectedly conditional on the number of Mexican in the cohort. We noted the meaning of the axes of variation whenever apparent (red). The right column had the same axes of variations as the middle one.

We next assessed whether the distance between individuals and populations is a meaningful biological or demographic quantity by studying the relationships between Chinese and Japanese, a question of major interest in the literature^58,59^. We already applied PCA to Chinese and Japanese, using Europeans as an outgroup (Supplementary Fig. S2.4). The only element that varied in the following analyses was the number of Mexicans as the second outgroup (5, 25, and 50). We found that the proportion of homogeneous Japanese and Chinese clusters dropped from 100% (Fig. 16A) to 93.33% (Fig. 16B) and 40% (Fig. 16C), demonstrating that the genetic distances between Chinese and Japanese depend entirely on the number of Mexicans in the cohort rather than the actual genetic relationships between these populations as one may expect.

Some authors consider higher PCs informative and advise considering these PCs alongside the first two. In our case, however, these PCs were not only susceptible to bias due to the addition of Mexicans but also exhibited the exact opposite pattern observed by the primary PCs (e.g., Fig. 16G–I). It has also been suggested that in datasets with ancestry differences between samples, axes of variation often have a geographic interpretation^10^. Accordingly, the addition of Mexicans altered the order of axes of variation between the cases, making the analysis of additional PCs valuable. We demonstrate that this is not always the case. Excepting PC1, over 60% of the axes had no geographical interpretation or an incorrect one. An a priori knowledge of the current distribution of the population was essential to differentiate these cases. The addition of the first 20 Mexicans replaced the second axis of variation (initially undefined) with a third axis (Eurasia-America) in the middle and right columns and resulted in a minor decline of ~ 5% of the homogeneous clusters. Adding 25 Mexicans to the second cohort did not affect the axes, but the proportion of homogeneous clusters declined by 66%. The axes changes were unexpected and altered the interpretation of PCA results. Such changes were not detectable without an a priori knolwedge.

These results demonstrate that (1) the observable distances (and thereby clusters) between populations inferred from PCA plots (Figs. 14, 15, 16) are artifacts of the cohort and do not provide meaningful biological or historical information, (2) that distances betewen samples can be easily manipulated by the experimenter in a way that produces unpredictable results, (3) that considering higher PCs produces conflicting patterns, which are difficult to reconcile and interpret, and (4) that our extensive “exploration” of PCA solutions to Chinese and Japanese relationships using 18 scatterplots and four PCs produced no insight. It is easy to see that the multitude of conflicting results, allows the experimenter to select the favorable solution that reflects their a priori knowledge.

### The case of projections

**Figure 17:**
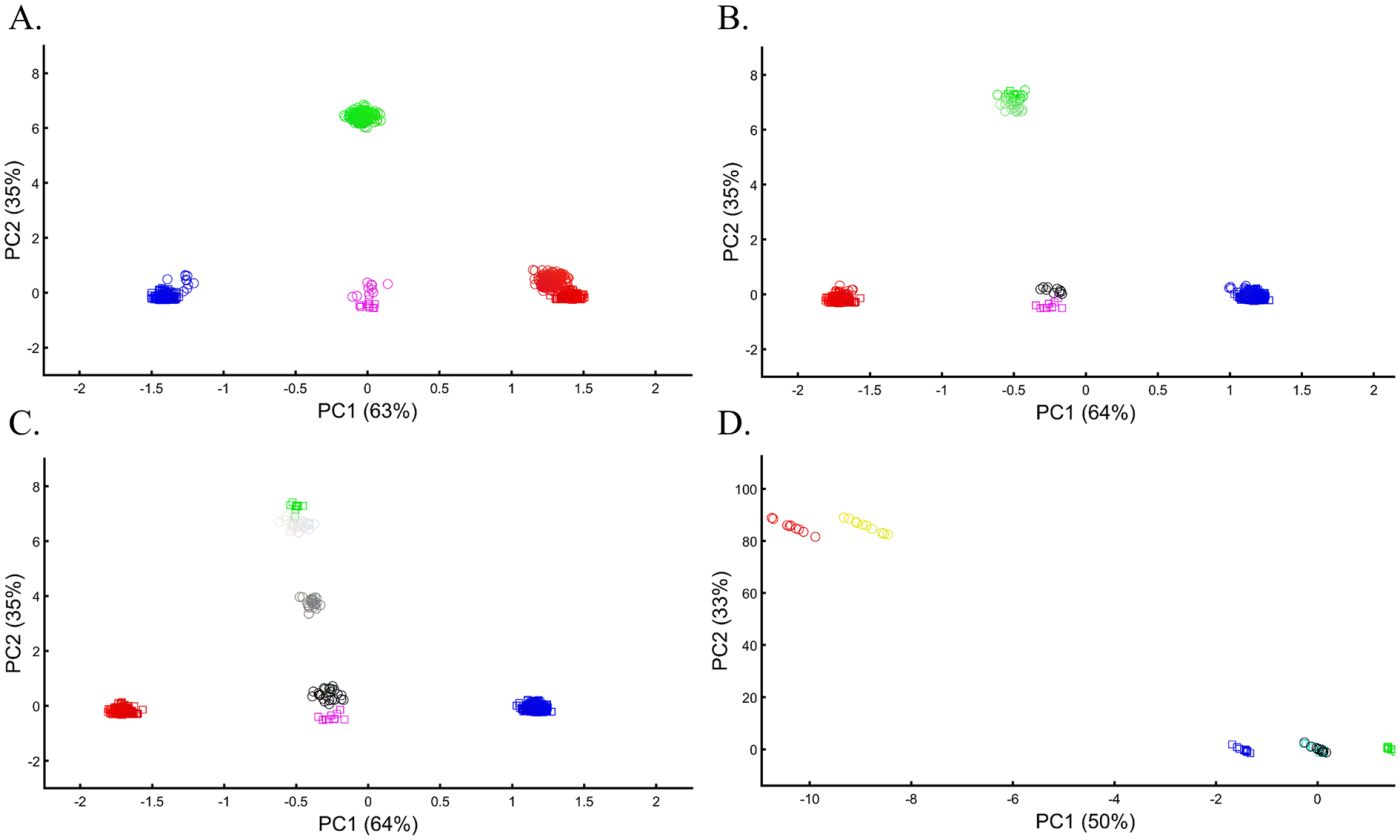
Examining the accuracy of PCA projections. The PCA results of one dataset (circles) were projected onto another (squares). In (**A**), testing the case of varying sample sizes between the first (*n*_*Red*_ = 200, *n*_*Green*_ = 10, *n*_*Blue*_ = 200, *n*_*Purple*_ = 10) and second (*n*_*Red*_ = 200, *n*_*Green*_ = 200, *n*_*Blue*_ = 10, *n*_*Purple*_ = 10) datasets, where in the second dataset, colors varied a little (e.g., [1,0,0] → [1,0.1,0.1]). In (**B**–**D**), the sample size varied (10 ≤ *n* ≤ 300) for both datasets. Colors include Red [1,0,0], Green [0,1,0], light Green [1,0.2,1], Cyan [0,1,1], Blue [0,0,1], Purple [1,0,1], Yellow [1,1,0], Grey [0.5,0.5,0.5], White [1,1,1], and Black [0,0,0].

Incorporating precalculated PCA is done by projecting the PCA results calculated for the first dataset onto the second one, e.g., Ref.^17^. Here, we tested the accuracy of this approach by projecting one or more color populations onto precalculated color populations that may or may not match the projected ones. The accuracy of the results was dependent on the identity of the populations of the two cohorts. When the same populations were analyzed, they overlapped (Fig. 17A), but when unique populations were found in the two datasets, PCA created misleading matches (Figs. 17B–D). In the latter case, and when the sample sizes were uneven (Fig. 17C), the projected samples formed clusters with the wrong populations, and their positioning in the plot was incorrect. Overall, we found that PCA projections are unreliable and misleading, with correct outcomes indistinguishable from incorrect ones.

**Figure 18:**
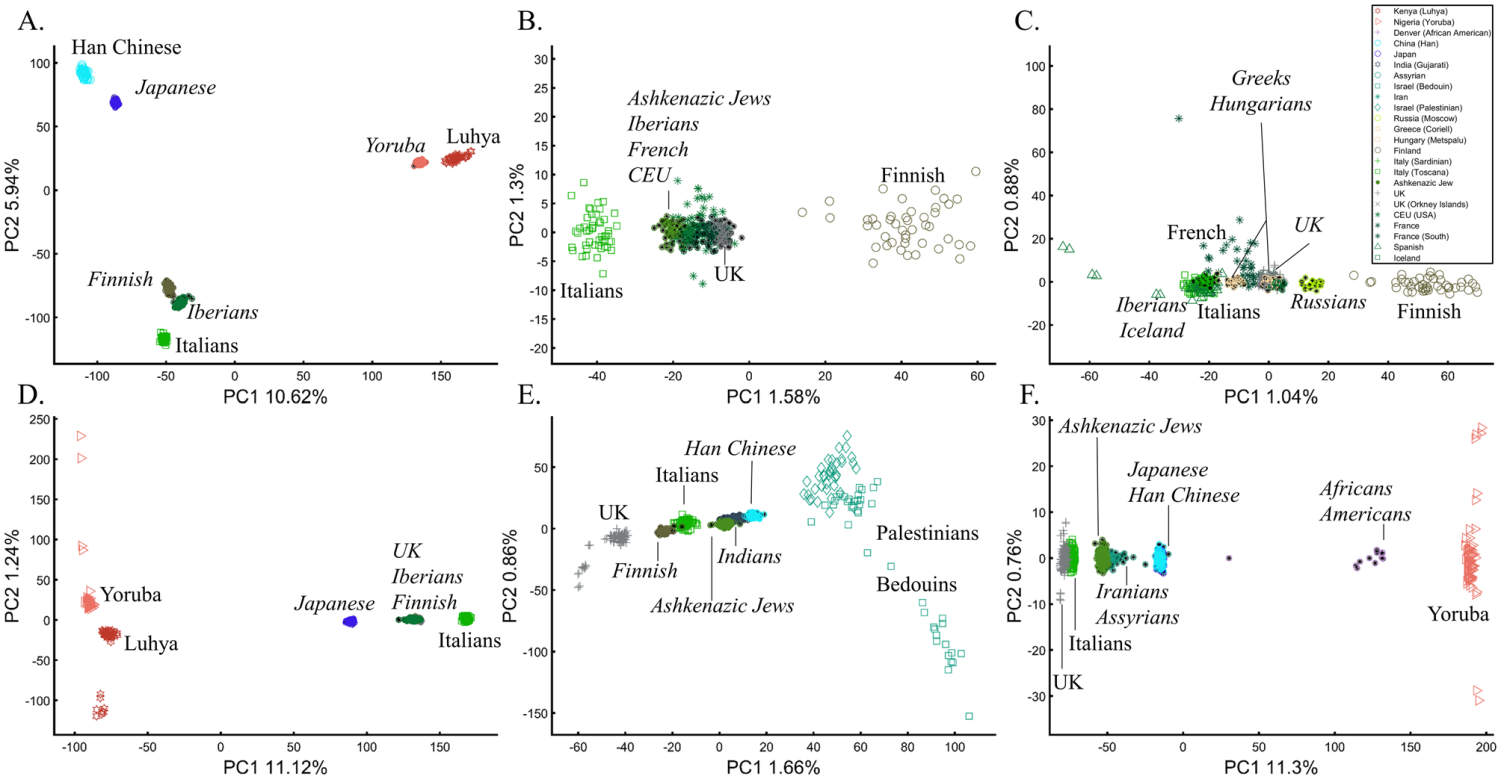
PCA projections of populations (italic and black star inside the shape) onto base populations with even-sized sample (*n* = 50, unless noted otherwise) (regular font). In (**A**) *n*_*projected*_ = 100, (**B**) *n*_*projected*_ = 50, (**C**) *n*_*projected*_ = 20, (**D**) *n*_*projected*_ = 100, (**E**) *n*_*projected*_ = 80 and *n*_*projected*_ = 100, and (**F**) 80 ≤ *n*_*projected*_ ≤ 100 and 12 ≤ *n*_*projected*_ ≤ 478.

To evaluate the reliability of projections for human populations, we tested whether the projected populations cluster with their closest groups and to what extent these results can be manipulated. We found that populations can be shown to correctly align with continental populations when the base (or test) populations and the projected populations are very similar (Fig. 18A), which gives us confidence in the accuracy of PCA projections. However, even in the simplest scenario of using three continental populations, it is unclear how to interpret the overlap between the base and projected populations since the Spanish would not be considered genetically closer to Finns than Italians, as suggested by PCA. In another simple scenario, where Europeans are projected onto other Europeans, distinct populations like AJs, Iberians, French, CEU, and British overlap entirely (Fig. 18B), whereas Finns and Italians were separate. Not only do the results share no apparent resemblance to the geographical distribution, but they also produce conflicting information as to the genetic distances between these populations—two properties that PCA enthusiastics claim it represents. Adding more populations, even if only to the projected populations, contributes to further distortions with previously distinct populations (Fig. 18B) now clustering (Fig. 18C). In a different dataset, projecting Japanese onto a base dataset of Africans and Europeans places them as an admixed African-European population. The projected Finns cluster with other Europeans (Fig. 18D), at odds with the previous results (Fig. 18B) that singled them out.

To test the behavior of PCA when projecting populations different from the base populations, we projected Chinese, Finns, Indians, and AJs onto Levantine and two European populations (Fig. 18E). The results imply that the Chinese and AJs are of an Indian origin originating from a European-Levantine mix. Replacing Levantines with Africans does not stabilize the projected results (Fig. 18F). Now the projected Chinese and Japanese overlap, and AJs cluster with Iranians.

Overall, our results show that it is unfeasible to rely on PCA projections, particularly in studies involving different populations, as is commonly done. Even when the projected populations are identical to the base ones, the base and projected populations may or may not overlap.

### The case of ancient DNA

PCA is the primary tool in paleogenomics, where ancient samples are initially identified based on their clustering with modern or other ancient samples. Here, a wide variety of strategies is employed. In some studies, ancient and modern samples are combined^60^. In other studies, PCA is performed separately for each ancient individual and “particular reference samples”, and the PC loadings are combined^61^. Some authors projected present-day human populations onto the top two principal components defined by ancient hominins (and non-humans)^62^. The most common strategy is to project ancient DNA onto the top two principal components defined by modern-day populations^14^. Here, we will investigate the accuracy of this strategy.

**Figure 19:**
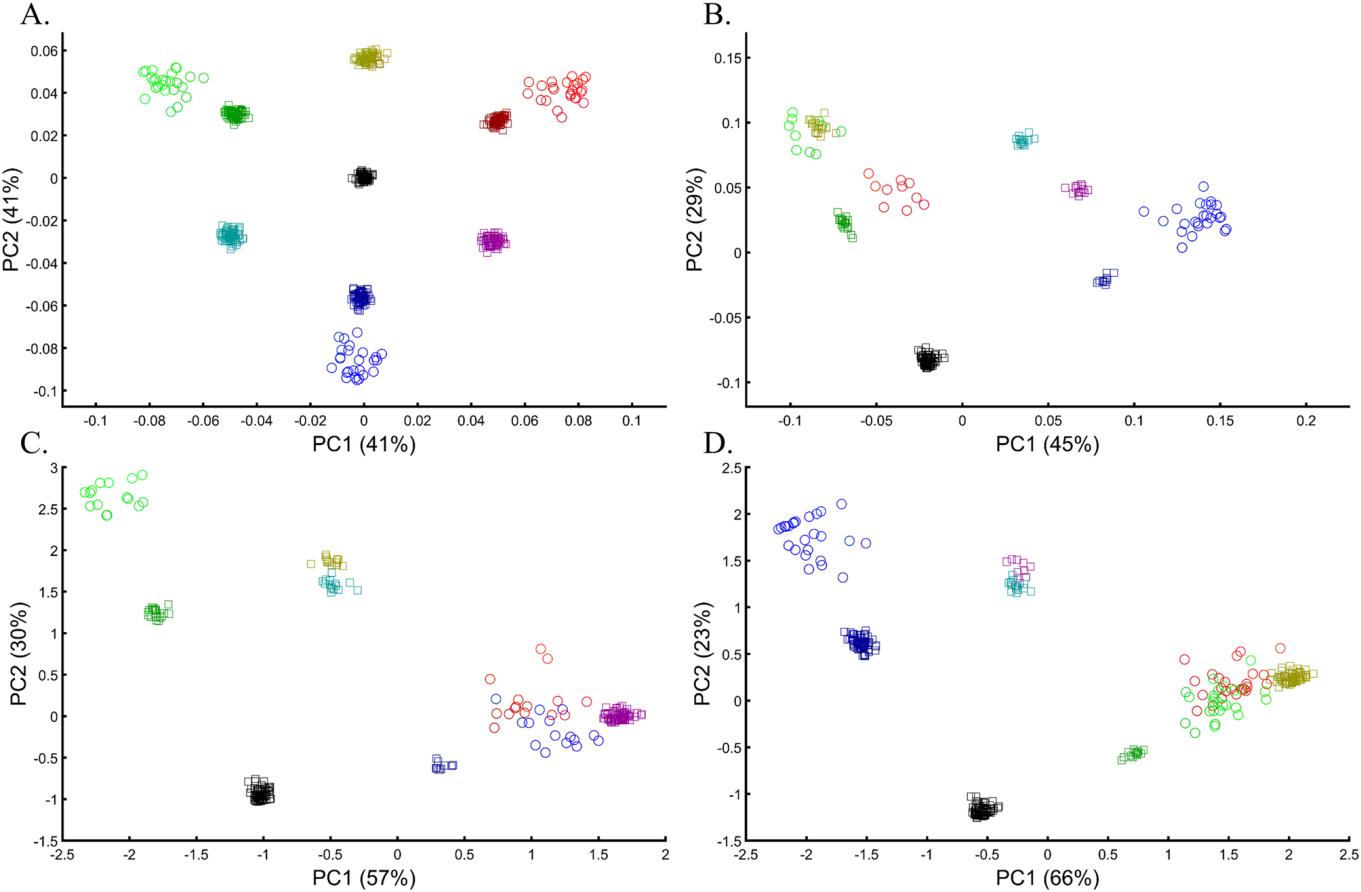
Merging PCA of “ancient” (circles) and “modern” (squares) color populations using two approaches. First, PCA is calculated separately on the two datasets, and the results are plotted together (**A**,**B**). Second, PCA results of “ancient” populations are projected onto the PCs of the “modern” ones (**C**,**D**). In (**A**), even-sized samples from “ancient” (*n* = 25) and “modern” (*n* = 75) color populations are used. In (**B**), different-sized samples from “ancient” (10 ≤ *n* ≤ 25) and “modern” (10 ≤ *n* ≤ 75) populations are used. In (**C**) and (**D**), different-sized samples from “ancient” (10 ≤ *n* ≤ 75) are used alongside even-sized samples from “modern” populations: (**C**) (*n* = 15) and (**D**) *n* = 25. Colors include Red [1,0,0], dark Red [0.6,0,0], Green [0,1,0], dark Green [0,0.6,0], Blue [0,0,1], dark Blue [0,0,0.6], light Cyan [0,0.6,0.6], light Yellow [0.6,0.6,0], light Purple [0.6,0,0.6], and Black [0,0,0].

Since ancient populations show more genetic diversity than modern ones^14^, we defined “ancient colors” (*a*) as brighter colors whose allele frequency is 0.95 with an SD of 0.05 and “modern colors” (*m*) as darker colors whose allele frequency is 0.6 with an SD of 0.02. Two approaches were used in analyzing the two datasets: calculating PCA separately for the two datasets and presenting the results jointly (Fig. 19A,B), and projecting the PCA results of the “ancient” populations onto the “modern” ones (Fig. 19C,D). In both cases, meaningful results would show the ancient colors clustering close to their modern counterparts in distances corresponding to their true distances.

These are indeed the results of PCA when even-sized “modern” and “ancient” samples from color populations are analyzed and the color pallett is balanced (Fig. 19A). In the more realistic scenario where the color pallet is imbalanced and sample sizes differ, PCA produced incorrect results where ancient Green (aGreen) clustered with modern Yellow (mYellow) away from its closest mGreen that clustered close to aRed. mPurple appeared as 4-ways mixed of aRed, aBlue, mCyan, and mDark Blue. Instead of being at the center (Fig. 19A), Black became an outgroup and its distances to the other colors were distorted (Fig. 19B). Projecting “ancient” colors onto “modern” ones also highly misrepresented the relationships among the ancient samples as aRed overlapped with aBlue or aGreen, mYellow appeared closer to mCyan or aRed, and the outgroups continuously changed (Fig. 19C,D). Note that the first two PCs of the last results explained most of the variance (89%) of all anlyses.

**Figure 20:**
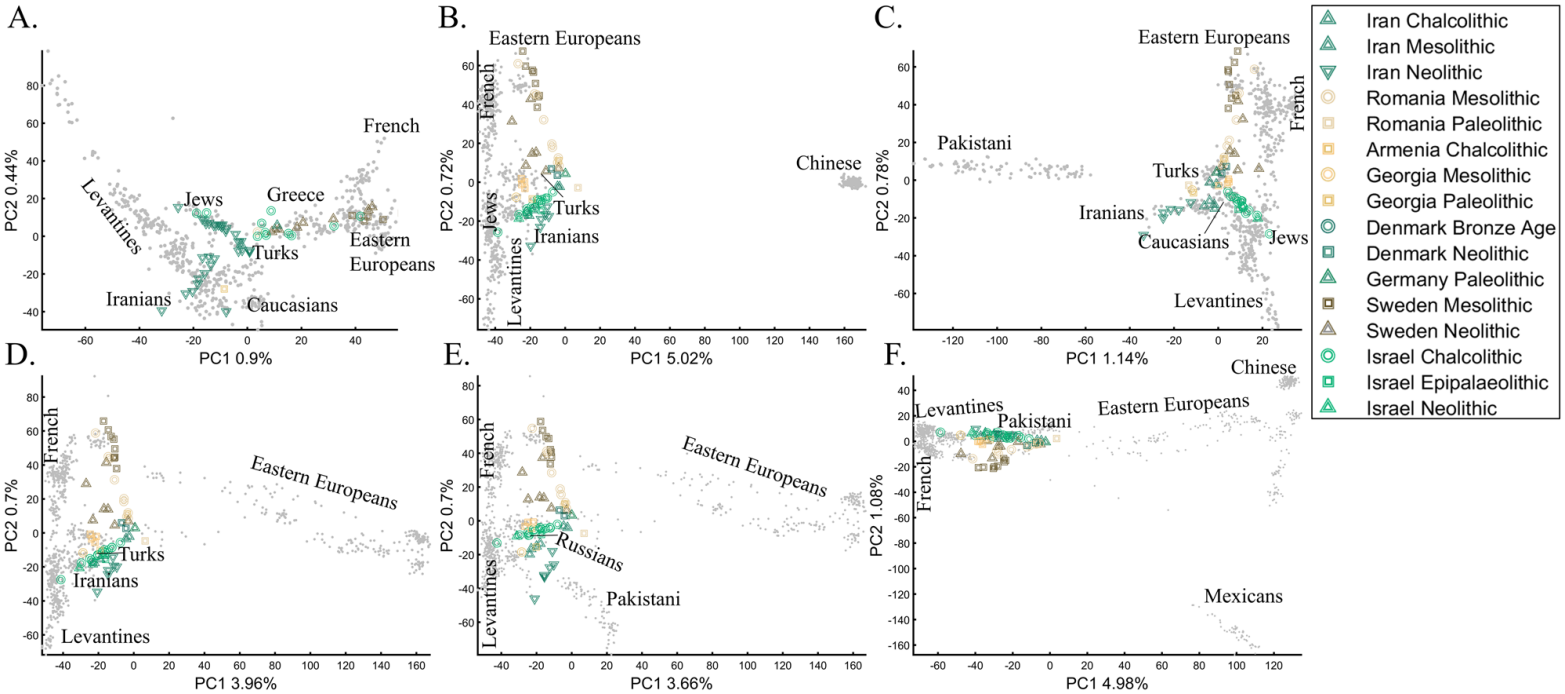
PCA of 65 ancient Palaeolithic, Mesolithic, Chalcolithic, and Neolithic from Iran (12), Israel (16), the Caucasus (7), Romania (10), Scandinavia (15), and Central Europe (5) (colorful shapes) projected onto modern-day populations of various sample sizes (grey dots, black labels). The full population labels are shown in Supplementary Fig. S8. In addition to the modern-day populations used in (**A**), the following subfigures also include (**B**) Han Chinese, (**C**) Pakistani (Punjabi), (**D**) additional Russians, (**E**) Pakistani (Punjabi) and additional Russians, and (**F**) Pakistani (Punjabi), additional Russians, Han Chinese, and Mexicans. The ancient samples remained the same in all the analyses. In each plot (**A**–**F**), the ancient Levantines cluster with different modern-day populations.

Recently, Lazaridis et al.^14^ projected ancient Eurasians onto modern-day Eurasians and reported that ancient samples from Israel clustered at one end of the Near Eastern “cline” and ancient Iranians at the other, close to modern-day Jews. Insights from the positions of the ancient populations were then used in their admixture modeling that supposedly confirmed the PCA results. To test whether the authors’ inferences were correct and to what extent those PCA results are unique, we used similar modern and ancient populations to replicate the results of Lazaridis et al.^14^ (Fig. 20A). By adding the modern-day populations that Lazaridis et al.^14^ omitted, we found that the ancient Levantines cluster with Turks (Fig. 20B), Caucasians (Fig. 20C), Iranians (Fig. 20D), Russians (Fig. 20E), and Pakistani (Fig. 20F) populations. The overlap between the ancient Levantines and other populations also varied widely, whereas they cluster with ancient Iranians and Anatolians, Caucasians, or alone, as a “population isolate.” Moreover, the remaining ancient populations exhibited conflicting results inconsistent with our understanding of their origins. Mesolithic and Neolithic Swedes, for instance, clustered with modern Eastern Europeans (Fig. 20A–C) or remotely from them (Fig. 20D–F). These examples show the wide variety of results and interpretations possible to generate with ancient populations projected onto modern ones. Lazaridis et al.’s^14^ results are neither the only possible ones nor do they explain the most variation. It is difficult to justify Lazaridis et al.’s^14^ preference for the first outcome where the first two components explained only 1.35% of the variation (in our replication analysis. Lazaridis et al. omitted the proportion of explained variation) (Fig. 20A), compared to all the alternative outcomes that explained a much larger portion of the variation (1.92–6.06%).

We note that for high dimensionality data where markers are in high LD, projected samples tend to “shrink,” i.e., move towards the center of the plot. Corrections to this phenomenon have been proposed in the literature, e.g., Ref.^63^. This phenomenon does not affect our datasets, which are very small (Fig. 19) or LD pruned (Fig. 20).

### The case of marker choice

**Figure 21:**
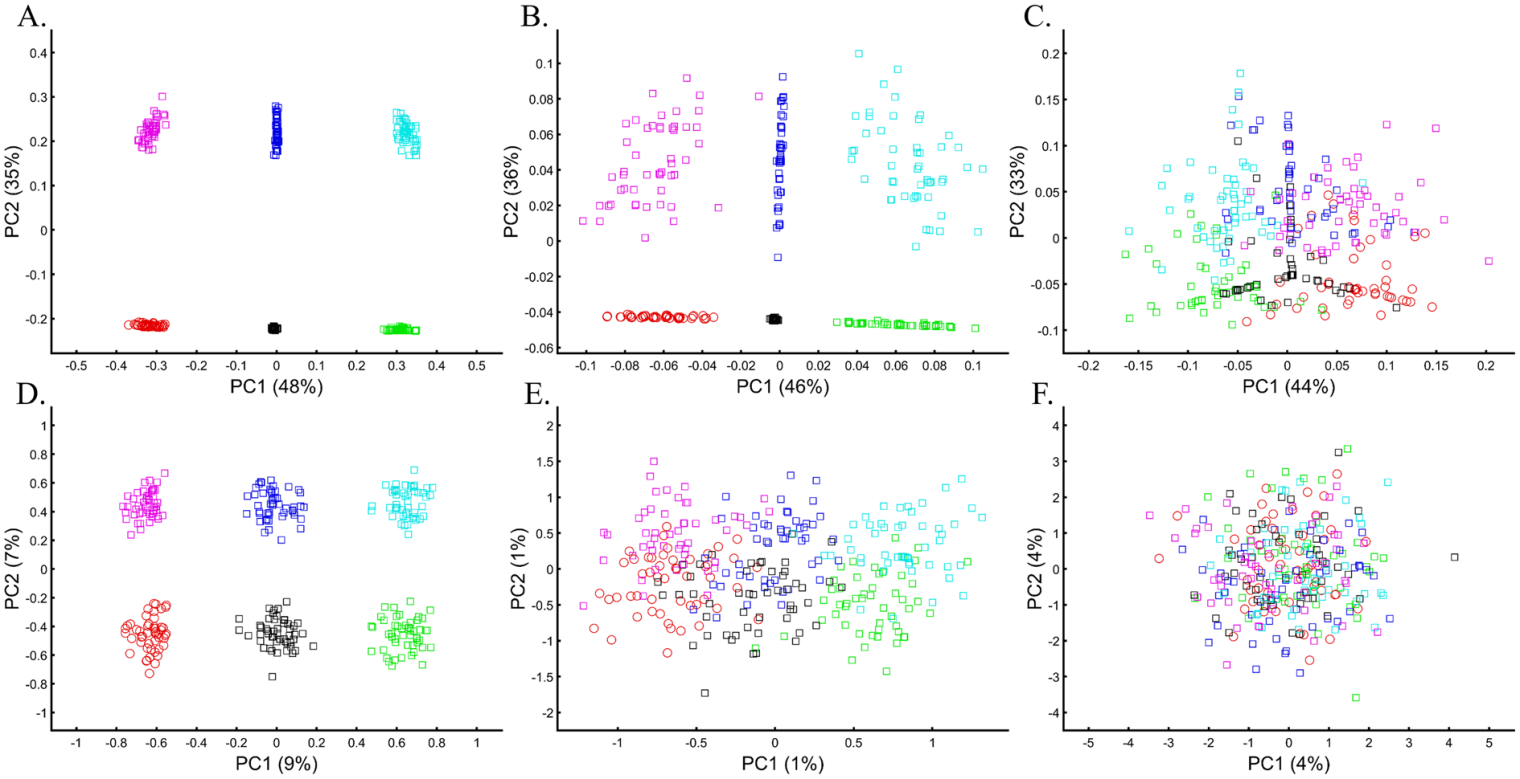
Testing the effects of missingness and noise in a PCA of six fixed-size (*n* = 50) samples from color populations. The top plots show the effect of missingness alone or combined with noise: (**A**) 50% missingness, (**B**) 90% missingness, and (**C**) 90% missingness and low-level random noise in all the markers. The bottom plots test the effect of noise when added to the original markers in the above plots using: (**D**) 30 random markers, (**E**) 300 random markers, and (**F**) 3000 random markers. Colors include Red [1,0,0], Green [0,1,0], Blue [0,0,1], Cyan [0,1,1], Yellow [1,1,0], and Black [0,0,0].

The effect of marker choice on PCA results received little attention in the literature. Although PCA is routinely applied to different SNP sets, the PCs are typically deemed comparable. In forensic applications, that typically employ 100–300 markers, this is a major problem. To evaluate the effect of various markers on PCA outcomes, it is unfeasible to use our color model, although it can be used to study the effects of missing data and noise, which are common in genomic datasets and reflect the biological properties of different marker types in capturing the population structure. Remarkably, the addition of 50% (Fig. 21A) and even 90% missingness (Fig. 21B) allowed recovering the original population structure. The structure decayed when random noise was added to the latter dataset (Fig. 21C). To further explore the effect of noise, we added random markers to the dataset. An addition of 10% of noisy markers increased the dataset's disparity, but it still retained the original structure (Fig. 21D). Interestingly, even adding 100% noisy markers allowed identifying the original structure's key features (Fig. 21E). Only when adding 1000%, noisy markers did the original structure disappear (Fig. 21F). Note that the introduction of noise has also sliced the percent of variation explained by the PCs. These results highlight the importance of using ancestry informative markers (AIMs) to uncover the true structure of the dataset and accounting for disruptive markers.

**Figure 22:**
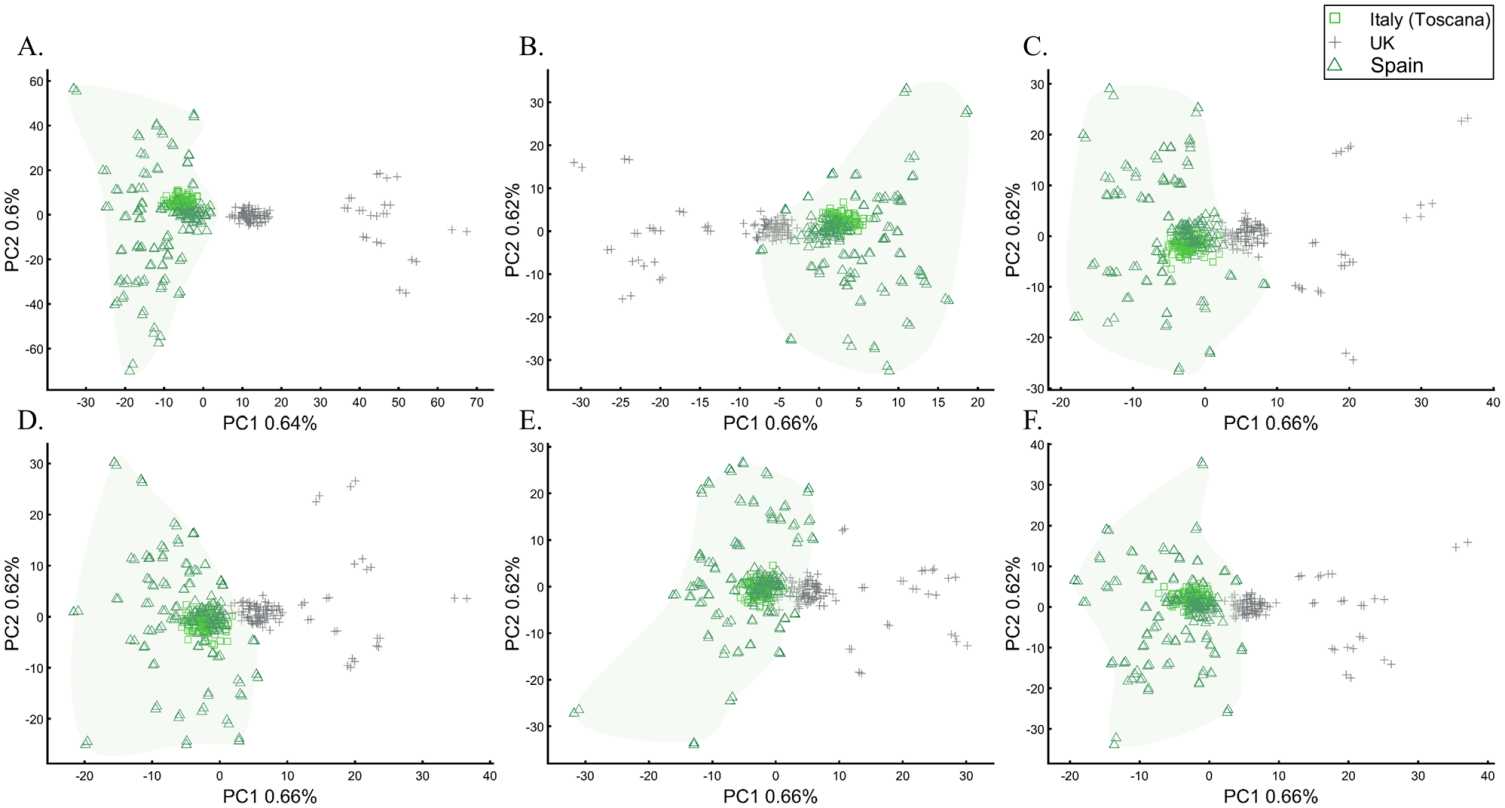
PCA of Tuscany Italians (*n* = 115), British (*n* = 105), and Iberians (*n* = 150) across all markers (*p* ~ 129,000) (**A**) and different marker types (*p* ~ 30,000): (**B**) coding SNPs, (**C**) intronic SNPs, (**D**) protein-coding SNPs, (**E**) RNA molecules, and (**F**) upstream and downstream SNPs. Convex hull was used to generate the European cluster.

To evaluate the extent to which marker types represent the population structure, we studied the relationships between UK British and other Europeans (Italians and Iberians) using different types of 30,000 SNPs, a number of similar magnitude to the number of SNPs analyzed by some groups^64,65^. According to the full SNP set, the British do not overlap with Europeans (Fig. 22A). However, coding SNPs show considerable overlap (Fig. 22B) compared with intronic SNPs (Fig. 22C). Protein coding SNPs, RNA molecules, and upstream or downstream SNPs (Fig. 22D–F, respectively) also show small overlap. The identification of “outliers,” already a subjective measure, may also differ based on the proportions of each marker type. These results not only illustrate how the choice of markers and populations profoundly affect PCA results but also the difficulties in recovering the population structure in exome datasets. Overall, different marker types represent the population structure differently.

### The case of inferring a personal ancestry

**Figure 23:**
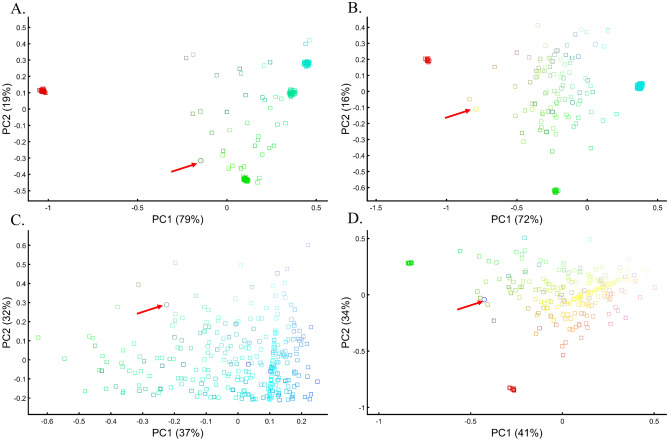
Inferring single individual ancestries using reference individuals. In (**A**) Using even-sized samples from reference populations (*n* = 37): Red [1,0,0], Green [0,1,0], bright Cyan [0, 0.9, 0.8], dark Cyan [0, 0.9, 0.6], heterogeneous darker Cyan [0, 0.9, 0.4] with high standard deviation (0.25) with a light Green test individual [0, 0.5, 0]. In (**B**) Using the same reference populations as in (**A**) with uneven-sizes: Red (*n* = 15), Green (*n* = 15), bright Cyan (*n* = 100), dark Cyan (*n* = 15), heterogeneous darker Cyan (*n* = 100), with a Yellow test indiviaul (1,1,0). In (**C**) A heterogeneous Cyan population [0, 1, 1] (*n* = 300) with high standard deviation (0.25) and a Grey test individual (0.5, 0.5, 0.5). In (**D**) Red [1,0,0] (*n* = 10), Green [0,1,0] (*n* = 10), a heterogeneous population [1, 1, 0.5] (*n* = 200) and a Blue test individual (0,0,1).

PCA is used to infer the ancestry of individuals for various purposes, however a minimal sample size of one, may be even more subjected to biases than in population studies. We found that such biases can occur when individuals with Green (Fig. 23A) and Yellow (Fig. 23B) ancestries clustered near admixed Cyan individuals and Orange, rather than with Greens or by themselves, respectively. One Grey individual clustered with Cyan (Fig. 23C) when it is the only available population, much like a Blue sample clustered with Green samples (Figs. 23D).

Arguably, one of the most famous cases of personal ancestral inference occurred during the 2020 US presidential primaries when a candidate published the outcome of their genetic test undertaken by Carlos Bustamante that tested their Native American ancestry (https://elizabethwarren.com/wp-content/uploads/2018/10/Bustamante_Report_2018.pdf). Analyzing 764,958 SNPs, Bustamante sought to test the existence of Native American ancestry using populations from the 1000 Genomes Project and Amerindians. RFMix^66^ was used to identify Native American ancestry segments and PCA, elevated to be a “machine learning technique,” to verify that ancestry independently of RFMix. The longest of five genetic segments, judged to be of Native American origin, was analyzed using PCA and reported to be “clearly distinct from segments of European ancestry” and “strongly associated with Native American ancestry” as it clustered with Native Americans distinctly from Europeans and Africans (Fig. 1 in their report) and between Native American samples (Fig. 2 in their report). Bustamante concluded that “While the vast majority of the individual’s ancestry is European, the results strongly support the existence of an unadmixed Native American ancestor in the individual’s pedigree, likely in the range of 6–10 generations ago”.

**Figure 24:**
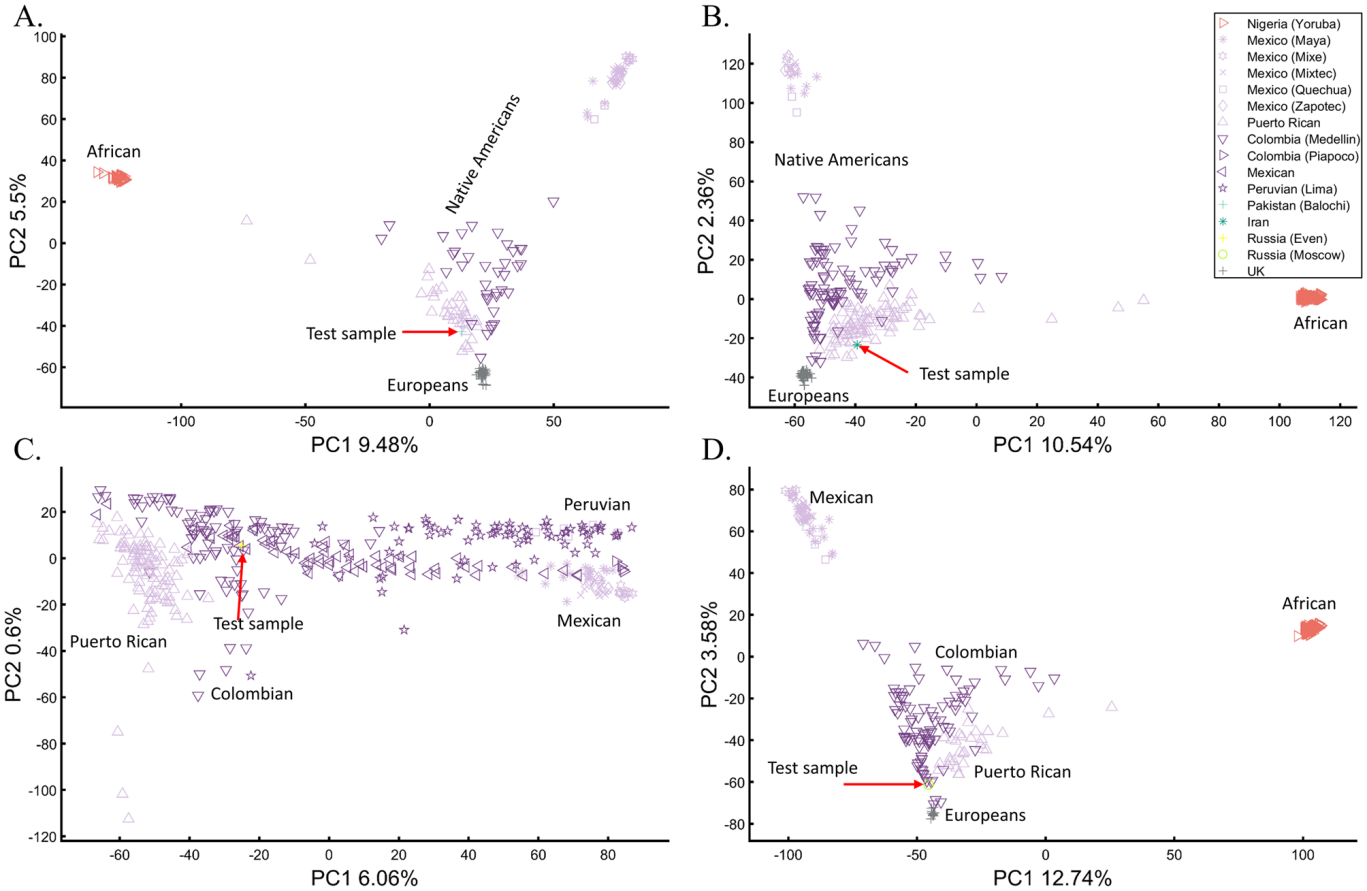
Evaluation of Native American ancestry for four Eurasians. (**A**) Using even-sample size (*n* = 37) for Africans, Mexican-Americans, British, Puerto Ricans, Colombians, and a Pakistani. (**B**) Using uneven-sample sizes, for Africans (*n* = 100), Mexican-Americans (*n* = 20), British (*n* = 50), Puerto Ricans (*n* = 89), Colombians (*n* = 89), and an Iranian. (**C**) Analyzing a whole-Amerindian cohort of Colombian (*n* = 93), Mexican-Americans (*n* = 117), Peruvian (*n* = 75), Puerto Ricans (*n* = 102), and an Even Russian. (**D**) Using uneven-sample sizes, for Africans (*n* = 100), Mexican-Americans (*n* = 53), British (*n* = 20), Puerto Ricans (*n* = 30), Colombians (*n* = 89), and a Moscow Russian. All the samples were randomly selected.

We have already shown that AJs (Fig. 9C) and Pakistanis (Fig. 14D) can cluster with Native Americans. With the candidate’s DNA unavailable (and their specific European ancestry undisclosed), we tested whether the two PCA patterns observed by Bustamante can be reproduced for modern-day Eurasians without any reported Native American ancestry (Pakistani, Iranian, Even Russian, and Moscow Russian) (Figs. 24A–D, respectively).

These analyses show that the experimenter can easily generate desired patterns to support personal ancestral claims, making PCA an unreliable and misleading tool to infer personal ancestry. We further question the accuracy of Bustamante’s report, provided the biased reference population panel used by RFMix to infer the DNA segments with the alleged Amerindian origin, which excluded East European and North Eurasian populations. We draw no conclusions about the candidate’s ancestry.

## Discussion

The reproducibility crisis in science called for a rigorous evaluation of scientific tools and methods. Due to PCA’s centrality in population genetics, and since it was never proven to yield correct results, we sought to assess its reliability, robustness, and reproducibility for twelve test cases using a simple color-based model where the true population structure was known and real human populations. PCA failed in all three measures.

PCA did not produce correct and\or consistent results across all the design schemes, whether even-sampling was used or not, and whether for unmixed or admixed populations. We have shown that the distances between the samples are biased and can be easily manipulated to create the illusion of closely or distantly related populations. Whereas the clustering of populations between other populations in the scatter plot has been regarded as “decisive proof” or “very strong evidence” of their admixture^18^, we demonstrated that such patterns are artifacts of the sampling scheme and meaningless for any bio historical purposes. Sample clustering, a subject that received much attention in the literature, e.g., Ref.^9^, is another artifact of the sampling scheme and likewise biologically meaningless (e.g., Figs. 12, 13, 14, 15), which is unsurprising if the distances are distorted. PCA violations of the true distances and clusters between samples limit its usability as a dimensional reduction tool for genetic analyses. Excepting PC1, where the distribution patterns may (e.g., Fig. 5a) or may not (e.g., Fig. 9) bear some geographical resemblance, most of the other PCs are mirages (e.g., Fig. 16). The axes of variation may also change unexpectedly when a few samples are added, altering the interpretation.

Specifically, in analyzing real populations, we showed that PCA could be used to generate contradictory results and lead to absurd conclusions (*reductio ad absurdum*), that “correct” conclusions cannot be derived without *a prior*i knowledge and that *cherry-picking* or *circular reasoning* are always needed to interpret PCA results. This means that the difference between the a posteriori knowledge obtained from PCA and a priori knowledge rests solely on belief. The conflicting PCA outcomes shown here via over 200 figures demonstrate the high experimenter’s control over PCA’s outcome. By manipulating the choice of populations, sample sizes, and markers, experimenters can create multiple conflicting scenarios with real or imaginary historical interpretations, *cherry-pick* the one they like, and adopt *circular reasoning* to argue that PCA results support their explanation.

Overall, the notion that PCA can yield biologically or historically meaningful results is a misconception supported by a priori knowledge and post hoc reasoning. PCA “correct” results using some study designs are utterly indistinguishable from incorrect results constructed using other study designs, and neither design could be justified a priori to be the correct one. Likewise, PCA correctly represented the genetic distances and clusters for a  miniscule fraction of the samples (e.g., Fig. 6) who were otherwise indistinguishable from the remaining samples whose genetic distances were distorted. Therefore, like a broken clock, PCA *can* be tuned by the experimenter (e.g., Fig. 20) to yield presumed “correct” results, and “correct” results can be *cherry-picked* if known a priori*,* but neither is evidence to the accuracy of PCA. Just like a broken clock, working clocks (i.e., other tools) are essential to decide on the “correct” PCA results. This begs the question of why use PCA at all, particularly as a first hypothees generator.

Some authors^67^ revealed the cards by proposing to use PCA for “exploration” purposes; however, the “exploration” protocol was never scripted, and neither was the method by which a posteriori knowledge can be garnered from this journey to the unknown. “Exploration” is thereby synonymous with *cherry-picking* specific PCA results deemed similar to those generated by other tools. If this was a realistic approach, the practice of PCA could have been simply dismissed as cumbersome and unnecessary. However, in the literature, the reverse procedure is dominant, i.e., the broken clock is used to call the hours for the other clocks. We believe that such design is popular because downstream analyses are equally manuverable or designed to address specific questions, allowing the experimenter a control over the general narrative.

Indeed, after “exploring” 200 figures generated in this study, we obtained no a posteriori wisdom about the population structure of colors or human populations. We showed that the inferences that followed the standard interpretation in the literature were wrong. PCA is highly subjected to minor alterations in the allele frequencies (Fig. 12), study design (e.g., Fig. 9), or choice of markers (Fig. 22) (see also Refs.^57,68^). PCA results also cannot be reproduced (e.g., Fig. 13) unless an identical dataset is used, which defeats the usefulness of this tool. In that, our findings thereby join similar reports on PCA’s unexpected and arbitrary behavior^69,70^. Note that variations in the implementations of PCA (e.g., PCA, singular value decomposition [SVD], and recursive PCA), as well as various flags, as implemented in EIGENSOFT, yield major differences in the results—none more biologically correct than the other. That the same mathematical procedure produces biologically conflicting and false results proves that bio historical inferences drawn only from PCA are fictitious.

Several aspects of this study are important to emphasize. First, this study does not ask whether the PC transformation is correct. If properly implemented, the computational procedure that computes the principal components and uses them to change the basis of the data is considered correct. This study asks whether the PC transformation produces *correct* or *wrong* outcomes for the original datasets, consisting of colors populations, where the truth is known. For real populations, we avoided judging results to be correct or not since many of those questions are subjects of ongoing debates. Instead, we asked whether PCA results are consistent with each another, align with their interpretation in the literature, and can lead to absurd conclusions. Second, this study focuses on genetic variation data, particularly human data, that have particular characteristics. For other data types or datasets not tested here, PC analyses may be more successful, e.g., Ref.^71^, if they survive the test criteria presented here. We note, however, that PCA produced incorrect results in our simple model (e.g., Fig. 3) and that criticism is neither rare nor unique to genetics (see criticism of PCA in geology^72^ and physical anthropology^73^). To better understand how PCA reached prominence, we shall review the historical debate on whether the PCA trnasformation represents the genetic data correctly.

### A brief history of PCA and its application to population genetics

It is well-recognized that Pearson^74^ introduced PCA and Hotelling^75^ the terminology. Hotelling’s motivation was to address the problem of evaluating independent mental traits in psychology. Thurstone presented another principal axes solution to the problem of factor analysis^75^. However, he later reconciled, as he could not see how they describe a meaningful psychological model^76^. The argument about the truthfulness and reliability of PCs continues to this day^77^.

In population genetics, PCA is primarily used to reduce the dimensionality of multivariate datasets by linearly transforming the genotypes into a set of mutually uncorrelated principal components (PCs) ranked according to their variances. As most of the original variability is contained in the primary two PCs, they are typically visualized on a colorful scatter plot. The early work of Cavalli-Sforza suggested that PCA can detect ancient migrations and population spreads^78,79^ in the genomic data. The authors proposed that PCA will “give us new insight into the evolutionary history of the populations represented in the map”^78^ although later they explained their inability to interpret the PCA results for Africans because “the genetic and archeological knowledge in these regions is not as detailed as in Europe”^79^, i.e., in the lack of a priori knowledge. Cavalli-Sforza’s arguments were not very convincing.

During the twentieth century, PCA was sparsely employed in genomic analyses alongside other multidimensional scaling tools. The next-generation sequencing revolution in the early twenty-first century produced large genomic datasets that required new and powerful computational tools with appealing graphical interfaces, like STRUCTURE^80^. PCA was not used in the publications of the first two HapMaps nor the HGDP dataset^81–83^.

In 2006, Price et al.^10^ introduced the SmartPCA tool (EIGENSOFT package) and claimed that PCA has “a solid statistical footing” that can “discover structure in genetic data” even in admixed populations. Those claims were made based on a simulated dataset and an application of PCA to a dataset of European Americans, which revealed an incoherent pattern claimed to reflect genetic variation between northwest and southeast Europe. Simultaneously, Patterson et al.^9^ applied PCA to three African and three Asian populations claiming that the dispersion patterns of the primary two PCs reflect the true population structure. SmartPCA offered no remedy to the known problems with PCA, only new promises.

The next milestone in the rise of PCA to prominence was the work of Novembre and colleagues^32^ that showed a correlation between PCA and geography among Europeans. The authors applied PCA to a dataset of European genotypes, positioned the PCs on Europe’s map, and rotated their axes to increase the correlation with Europe’s map. After fitting a model of longitude and latitude that included PC1, PC2, and their interactions, samples were positioned on Europe’s map. The authors claimed that “the resulting figure bears a notable resemblance to a geographic map of Europe” and reported that, on average, 50% of samples from populations with greater than six samples were predicted within less than 400 km of their country. Most of those populations, however, were from the extreme ends of the map (Italy, UK, and Spain) and were predicted most accurately because PCA maximizes the variance along the two axes. By contrast, samples from mid and north-Europe were predicted most poorly. Overall, the authors’ approach classified about 50% of the samples in the final dataset to within 400 km of their countries. Only 24% of the samples from all European countries (Table 3 in Ref.^32^) were predicted to their correct country, 50% of the populations were predicted within 574 km (about the distance from Berlin to Warsaw), and 90% of the populations were predicted within 809 km (about the distance from Berlin to Zurich). Overall, it is fair to say that in practice, this method does not perform as implied because it strongly depends on the specific cohort. Therefore, it does not have any practical applications. A more proper title for the paper would have been “populations can be selected to mirror geography in a quarter of Europe”. Novembre et al.'s study was iconic, which in retrospect may be unwarranted, since authors always claimed to see geographical patterns in PCA results irrespective to Novembre et al.'s transformation. Later, Yang et al.^84^ claimed to have expanded the method to global samples. Elhaik et al.^85^ showed that the new method has less than 2% accuracy, with some samples being predicted outside our planet. Thus far, no PCA or PCA-like application has ever reached an accuracy higher than 2% worldwide^86^. By contrast, an admixture-based approach achieved 83% accuracy in classifying individuals to countries and even islands and villages^85^.

Ignoring these methodological problems and further promoting their PCA tool, Reich et al.^44^ wrote in an editorial for the Novembre et al.'s study that “PCA has a population genetics interpretation and can be used to identify differences in ancestry among populations and samples, regardless of the historical patterns underlying the structure,” that “PCA is also useful as a method to address the problem of population stratification—allele frequency differences between cases and controls due to ancestry differences—that can cause spurious associations in disease association studies,” and finally that “PCA methods can provide evidence of important migration events”—none of which were supported by the work of Novembre et al.

**Figure 25:**
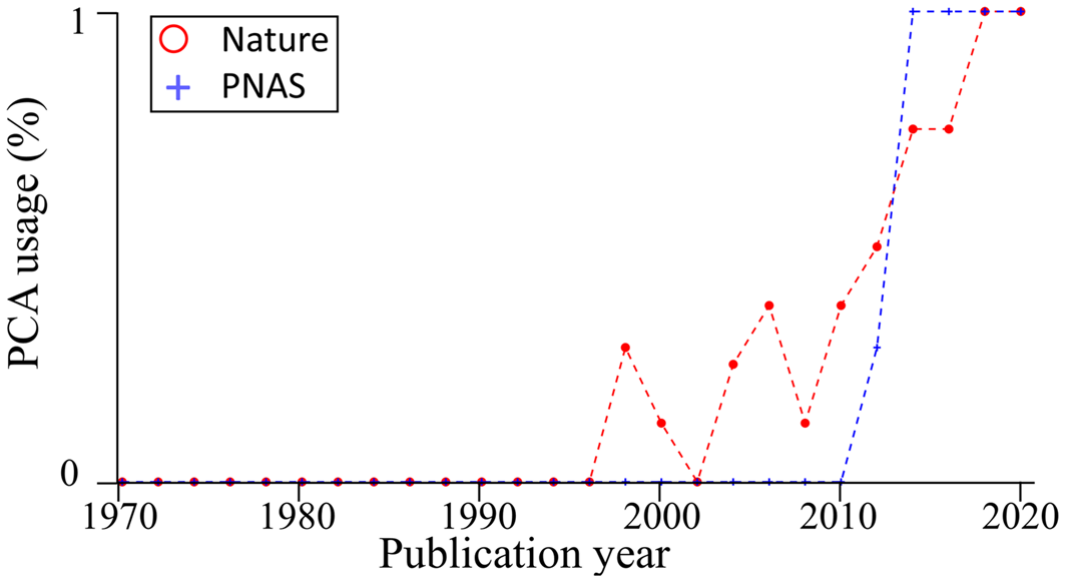
Evaluating the usability of a PCA in population genetic publications by sampling four random population genetic papers per year from Nature and PNAS. The percent of publications that used at least one PCA is shown.

After its applications to the HGDP^87^ and HapMap 3^88^ datasets, PCA became the foremost utility in population genetic investigations, reaching ”fixation” by 2013, the point where it is used almost in every paper in the field (Fig. 25).

### Evaluating the core properties of PCA

Table 1 summarizes the main findings of the twelve test cases analyzed here. Several additional limitations of PCA are worth highlighting since they may not have been evident in the test cases. First, PCA typically explains a tiny part of the variation (Supplementary Fig. S9) that may have a genealogical interpretation^69^, but not only does it grow smaller as more samples are added (Supplementary Fig. S9), it also grows in inaccuracy (Fig. 9). This leads to a paradox, whereas increasing the sample size, which intuitively should be expected to increase the accuracy of analyses, decreases the proportion of explained variance and accuracy. Second, analyzing only the top two PCs does not solve the rapid decline in the proportion of explained variation (Supplementary Fig. S10). Interestingly, the average variance explained by the two primary PCs over hundreds and thousands of individuals from different populations is very small (Supplementary Fig. S10, inset). Third, PCs higher than three not only explain a minuscule amount of variation, but they also cannot differentiate the true data structure from noise (Supplementary Fig. S11). In other words, PC plots where the first two PCs explain ~ 1% of the variance, as we calculated for Lazaridis et al.^14^, capture as much of the population structure as they would from a randomized dataset. Recall that all the datasets analyzed here include AIMs that improve the discovery of population structure. The fourth limitation concerning PCA’s characteristic is the “big-p, little-n,” where *p* stands for dimensions and *n* for samples, otherwise known as the *p* >  > *n* problem or *the curse of dimensionality*^89^. Briefly, it refers to the phenomenon that arises when analyzing data in high-dimensional spaces unobserved in lower-dimensional spaces. As a dimensionality reduction technique, PCA aims to address this problem. However, PCA introduces biases of its own. PCA misrepresents the distances and clusters. In high-dimensional space, the distances between the data points increase compared to low-dimensional space (Supplementary Fig. S12). As such, formerly close population samples appear more distanced and no longer cluster. In other words, cases and controls cannot be reliably identified in high-dimensional data, as is commonly done. Finally, PCA adjustments may be disadvantageous. We show that applying PCA adjustment to case–control data yielded a higher proportion of false positives, a smaller proportion of true positives, and weaker *p*-values (Supplementary Text 3).

**Table 1:** A summary of the main findings of the twelve test cases studied here.

Test case	Main findings
The near-perfect case of dimensionality reduction	The observed distances in a PC plot do not reflect the distances between the samples Even sample size changes do not affect the topography of the outcomes for same-size populations
Different sample sizes	Changing sample sizes creates alternative results A priori knowledge is vital to interpreting PCA results. Without it, interpreting PCA plots leads to nonsensical conclusions
One admixed population	The proportion of explained variance by the PCs is not biologically meaningful and is not a measure of PCA accuracy Clines like the “Ancestral North Indians” (ANI) and “Ancestral South Indians” (ASI) are artifacts of the PCA scheme PCA results do not reflect genetic or biological distances Admixture levels and direction cannot be inferred from PCA PCA schemes can be manipulated to support ethnocentric claims, as with the case of Ashkenazic Jews (AJs) Experimenters can use PCA to produce near-endless conflicting and absurd historical scenarios, all mathematically correct but biologically incorrect
Two or three-way admixed population (Supplementary Text 2.1)	PCA outcomes may appear, in part, meaningful based on a priori knowledge but are biologically meaningless and contradictory otherwise
Multiple admixed population	Alternating reference populations creates alternative results Including multiple admixed populations does not improve PCA accuracy PCA schemes can be manipulated to support origin or genetic distinctiveness claims
The case of multiple admixed populations without “unmixed” populations	Including multiple admixed populations without “unmixed” ones does not improve PCA accuracy Although a deterministic process, PCA behaves unexpectedly as minor variations can lead to an ensemble of different outputs that appear stochastic. Consequently, PCA results are irreproducible
Pairwise comparisons (Supplementary Text 2.2)	PCA can lead to erroneous conclusions concerning clustering, identity, and distance cross-dimensionally PCA clustering and distances are unpredictable and unreliable for studying the relationships between populations
Case–control matching and GWAS	Analyzing reference populations with mismatched ancestries respective to the unknown samples biases the ancestry inference of the latter PCA exhibits a high error rate when used to create genetically homogeneous clusters Analyzing higher PCs decreases the size of the homogeneous clusters and increases the size of the non-homogeneous ones Studying genetic association in a case–control setting, PCA adjusted results had more false positives, fewer true positives, and weaker *p*-values than unadjusted results “Exploring” PC plots yields no insight. The sole purpose of “exploration” is to allow experimenters to select their favorable solution based on their a priori knowledge
Projections	PCA projections are unreliable and misleading, with correct outcomes indistinguishable from incorrect ones
Ancient DNA	Projecting ancient populations onto modern ones allows the experimenter to choose favorable results Authors typically omit the amount of variance explained by the primary PCs because it is minuscule
Marker choice	Analyzing different markers creates alternative results Ancestry informative markers (AIMs) are more robust to noise and errors when studying the population structure
Inferring a personal ancestry	Using PCA to infer individual ancestry is unreliable and misleading Using PCA, experimenters can easily generate desired patterns to support personal ancestral claims

### Misuses of PCA in the literature

To understand how and why a tool with so many limitations became the foremost tool in population genetics, we will briefly review how authors handled those limitations.

We have already demonstrated that authors misinterpret PCA findings and do not disclose the amount of variation explained by PCA. Fascinatinglyedly, in 2008 Reich and colleagues found it necessary to assess “whether the proportion of the variance explained by the first PC is sufficiently large,” most likely before they realized just how small this variation really is. To the best of our knowledge, they omitted this information in their numerous publications that employed PCA, e.g., Refs.^14,45,62,90–93^.

Remarkably, Novembre and Stephens^94^ warned that “PCA results depend on the details of a particular dataset, they are affected by factors in addition to population structure, including distribution of sampling locations and amounts of data. Both these features limit the utility of PCA for drawing inferences about underlying processes” but nonetheless found PCA to be “undoubtedly an extremely useful tool for investigating and summarizing population structure,” and correctly anticipated that it will play “a prominent role in analyses of ongoing studies of population genetic variation”.

Although authors were aware that PCA results depended on the sample cohort, they continued using it, presenting only the results that fit their a priori hypotheses. For example, Tian et al.^49^ recognized that PCA “is sensitive to differences in the inclusion or exclusion of specific population groups” and that it “can be dramatically affected by differences in relatively small genomic regions that may not reflect true population substructure”. Likewise, Tian et al.^50^ noted that Ashkenazic Jews (AJs) “have a unique genotypic pattern that may not reflect geographic origins” and that “the inclusion or exclusion of particular ethnic groups… shifted the relationships in PCA”. They acknowledged that their findings “show that PCA results are highly dependent on which population groups are included in the analysis”. Still, both groups drew conclusions based on PCA and their a priori perceptions. Price et al.^95^ needed no Leavnatine populations to conclude from a PCA plot with Ashkenazic Jews and Europeans that “both Ashkenazi Jewish and southeast European ancestries are derived from migrations/expansions from the Middle East and subsequent admixture with existing European populations”. Provided its flexibility, it should come as no surprise that PCA and, in one case, Multi-dimensional scaling (MDS)^96^ spearheaded claims of Levantine origin for AJs^97^. We showed that PCA could be easily engineered to foster American, Iberian, West, Central, and South European, Britain, Scandinavian, South Central Asian, Central Asian, Middle Eastern, Caucasian, and even Levantine origins for AJs.

PCA applications in biology have been criticized by several groups. McVean^69^ cautioned that “Sub-sampling from populations to achieve equal representation, as in Novembre et al.^32^, is the only way to avoid this problem [= the distortion of the projection space]” and that “the influence of uneven sample size can be to bias the projection of samples on the first few PCs in unexpected ways”. However, these statements are incorrect. First, Novembre et al.’s sample sizes ranged from 1 to 219. Second, McVean’s simulation was limited to the case of symmetric populations arranged in a lattice formation, as in Figs. 1C or 19A. This led McVean to believe that accuracy can be achieved when sample sizes are even and thereby have some merit (“The result provides a framework for interpreting PCA projections in terms of underlying processes, including migration, geographical isolation, and admixture”). Had McVean explored the slightly more realistic case of populations sampled evenly with uneven contributions to the covariance matrix (e.g., Figs. 4A, 9A), he would have realized that PCA’s accuracy is extremely limited to well-controlled simulations of even-sized samples from isotropic populations (symmetrically distributed across all the dimensions). In reality, “populations” are unknown, are of uneven population sizes, are anisotropic, and sampled unevenly using different markers. These limitations invalidate PCA as a useful tool for population genomic studies. Elhaik and Ryan^57^ showed that PCA could not model admixed samples, resonating our findings using forward simulation (Supplementary Text 1). Elhaik et al.^85^ showed that PCA-like tools could not be used for biogeography, which is not surprising if PC distances are meaningless. François et al.^67^ noticed that the gradients observed in the first PC often contradict formulated expectations and offered a biological explanation for the phenomenon. They concluded that PCA should be considered as a data exploration tool (i.e., *cherry-picking*) and that interpreting the results in terms of past routes of migration “remains a complicated exercise”. Björklund^98^ raised concerns about sampling problems that render PCA biologically meaningless and provided several recommendations, like evaluating the distinctness of the PC’s and presenting the percent of explained variance. The practice of ignoring sample dates in paleogenomic analyses that incorporates ancient and modern samples has also been criticized^99^. Recently, Chari et al.^70^ showed that in single-cell gene expression analyses, where PCA pre-conditioned *t*-SNE and UMAP visuals are often used to infer or confirm relationships between cells in qualitative and quantitative manners for many purposes, including to “validate” clustering, PCA caused major distortion of the data and when analyzing equidistant points was tantamount to applying a random projection. The authors developed an art model and showed that it produces comparable metrics to those produced by the PCA-refined dataset on which *t*-SNE and UMAP were applied. The authors reported that the “application of PCA to a set of equidistant points produces an arbitrary projection that will depend on software implementation details, including random number seeds and the numerical methods implemented for computing eigenvalues and eigenvectors”. Our findings, albeit in population genetics, demonstrate that with the exceptions discussed above, all PCA results are wrong and are independent of the level of “cautiousness” exhibited by the experimenter even for “exploration” purposes.

### PCA as a *Dataism* exercise in population genetics

*Dataism* describes an ideology formed by the emergence of Big Data, where measuring the data is the ultimate achievement^100^. Dataism proponents believe that with sufficient data and computing power, the world’s mysteries would reveal themselves. Dataism enthusiasts rarely ask themselves *if PCA results are correct* but rather *how to interpret the results correctly*. As such, clustering is interpreted as identity, due to *common ancestry* and its absence as *genetic drift*. Populations nested between other populations are *admixed* or *isolates,* and those at the corners of the PC scatter plot are *unmixed, pure,* or *races.*

Although a newly coined term, the roots of the dataism philosophy are traceable to the Hotelling-Thurstone debate and specifically to the Cavalli-Sforza-Sokal conundrum. Cavalli-Sforza et al.^101^ (p338) explained the first six components in ancient human cross-continental expansions, but they never explained to what extent those historical inferences were distinguished from the null hypothesis since they did not have any. Sokal and colleagues showed that the PCA maps are subject to substantial errors and that apparent geographic trends may be detected in spatially random data (the null). Sokal et al. did not express doubt in human history, only that it reveals itself in the PC maps, as do we. Cavalli-Sforza’s group responded that Sokal et al.’s sampling scheme was extremely irregular^102^ and questioned Sokal et al.’s disbelief in a wrong method that yields a conclusion that they were willing to accept otherwise. Sokal et al.^103^ were concerned with the lack of response to their original inquiries, the PC’s interpolation (to overcome gaps in the data) and smoothing technique that introduced more noise, the specific sampling scheme of Cavalli-Sforza and colleagues that appeared incidental rather than genuinely comprehensive, and the continued absence of a null model. In further criticism of Cavalli-Sforza et al.^101^, they claimed that whereas some of the results appear biologically sound, others are not, yet both are discussed equally seriously. Cavalli-Sforza^104^ stuck by PCA and the historical inferences (The Neolithic spread to Europe made “between 8000 and 5000 years ago”) that can be allegedly derived from it. In other words, whereas Cavalli-Sforza and colleagues believed that once sufficient data are available, the value of PCA for bio-history would reveal itself, Sokal and colleagues questioned the robustness and reliability of the approach to generate valid historical and ethnobiological results and cautioned that data that “have been interpolated or smoothed, invite ethnohistorical interpretation by the unwary”^105^. The issues at the heart of the debate were not as much about biostatistics as about dataism.

At first, Sokal and colleagues had the upper hand in the debate. PCA was not used in the first Big Data analyses of 2003–2005 until resurrected by Price et al.^10^. Price et al. ignored Sokal’s reasoning. They produced no null model nor proved that the method yields biologically correct results. The appeal of their tool was mainly its applicability to the large genetic datasets that had begun emerging at that time and the visual appeal of PC scatterplots that condensed these data. Interestingly, Novembre and Stephens^94^ showed that the PCA structured patterns that Cavalli-Sforza and others have interpreted as migration events are no more than mathematical artifacts that arise when PCA is applied to standard spatial data in which the similarity between locations decays with geographic distance. Nonetheless, their warning was largely ignored, perhaps because the parallel study of Novembre et al.^32^ left a stronger impact, and Cavalli-Sforza’s dataism was vindicated.

Evidently, PCA produces patterns no more historical than Alice in Wonderland and bear no more similarity to geographical maps. Overall, the positioning of a method that lacks any measurable power, a test of significance, or a null model, which any diligent scientist should seek at the forefront of population genetic analyses, is problematic at the very least. It would not be an exaggeration to consider PCA the Rorschach of population genetics, a procedure that is almost entirely open to manipulations and consequent interpretations, where researchers may see “geographical maps” or “Neolithic clines” as they will. In PCA-driven science, almost all the answers are equally acceptable, and the truth is in the eyes of the beholder.

### Moving beyond PCA

As an alternative to PCA, we briefly note the advantages of a supervised machine-like model implemented in tools like the Geographic Population Structure (GPS)^85^ and Pairwise Matcher (PaM)^57^. In this model, gene pools are simulated from a collection of geographically localized populations. The ancestry of the tested individuals is next estimated in relation to these gene pools. In this model, all individuals are represented as the proportion of gene pools. Their results do not change when samples are added or removed in the second part of the analysis. Population groups are bounded within the gene pools, and inclusion in these groups can be evaluated. This model was shown to be reliable, replicable, and accurate for many of the applications discussed here, including biogeography^85^, population structure modeling^106^, ancestry inference^107^, paleogenomic modeling^108^, forensics^86^, and cohort matching^57^. An evaluation of other tools that may be useful to infer the population structure and their limitations can be found elsewhere^37,109^.

## Conclusions

PCA is a mathematical transformation that reduces the dimensionality of the data to a smaller set of uncorrelated dimensions called principal components (PCs), which has numerous applications in science. In population genetics alone, PCA usage is ubiquitous, with dozen standard applications. PCA is typically the first and primary analysis, and its outcomes determine the study design. That PCA is completely non-parametric is the source of its strength. Any genotype dataset can be rapidly processed with no concerns about parameters or data validity. It is also a weakness because the answer is unique and depends on the particular dataset, which is when reliability, robustness, and reproducibility become a concern. The implicit expectation employed by PCA users is that the variance explained along the first two PCs provides a reasonable representation of the complete dataset. When this variance is minuscule (as often with human populations), it poorly represents the data. Rather than consider using alternative analyses, authors often choose not to report the variation explained by PCA. Regardless, it is not a proxy for the reliability of the results.

Here, we carried out extensive analyses on twelve PCA applicaitons, using model- and real-populations to evaluate the reliability, robustness, and reproducibility of PCA. We found that PCA failed in all criteria and showed how easily it could generate erroneous, contradictory, and absurd results. This is not surprising because PCA is blind to the data and their meaning. The covariance matrix is calculated from the centered matrix itself created simply by subtracting the mean *A*_*u*_ from the original matrix A, disregarding the weights and geography. The remaining transformation consists of the dimensionality reduction, which is less problematic; however, that the first two PCs that capture most, but still a very small part of the genetic variation, are typically analyzed creates further misinterpretations. Given the omnipresence of PCA in science, an intriguing question is whether multidisciplinary PCA results should be reevaluated? Based on our analyses and critical evaluations published elsewhere, we cannot dismiss this possibility.

As PCA lacks any measurable significance or accuracy, we argue that its dominance in population genetics could not have been achieved without the adoption of two fallacies: *cherry-picking* or *circular reasoning* (i.e., “exploration”), the screening and selecting PCA scatterplots that fit preconceived hypotheses while ignoring the other plots, and the a priori where PCA results are interpreted based on pre-existing knowledge because PCA scatterplots are uninformative a posteriori. As a “black box” basking in bioinformatic glory free from any enforceable proper usage rules, PCA misappropriations, demonstrated here for the first time, are nearly impossible to spot.

The fact that population affinities vary appreciably between closely related, ostensively equivalent datasets is deeply worrying (PCA applications were cited 32,000-216,000 times). Researchers from adjacent fields like animal and plant or medical genetics may be even less aware of the inherent biases in PCA and the variety of nonsensical results that it can generate. We consider PCA scatterplots analogous to Rorschach plots. We find PCA unsuitable for population genetic investigations and recommend reevaluating all PCA-based studies.

## Methods

### Generating the color populations

All the color populations were generated in a similar way with the number of dimensions *p* equals 3. Every individual color was represented by [*P*_1_*R*N, P_2_*R*N, P_3_*R*N], where *P*_*1–3*_ are the three color dimensions or components that range from 0 to 1, *R* is pseudorandom value drawn from the standard normal distribution (Matlab’s function *randn*). *N* is noise set to 0.01 in almost all analyses, with the following exceptions where a larger noise was needed in Figs. 17 (*N* = 0.02), 19 (*N* = 0.02 or 0.05), 13B, 13C (*N* = 0.05), 13A (*N* = 0.17), and Supplementary Fig. S2.3C (*N* = 0.015). Colors are represented by a name and value (i.e., Red is [1,0,0] to which *R* and *N* were added), rounded up for brevity.

### Sample collection

Alongside the simulated color datasets, we employed three human genotype datasets:

1. 2068 global modern-DNA samples genotyped over 621,799 SNPs^14^ available at https://reich.hms.harvard.edu/sites/reich.hms.harvard.edu/files/inline-files/NearEastPublic.tar.gz,
2. the overlap of dataset 1, 2504 humans from the 1000 genome project^110^ available at ftp://ftp-trace.ncbi.nih.gov/1000genomes/ftp, and 471 Ashkenazic Jews^48^ available at http://www.ncbi.nlm.nih.gov/geo/query/acc.cgi?acc=GSE23636 (overall 5,043 samples) and
3. the overlap of dataset 2 and 514 ancient DNA samples from Allen Ancient DNA Resource (AADR) (version 44.3)^14^ (Supplementary Table S1) (overall, 5,557 samples).
  We used Lazaridis et al.’s^14^ dataset to LD-prune all the datasets. After LD pruning using PLINK command (50 10 0.8) and removing SNPs with missingness, allowing no more than five missing SNPs per sample, the datasets included: *p*_1_ = 230,569, *p*_2_ = 128,568, and *p*_3_ = 128,568 autosomal SNPs, respectively.

### Data analyses

All calculations PCA were carried out using Matlab’s (R2020a, Statistics and Machine Learning Toolbox Version 11.7) PCA function, which uses singular value decomposition (SVD), like SmartPCA, and yields nearly identical results to the basic SmartPCA tool^9^ (Version 7.2.1 without removing outliers, normalization, or projection) (Supplementary Figs. S1–S2).

In test cases where simulated data were used, we manipulated the colors and the sample size, both shown in each figure legend and caption. We evaluated the accuracy of PCA’s projections of the colors on a 2D plane as deviations from the true distances of the colors from each other on a 3D plane.

In test cases where human data were used, we modulated the choice of populations and sample size (individuals were always randomly sampled), both shown in each figure legend and caption. Dataset 1 was used to produce Supplementary Figs. S1–S2. All the human test cases were carried out on dataset 2, except of the case of ancient DNA, where the 3rd dataset was used. By large, we refrained from commenting on the accuracy of the prediction, even when it is well established, and instead focused on conflicting interpretations produced by PCA.

To evaluate the proportion of homogeneous clusters, we applied a *k*-means clustering (Matlab’s *kmean* function) to the two top PCs. Cluster homogeneity was calculated by using *k*-means clustering to PC1 and PC2 for *K* clusters (unless stated otherwise), where *k* was the square root of the number of samples. Clusters were considered homogeneous if they harbored only samples from one population.

### Evaluating missingness and noise

To evaluate the effects of missingness and noise in the case of marker choice, each color component was evenly divided across a window size of 200, generating a dataset of 600 “SNPs”. Missingness was then simulated by randomly nullifying different values of the matrix. The tri-color component structure was recovered by the reverse operation of summing the three 200-SNP-sets. The noise was generated by adding random markers (generated using Matlab’s *rand* function) to the color SNP set.

### Projection of ancient samples

A major challenge in projecting ancient samples onto modern-day samples is handling the high data absences. Lazaridis et al.^14^ addressed this problem using the least-squares projection (lsqproject) implemented in EIGENSOFT. Wang et al.^68^ cautioned that this method does not address the shrinkage problem (where all the projected samples cluster together) and that the results might be misleading. To avoid this problem and the difficulties associated with missing data, in the case of ancient DNA, we analyzed 65 out of 102 of the ancient samples of interest with over 10,000 SNPs in our dataset (with a median of 48,249 SNPs). We then projected one ancient sample at a time, based on the modern-day samples, using only the genotyped SNPs of the former.

### Estimating the citation number of PCA tools

Very conservatively, we estimate that, as of 4/2022, 32,000 genetic studies employed PCA based on Google Scholar’s citation count for the most commonly used PCA tools using the following searches: “EIGENSTRAT OR EIGENSOFT OR smartPCA” (8300), “PLINK AND PCA -EIGENSOFT -SNPRelate” (8390), “genalex AND PCA” (5990), “FlashPCA OR FlashPCA2” (365), “PCA in R AND genetics” (530), “adegenet AND PCA” (5350), ClustVis AND PCA (2170), and pcadapt AND PCA (624). A search for “(population Genetics) AND ("PCA")” yielded 159,000 results. This is also likely a small fraction of the true number of studies that employed PCA. Searching for “(Genetics OR genome) AND ("PCA")” yielded 216,000 results.

## Supplementary Information

Supplementary Information 1.

Supplementary Information 2.

## Data Availability

All our data and scripts that can replicate our results and figures are available via GitHub: https://github.com/eelhaik/PCA_critique.
