# Supplementary material for: Principal Component Analyses (PCA)-based findings in population genetic studies are highly biased and must be reevaluated
Source: Sci Rep. 2022 Aug 29;12:14683. doi: 10.1038/s41598-022-14395-4 (PMC9424212; doi:10.1038/s41598-022-14395-4)
Supplement: Supplementary file 1 — Supplementary Information 1. [file 41598_2022_14395_MOESM1_ESM.pdf]

Supplementary materials

Figure S1

The results of Matlab's PCA function (pca.m) (A) and SmartPCA (B) are nearly identical when applied to the Lazaridis et al. (2016) (N_{ind}=2068) dataset. The axes of (A) were divided by 2500 to match the axes of (B). Populations (N_{pop}=189) are colored differently. Selected populations are labeled.

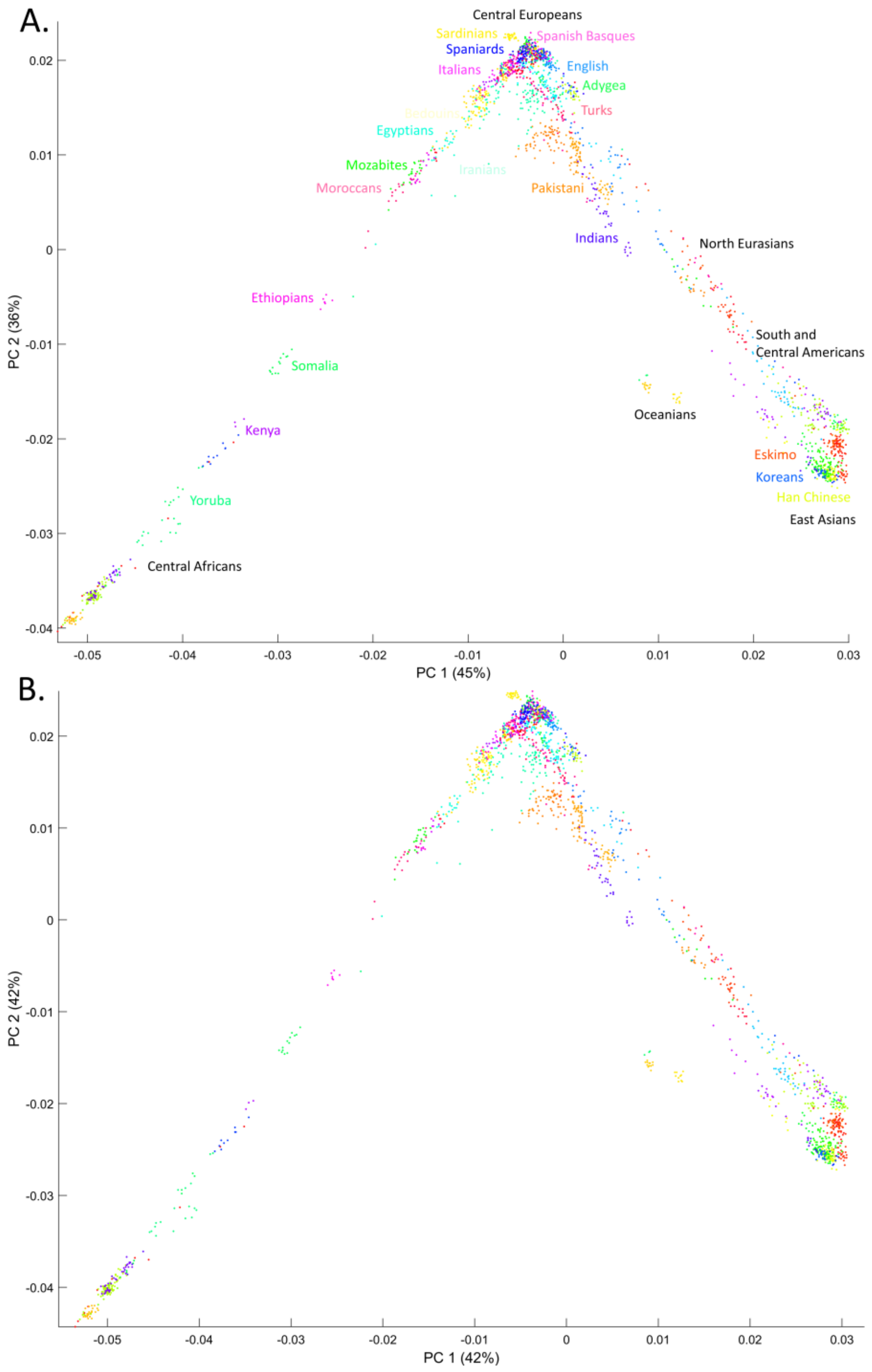

Figure S2
The first nine principal components (PCs) calculated by Matlab's pca.m (*x-axes*) and SmartPCA (*Y-axes*) are highly correlated when applied to the Lazaridis et al. (2016) (n_{ind}=2068) dataset (A-I). Pearson's correlation coefficients range from 0.99 to 1, and the *p*-values are 0 (two-tailed test).

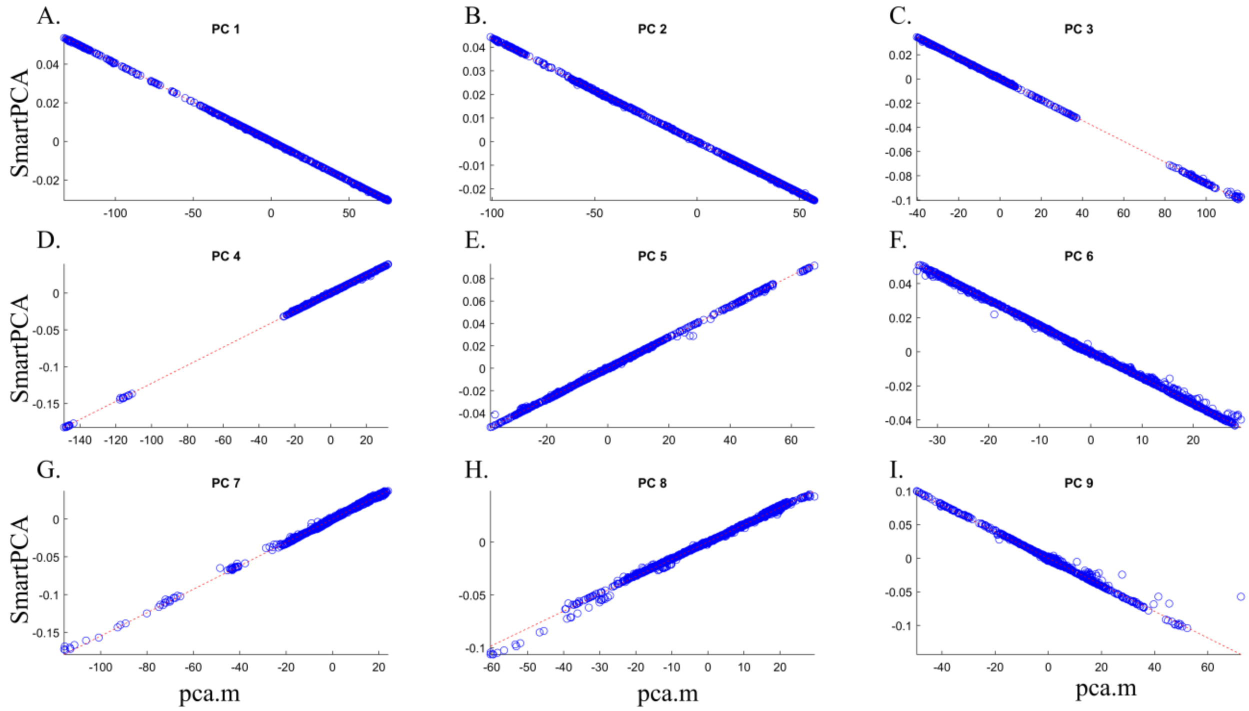

Figure S3

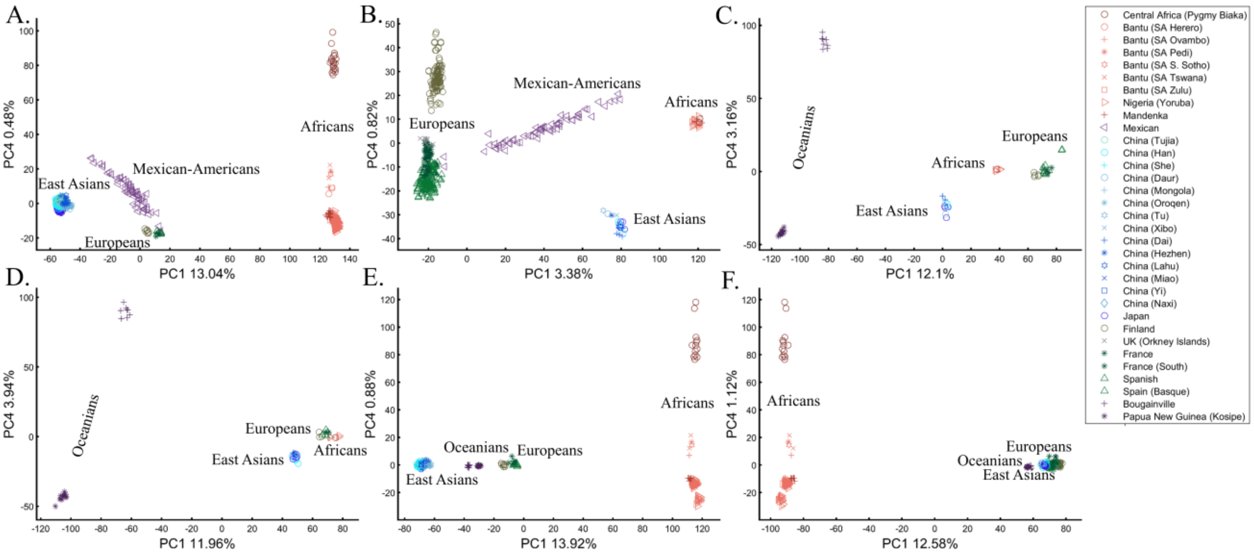

The figure legend is the same as in Figure 4, but showing PC1 and PC4.

Figure S4

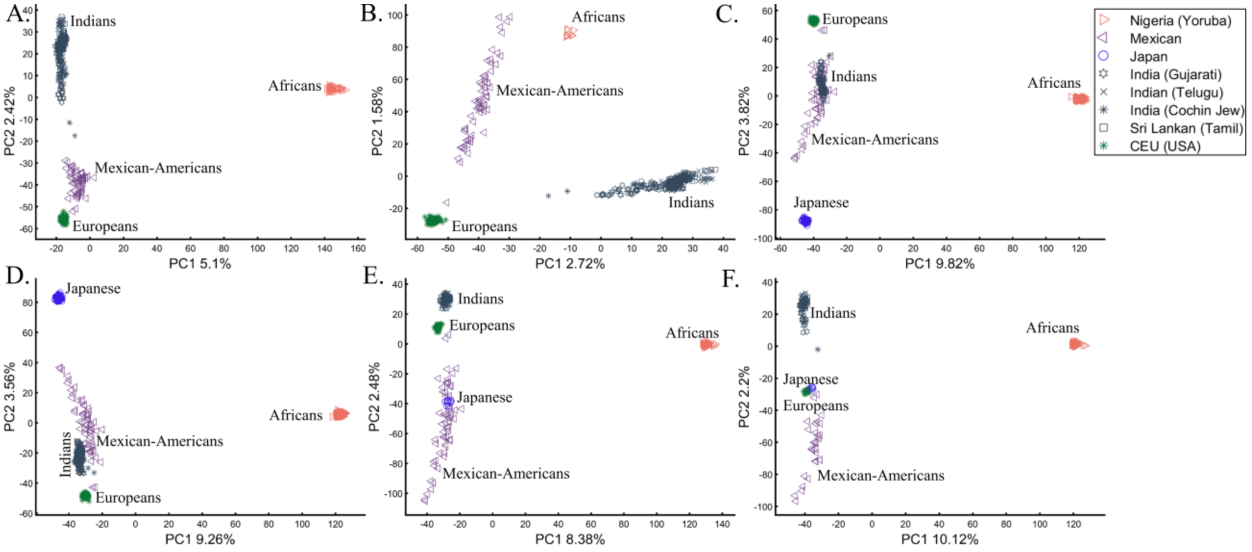

Figure S4. Generating alternative historical scenarios for the origin of Indians using PCA. A) Mexican-Americans as an admixed Indian-European group N_{Af}=50; N_{Eu}=90; N_{ME}=60; N_{Ind}=320. B) Africans as an admixed Mexican-American and Indian group, by reducing the African sample size of A: N_{Af}=10; N_{Eu}=90; N_{ME}=60; N_{Ind}=320, C) Indians and Mexican-American completely cluster N_{Af}=100; N_{Eu}=90; N_{ME}=60; N_{JP}=60, N_{Ind}=320, D) Indians and Mexican-American do not cluster N_{Af}=10; N_{Eu}=90; N_{ME}=60; N_{JP}=60, N_{Ind}=100, E) Japanese and Mexican-American cluster N_{Af}=50; N_{Eu}=50; N_{ME}=60; N_{JP}=10, N_{Ind}=100, and F) Japanese and European cluster N_{Af}=50; N_{Eu}=20; N_{ME}=30; N_{JP}=5, N_{Ind}=100

Figure S5

PCA for Black, the three primary colors, and an admixed Blue-Green color (Cyan). Varying the sample sizes yields different results, such as: A. Black is closer to Blue and Green and Cyan cluster (N_{Red}, N_{Green}, N_{Black}=100; N_{Blue}, N_{Cyan}=5), B. Black is closer to Green and Black and Cyan cluster (N_{Cyan}, N_{Green}=20; N_{Red}=1600; N_{Blue}, N_{Black}=400), C. Black clusters between Red and Blue, as expected from an admixed color (N_{Green}=10; N_{Black}=20; N_{Blue}=50; N_{Red}, N_{Cyan}=1000), and D. Black clusters between Red and Green, as expected from an admixed color (N_{Green}=50; N_{Black}, N_{Blue}=10; N_{Red}, N_{Cyan}=1000). Colors include Red [1,0,0], Green [0,1,0], Blue [0,0,1], Cyan [0,1,1], and Black [0,0,0].

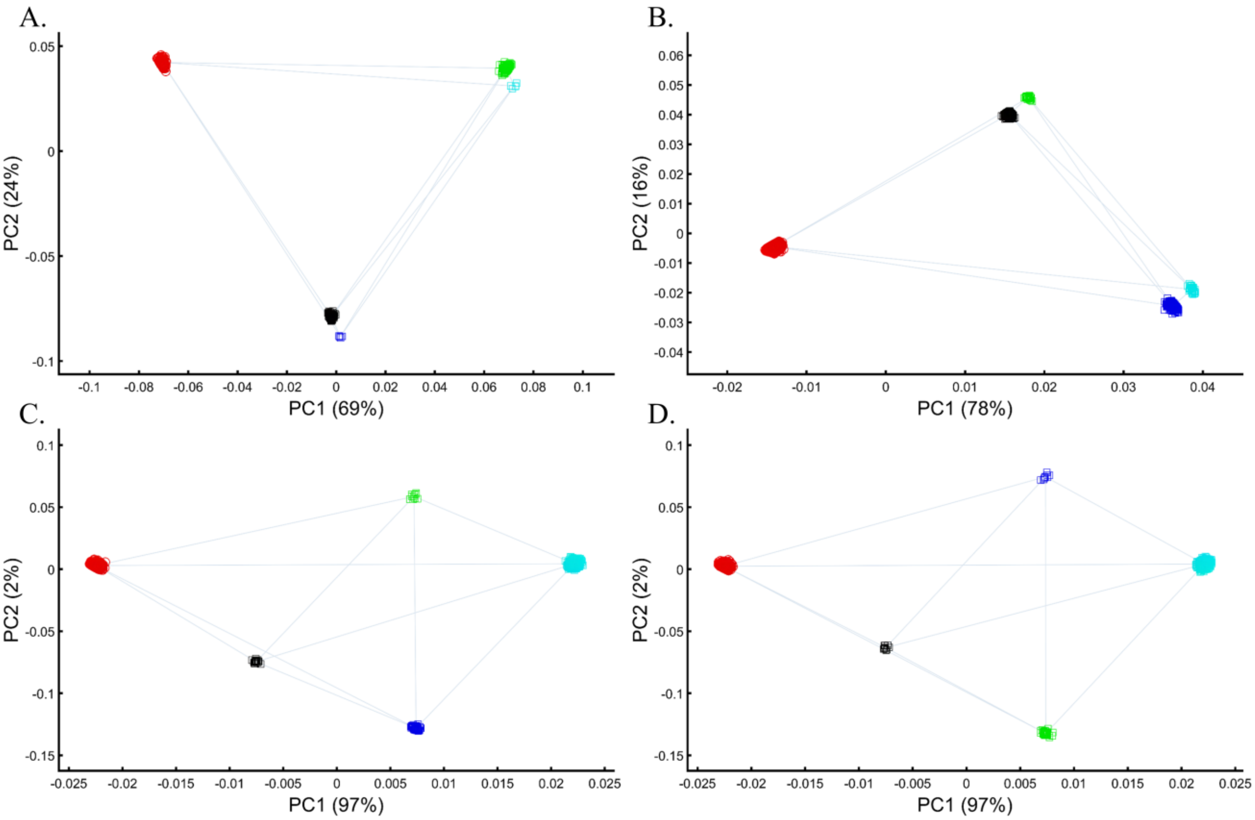

Figure S6

Plotting the second and third PCs for the samples in Figure S2.3.

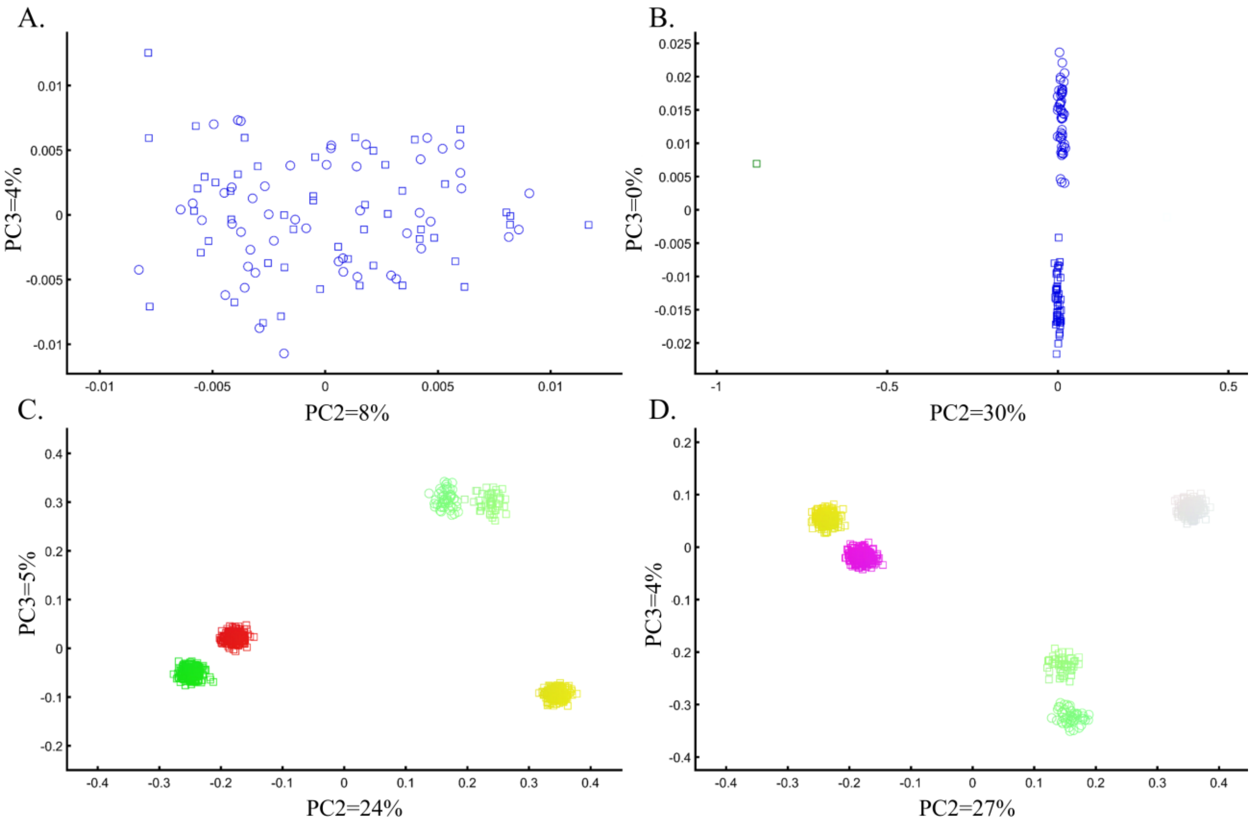

Figure S7

Plotting the first and third PCs for the samples in Figure S2.3.

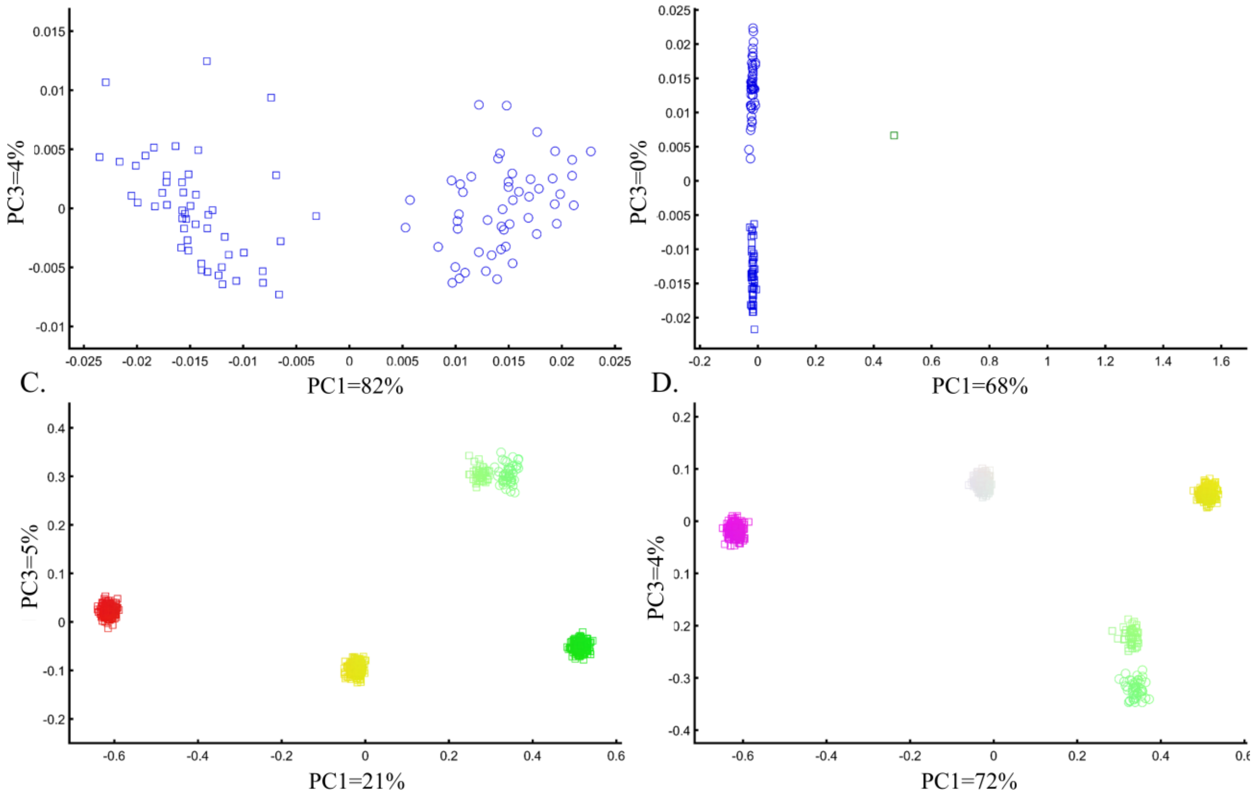

Figure S8

PCA for present-day populations. The PCA is performed on the same set of individuals as are reported in Figure 19, but without the ancient populations. Whereas in Figure 24, the modern-day populations used to project the ancient samples are greyed (to highlight the ancient samples), here they are colored.

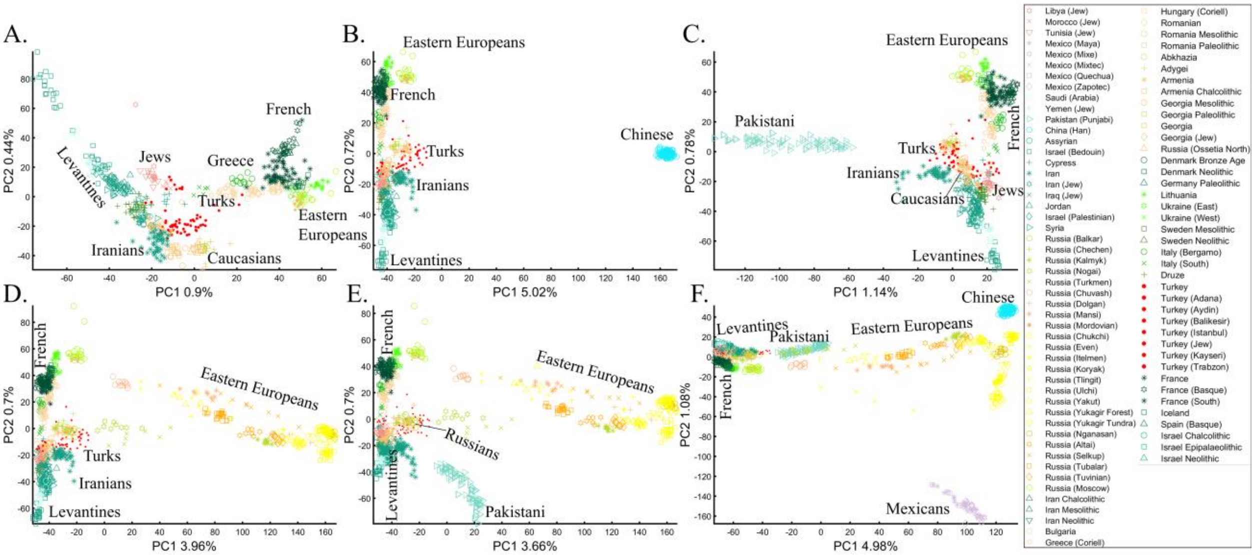

Figure S9

The proportion of explained variance by the first ten principal components (PCs) for a different number of samples (legend) selected at random from the entire dataset.

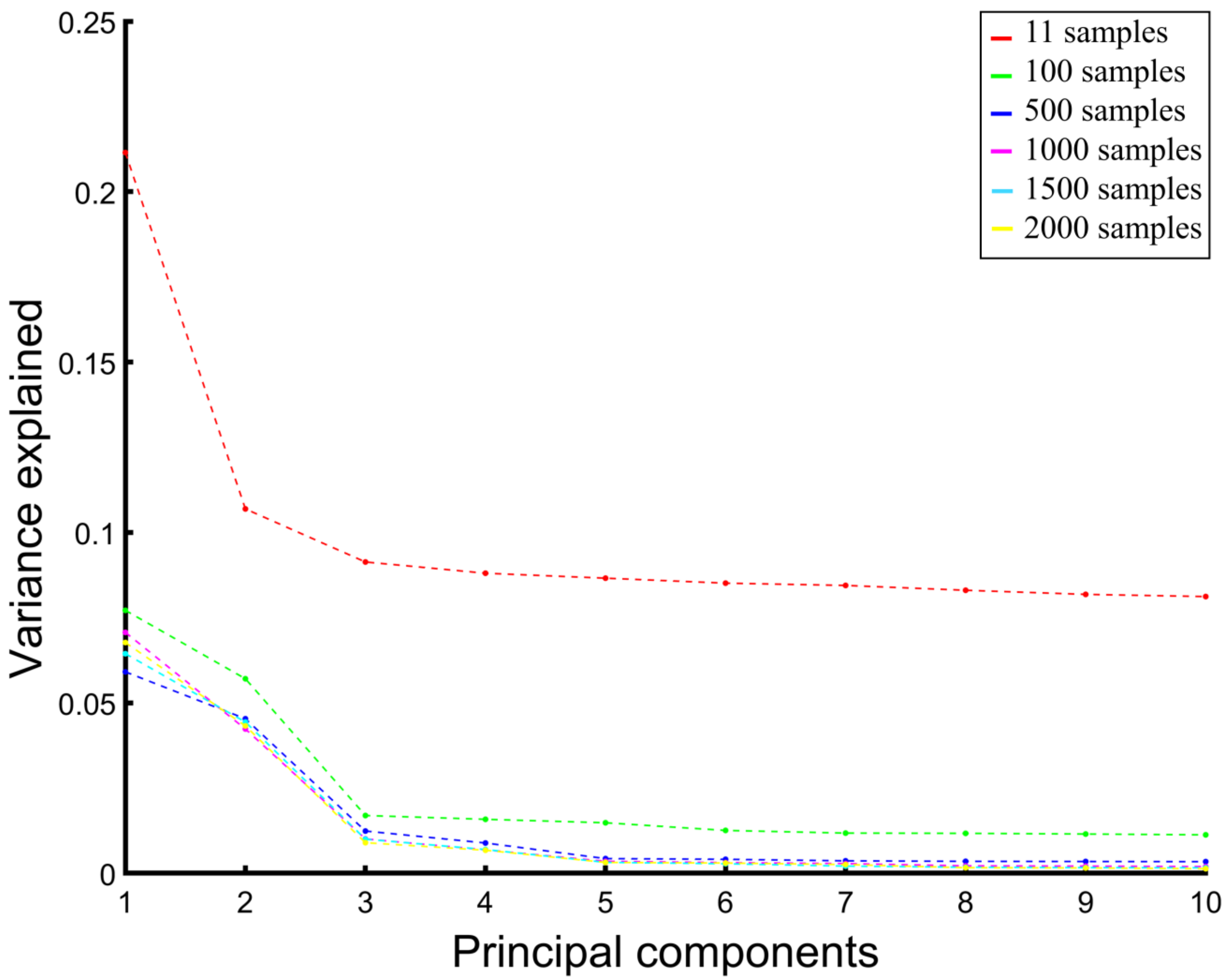

Figure S10

The proportion of the variance explained by the first two PCs for different numbers of individuals either from the same populations or selected at random from the entire dataset (inset).

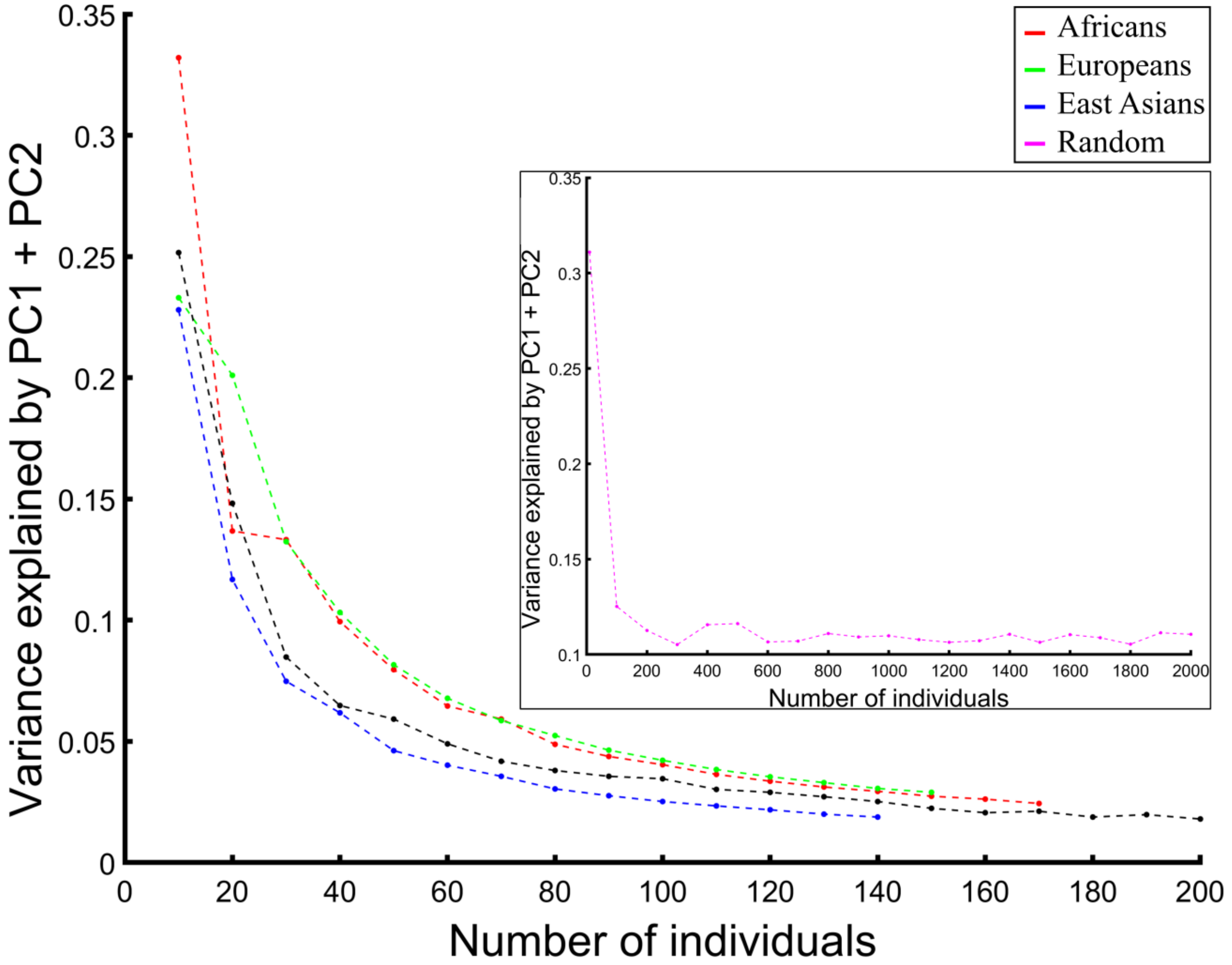

Figure S11

The proportion of explained variance due to the true structure of the data versus noise. Five 1000 Genomes Populations were selected from four continents: Europeans-Americans (CEU), Telugu Indians (ITU), Vietnamese (KHV), African-Americans (ASW), and Lima Peruvians (PEL). PCA was applied to 1200 random individuals and the reshuffled genetic dataset. We repeated the analyses 50 times. The proportion of variance explained by the first twenty PCs (x-axis) is shown in boxplots against the median proportion of variance calculated for the reshuffled matrix.

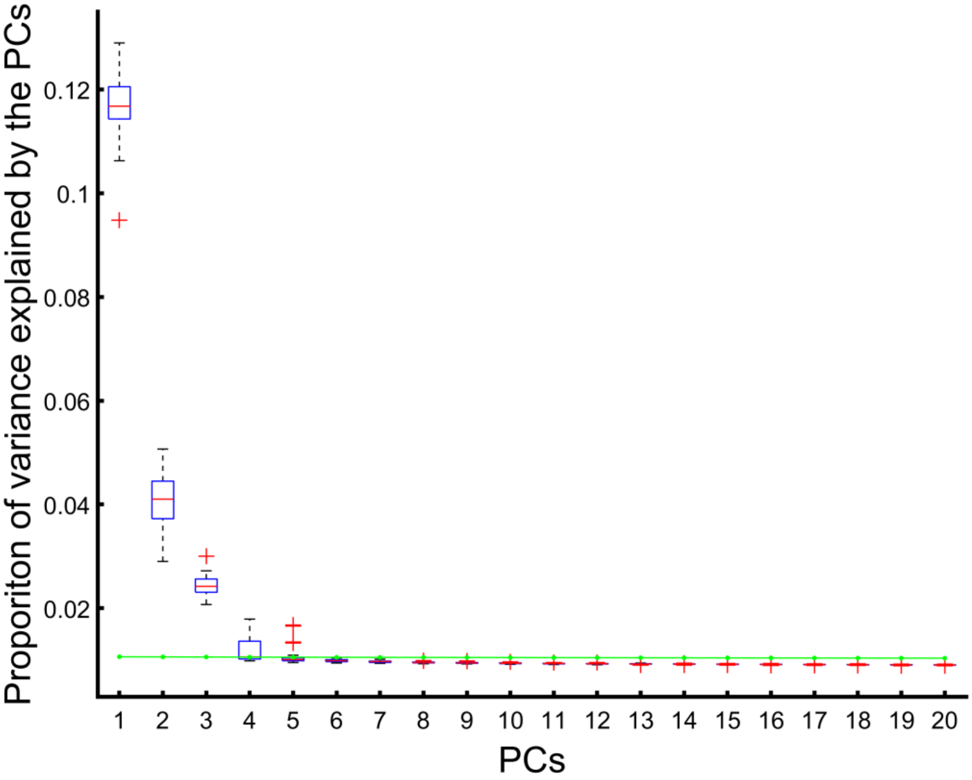

Figure S12

The effect of dimensionality on sample distances in PCA. Selecting 100 random Yoruba, we calculated the distances between the first two PCs for all individual pairs when applying PCA to 10, 100, 1000, 10,000, or 100,000 SNPs (A). We next calculated the cumulative shortest distance between the first two PCs for every sample (B) and the cumulative median distance for every sample (C) along the same number of SNPs: 10 (red), 100 (green), 1000 (blue), 10,000 (magenta), and 100,000 (cyan). Notice that some of the nearest neighbors analyzed in the highest dimension of 100,000 SNPs (B) have the same distance as the more distanced samples in C).

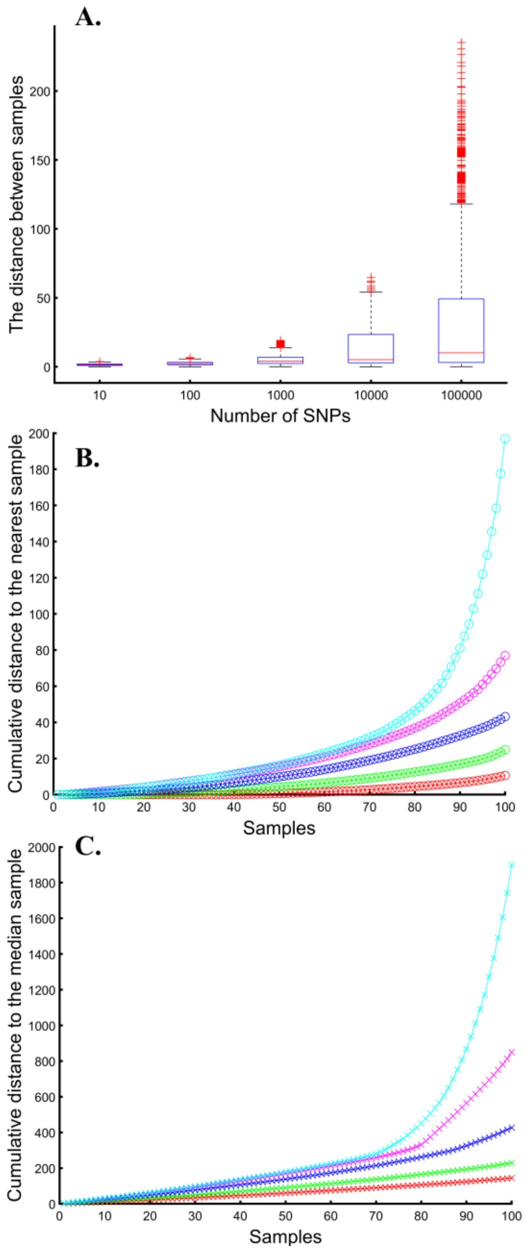

Table S1

Source of ancient DNA samples used in this study.

Sample Studied	Country	Publication
RISE61	Denmark	(Allentoft, et al. 2015)
RISE71	Denmark	(Allentoft, et al. 2015)
RISE94	Sweden	(Allentoft, et al. 2015)
RISE97	Sweden	(Allentoft, et al. 2015)
RISE98	Sweden	(Allentoft, et al. 2015)
WC1	Iran	(Broushaki, et al. 2016)
AH2	Iran	(Broushaki, et al. 2016)
AH1	Iran	(Broushaki, et al. 2016)
AH4	Iran	(Broushaki, et al. 2016)
oase1	Romania	(Fu, et al. 2015)
HohleFels49	Germany	(Fu, et al. 2016)
Falkenstein	Germany	(Fu, et al. 2016)
Falkenstein	Germany	(Fu, et al. 2016)
SC2	Romania	(González-Fortes, et al. 2017)
SC1	Romania	(González-Fortes, et al. 2017)
I5408	Romania	(González-Fortes, et al. 2017)
OC1	Romania	(González-Fortes, et al. 2017)
sf12	Sweden	(Günther, et al. 2018)
I1179	Israel	(Harney, et al. 2018)
I1152	Israel	(Harney, et al. 2018)
I1166	Israel	(Harney, et al. 2018)
I1178	Israel	(Harney, et al. 2018)
I1165	Israel	(Harney, et al. 2018)
I1160	Israel	(Harney, et al. 2018)
I1184	Israel	(Harney, et al. 2018)
I1170	Israel	(Harney, et al. 2018)
I1164	Israel	(Harney, et al. 2018)
I0644	Israel	(Harney, et al. 2018)
I1182	Israel	(Harney, et al. 2018)
I1169	Israel	(Harney, et al. 2018)
I1171	Israel	(Harney, et al. 2018)
I1168	Israel	(Harney, et al. 2018)
SATP	Georgia	(Jones, et al. 2015)
SATP	Georgia	(Jones, et al. 2015)
KK1	Georgia	(Jones, et al. 2015)
I0016	Sweden	(Lazaridis, et al. 2014)
I0017	Sweden	(Lazaridis, et al. 2014)
I1632	Armenia	(Lazaridis, et al. 2016)
I1631	Armenia	(Lazaridis, et al. 2016)
I1634	Armenia	(Lazaridis, et al. 2016)
I1407	Armenia	(Lazaridis, et al. 2016)
I1665	Iran	(Lazaridis, et al. 2016)
I1293	Iran	(Lazaridis, et al. 2016)

I1670	Iran	(Lazaridis, et al. 2016)
I1290	Iran	(Lazaridis, et al. 2016)
I1661	Iran	(Lazaridis, et al. 2016)
I1662	Iran	(Lazaridis, et al. 2016)
I1674	Iran	(Lazaridis, et al. 2016)
I1671	Iran	(Lazaridis, et al. 2016)
I0867	Israel	(Lazaridis, et al. 2016)
I1072	Israel	(Lazaridis, et al. 2016)
I0013	Sweden	(Mathieson, et al. 2015)
I0012	Sweden	(Mathieson, et al. 2015)
I0011	Sweden	(Mathieson, et al. 2015)
I0015	Sweden	(Mathieson, et al. 2015)
I0014	Sweden	(Mathieson, et al. 2015)
I4081	Romania	(Mathieson, et al. 2018)
I4582	Romania	(Mathieson, et al. 2018)
I4607	Romania	(Mathieson, et al. 2018)
I5411	Romania	(Mathieson, et al. 2018)
I5436	Romania	(Mathieson, et al. 2018)
Saxtorp5164	Sweden	(Mittnik, et al. 2018)
Ajvide58	Sweden	(Skoglund, et al. 2014)
Ajvide70	Sweden	(Skoglund, et al. 2014)
Gokhem2	Sweden	(Skoglund, et al. 2014)

REFERENCES

Allentoft ME, Sikora M, Sjogren KG, Rasmussen S, Rasmussen M, Stenderup J, Damgaard PB, Schroeder H, Ahlstrom T, Vinner L, et al. 2015. Population genomics of Bronze Age Eurasia. Nature 522:167-172.
Broushaki F, Thomas MG, Link V, Lopez S, van Dorp L, Kirsanow K, Hofmanova Z, Diekmann Y, Cassidy LM, Diez-del-Molino D, et al. 2016. Early Neolithic genomes from the eastern Fertile Crescent. Science 353:499-503.
Fu Q, Hajdinjak M, Moldovan OT, Constantin S, Mallick S, Skoglund P, Patterson N, Rohland N, Lazaridis I, Nickel B, et al. 2015. An early modern human from Romania with a recent Neanderthal ancestor. Nature.
Fu Q, Posth C, Hajdinjak M, Petr M, Mallick S, Fernandes D, Furtwängler A, Haak W, Meyer M, Mittnik A, et al. 2016. The genetic history of Ice Age Europe. Nature 534:200–205.
González-Fortes G, Jones ER, Lightfoot E, Bonsall C, Lazar C, Grandal-d'Anglade A, Garralda MD, Drak L, Siska V, Simalcsik A, et al. 2017. Paleogenomic Evidence for Multi-generational Mixing between Neolithic Farmers and Mesolithic Hunter-Gatherers in the Lower Danube Basin. Current Biology 27:1801-1810.e1810.
Günther T, Malmstrom H, Svensson EM, Omrak A, Sanchez-Quinto F, Kilinc GM, Krzewinska M, Eriksson G, Fraser M, Edlund H, et al. 2018. Population genomics of Mesolithic Scandinavia: Investigating early postglacial migration routes and high-latitude adaptation. PLoS Biology 16:e2003703.
Harney É, May H, Shalem D, Rohland N, Mallick S, Lazaridis I, Sarig R, Stewardson K, Nordenfelt S, Patterson N, et al. 2018. Ancient DNA from Chalcolithic Israel reveals the role of population mixture in cultural transformation. Nature Communications 9:3336.
Jones ER, Gonzalez-Fortes G, Connell S, Siska V, Eriksson A, Martiniano R, McLaughlin RL, Gallego Llorente M, Cassidy LM, Gamba C, et al. 2015. Upper Palaeolithic genomes reveal deep roots of modern Eurasians. Nature Communications 6:8912.
Lazaridis I, Nadel D, Rollefson G, Merrett DC, Rohland N, Mallick S, Fernandes D, Novak M, Gamarra B, Sirak K, et al. 2016. Genomic insights into the origin of farming in the ancient Near East. Nature 536:419-424.
Lazaridis I, Patterson N, Mittnik A, Renaud G, Mallick S, Kirsanow K, Sudmant PH, Schraiber JG, Castellano S, Lipson M. 2014. Ancient human genomes suggest three ancestral populations for present-day Europeans. Nature 513:409-413.
Mathieson I, Alpaslan-Roodenberg S, Posth C, Szécsényi-Nagy A, Rohland N, Mallick S, Olalde I, Broomandkhoshbacht N, Candilio F, Cheronet O, et al. 2018. The genomic history of southeastern Europe. Nature 555:197-203.
Mathieson I, Lazaridis I, Rohland N, Mallick S, Patterson N, Roodenberg SA, Harney E, Stewardson K, Fernandes D, Novak M, et al. 2015. Genome-wide patterns of selection in 230 ancient Eurasians. Nature 528:499-503.
Mittnik A, Wang C-C, Pfrengle S, Daubaras M, Zariņa G, Hallgren F, Allmäe R, Khartanovich V, Moiseyev V, Tõrv M, et al. 2018. The genetic prehistory of the Baltic Sea region. Nature Communications 9:442.
Skoglund P, Malmstrom H, Omrak A, Raghavan M, Valdiosera C, Gunther T, Hall P, Tambets K, Parik J, Sjogren KG, et al. 2014. Genomic diversity and admixture differs for Stone-Age Scandinavian foragers and farmers. Science 344:747-750.
