# Supplementary material for: Principal Component Analyses (PCA)-based findings in population genetic studies are highly biased and must be reevaluated
Source: Sci Rep. 2022 Aug 29;12:14683. doi: 10.1038/s41598-022-14395-4 (PMC9424212; doi:10.1038/s41598-022-14395-4)
Supplement: Supplementary file 2 — Supplementary Information 2. [file 41598_2022_14395_MOESM2_ESM.pdf]

Supplementary Texts 1-3

Supplementary Text 1 – Testing how PCA represents mixed and unmixed populations via a simulation... 2

Supplementary Text 2: Two more applications of PCA ... 4

2.1 The case of two or three-way admixed population ... 4

2.2 The case of pairwise comparisons ... 7

Supplementary Text 3: Evaluating the accuracy of correcting for population stratification with PCA ... 10

References ... 14

Supplementary Text 1 – Testing how PCA represents mixed and unmixed populations via a simulation

To test whether admixture can be inferred from the positioning of populations in a PCA plot, we simulated six ancestral populations with 20 cohorts per population over 50 markers. Allele frequencies (*AF*) per ancestral population per marker were generated at random. For each cohort, the allele frequencies for marker *i* was calculated as:

(1) $AF_i + 0.01*R_N$

with R_N being a random number generated by a standard normal distribution. Colors were randomly assigned to the parental populations, which were marked with circles. Cross-population inbreeding was set to 20% per generation. Mixed cohorts were crosses between two mixed cohorts or mixed and unmixed cohorts. Unmixed cohorts were sampled within the cohort. All F1 cohorts were generated by averaging the allele frequencies of their parental cohorts. F1 cohorts also received the average colors of their parents and were marked with x. To calculate F2 cohorts, we repeated the analysis with the F1 cohorts replacing the parental cohorts. In such a manner, we calculated multiple generations with constant sample size. We calculated PCA for each generation, plotting the parental- and the next-generation cohorts over the primary two PCs. Convex hull was applied to the first two PCs to identify the corners.

In the initial generations, the ancestral populations were positioned in all corners, but not all the ancestral populations were at the corners. In Figure S1.1A, the red and cyan ancestral populations are positioned at the hull's center, undistinguished from the mixed cohorts. This pattern continued until the sixth generation (Figure S1.1F). By then, one of the ancestral populations had gone extinct, and an admixed population took the corner. This process was not surprising because admixture populations were already positioned along the edges (Figure S1.1A-H). It was only a matter of time until the disappearance of one of the original populations would change the hull's shape. Remarkably, this process occurred while some of the unmixed parental populations remained in the hull's center. By the eighth generation, half of the corner populations were admixed cohorts (Figure S1.1H).

These results were replicated over multiple simulations, irrespective of the number of parental populations, sample sizes, marker sizes, and mixture proportions. Modulating some of these factors expedited or slowed the process, but the ultimate results were the same.

In summary, even in simple simulations that do not capture the complexity of genomic data and life processes, it is impossible to deduce whether populations are ancestral (unmixed) or mixed based on their position in a PCA plot. Furthermore, while simulations may show only some of the ancestral populations in the corners of an imaginary hull fit to the primary PCs, this effect is temporary. After several generations, unmixed populations will reach the corners. These findings are in agreement with our observations where admixed populations (e.g., Ashkenazic Jews) appeared both at the corner (e.g., Figure 11C) and the center (e.g., Figure 11A) of PCA plots.

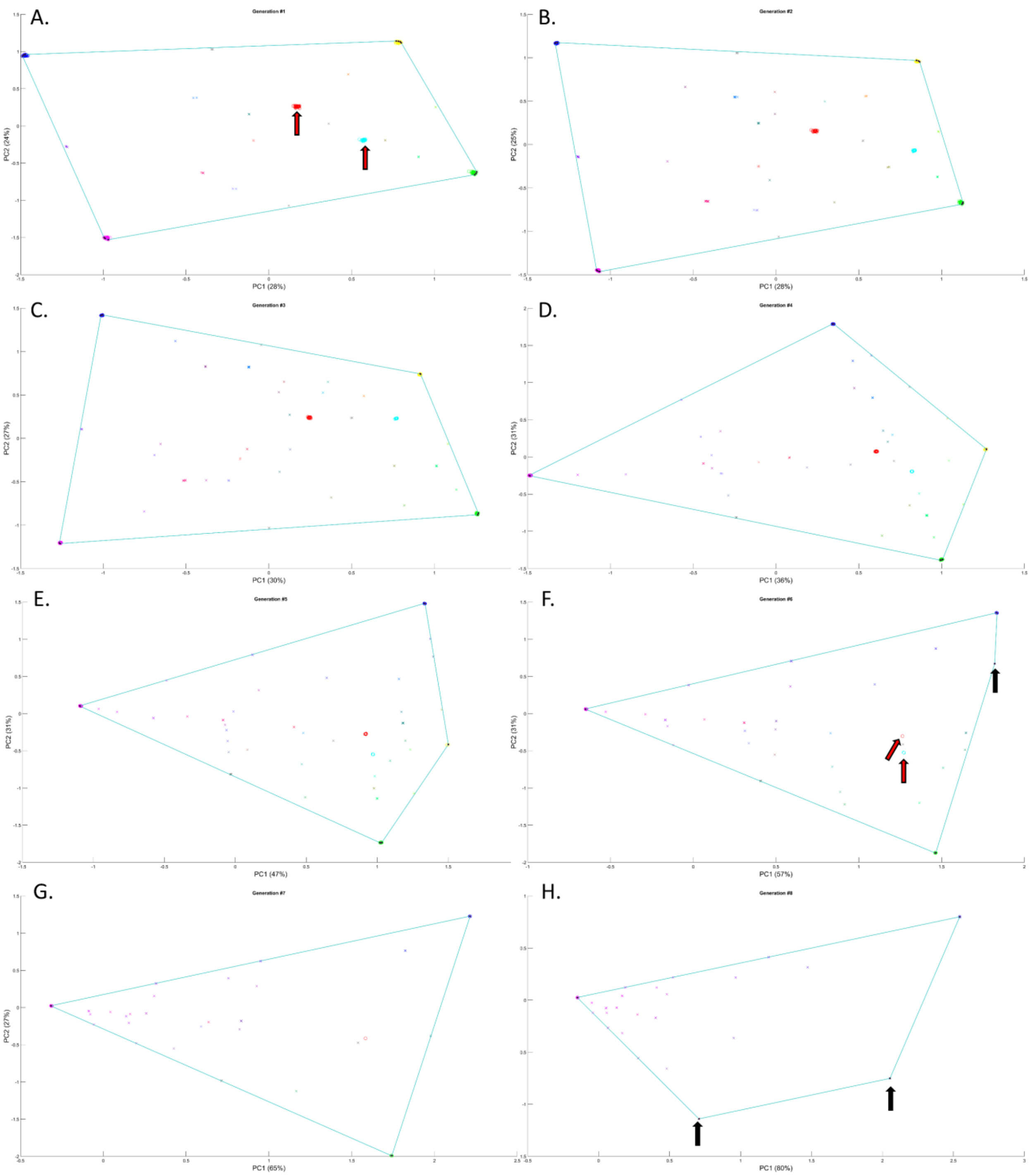

Figure S1.1. Forward simulation over eight generations. Each plot shows 120 parental populations and 120 offspring. Mixed (circles) and unmixed (x) cohorts are marked. Offspring inherit the colors of their parents. Convex hulls are marked by lines, and corners are marked with black circles. Red arrows point at some of the unmixed cohorts, and black ones at some of the mixed cohorts. PCA results in each of eight generations are shown.

Supplementary Text 2: Two more applications of PCA

2.1 The case of two or three-way admixed population

The question of how analyzing admixed groups with two or three ancestral populations affects findings for unmixed groups is illustrated through a typical study case in Box S2.1.

Box S2.1 – Studying the origin of Black using the primary and secondary colors

Though the previous analyses could not resolve the origin of Black (Box 2), there was a consensus that admixed cohorts can provide novel insights even though the issue of sampling bias remained unaddressed. An even-sample analysis (n=10) that included Cyan and Purple supported earlier suspicions that Black is a Green-Red admix (Figure S2.1A). Concerned with the low sample sizes, the Black-is-Green group increased the Red, Blue, and Purple sample sizes (n_{Red}, n_{Blue}, n_{Purple}=100), which both showed that Black is closer to Green and that Cyan is closer to Blue (Figure S2.1B). The Black-is-Blue group argued that admixed individuals should be sampled at lower numbers (n_{Cyan}, n_{Purple}=5) and since Blue and Green shared common origins (Figure 5D), they are interchangeable and should be sampled in an even amount to Red and Black (n_{Green} + n_{Blue}, n_{Red}, n_{Black}=50). The results showed perfect Black-Blue, Green-Cyan, and Red-Purple overlap (Figure S2.1C), at odds with the historical records of the origin of secondary colors. To test that, an independent group ventured into the field and collected Yellow. The results positioned Cyan and Purple close to their postulated ancestral cohorts and yielded a new genuine insight that Black is Yellow (Figure S2.1D). The Black-is-Red group did not dispute this claim but argued that Yellow is a recent Black admixture and that Yellow should be excluded from future analyses to gain a true understanding of Black's ancient origins. The removal of Yellow from the analysis showed a complete Black-Red overlap and concrete evidence that Black has a Red origin (Figure S2.1E). Yet those results could not be accepted as follow-up analyses provided credence to a new novel finding proving that Black has originated from Cyan (Figure S2.1F). To consolidate these different observations, the groups gathered together and a new consensus has emerged. Since Black clustered mostly with Green, which is a shade of Blue (Figure 5D), it was concluded that Black is the ancestor of Green and Blue, which partially explained many of the conflicting results reported thus far. That Green and Cyan clustered together (Figure S2.1C) supported the idea that Black is part of the Green-Blue-Cyan clade (Figure S2.1F). Since Cyan was positioned in the corner of the PC plot in all analyses (Figure S2.1), it also became established that it is a novel primary color. Not everyone agreed with these conclusions.

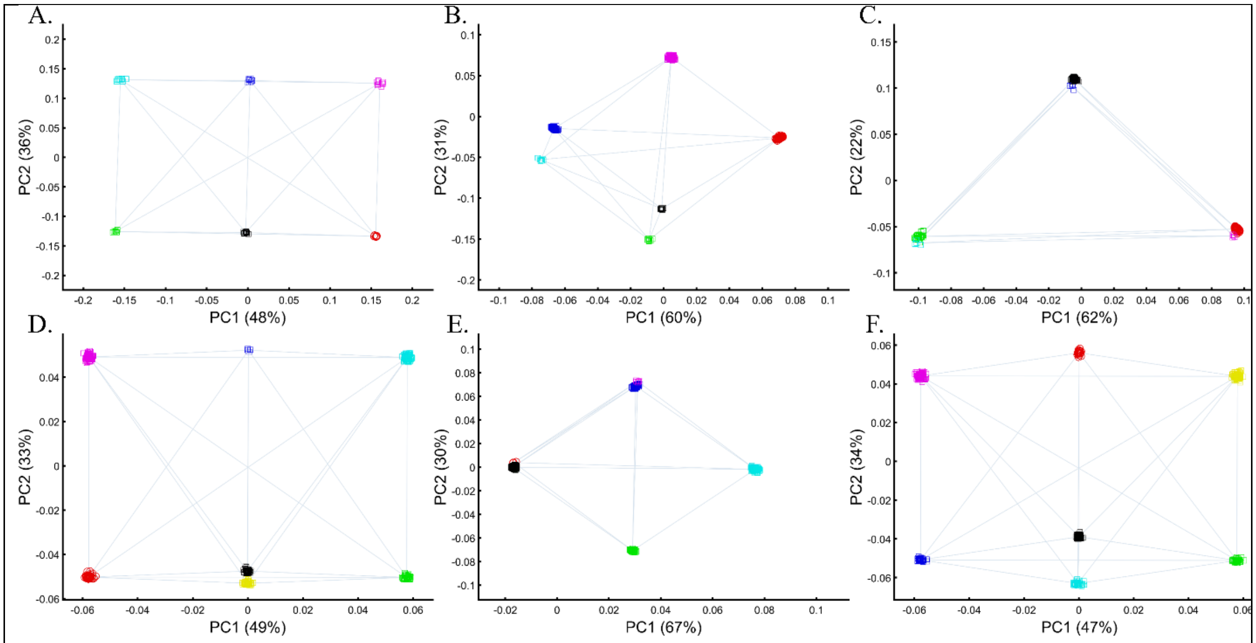

Figure S2.1. PCA with the primary and two or three mixed color populations. A) n_{all}=100, B) n_{Red}=n_{Blue}=n_{Purple}=100; n_{Green}=n_{Black}=n_{Cyan}=10, C) n_{Red}=n_{Black}=50; n_{Blue}=n_{Cyan}=n_{Purple}=5; n_{Green}=45, D) n_{Red}=n_{Green}=n_{Black}=n_{Yellow}=50; n_{Blue}=5; n_{Purple}=n_{Cyan}=100, E) n_{Black}=800; n_{Red}=n_{Purple}=50; n_{Blue}=n_{Green}=n_{Cyan}=100, F) n_{Red}=25; n_{Green}= n_{Blue}=n_{Black}=n_{Cyan}=50; n_{Purple}=n_{Yellow}=100. Scatter plots show the top two PCs. Colors include Red [1,0,0], Green [0,1,0], Blue [0,0,1], Cyan [0,1,1], Purple [1,0,1], Yellow [1,1,0], and Black [0,0,0].

The origin of Papuans and Bouganvilleans or Northern Melanesians (NMs) has been extensively investigated in the literature[1]. PCA yielded conflicting results not only about their distance to other samples but to each other. When analyzed against East Asians and Oceanians, Papuans and Bouganvilleans clustered at the edge of the plot either together but separately from other samples[2] or separately from each other and other samples[3]. Perhaps consequently, Papuan ancestry is considered distinct from Asian ancestry, which, in turn, is considered distinct from non-Asian ancestries[1]. However, this is not obvious from PCA studies, as Papuans were also shown to cluster with Amerindians and close to Central-Southern Asia[4].

It is easy to show how PCA can be used to generate conflicting results when NMs are analyzed alongside other highly admixed groups. We first show that when using even-sampling, the inclusion of NMs creates a European-Asian cluster that was not observed before (Figure S2.2A) with NMs clustering separately from one another at the extreme edge with East Asians clustering along a "European-Oceanian cline," appearing as a Europeans-NMs admixed group. Using uneven sampling, as done in all the studies, yields various contradictory results with NMs clustering together and close to East Asians (Figure S2.2B), appearing as a three-way tri-continental admix group distinct from each other and close to Pakistani (Hazara) (Figure S2.2C), or remaining highly distinct and separate from other population but influencing the formation of an African-European cluster with Pakistani (Hazara) as an outgroup (Figure S2.2D). Each of these results supports a different explanation for the origin of NMs, all equally mathematically

valid, which is a biological implausibility. These examples show that PCA can produce results that may appear, in part, meaningful (i.e., Africans are separated from non-Africans in Figure S2.2A-C) based on our *a priori* knowledge. Still, even in this case, they provide biologically meaningless and contradictory outcomes.

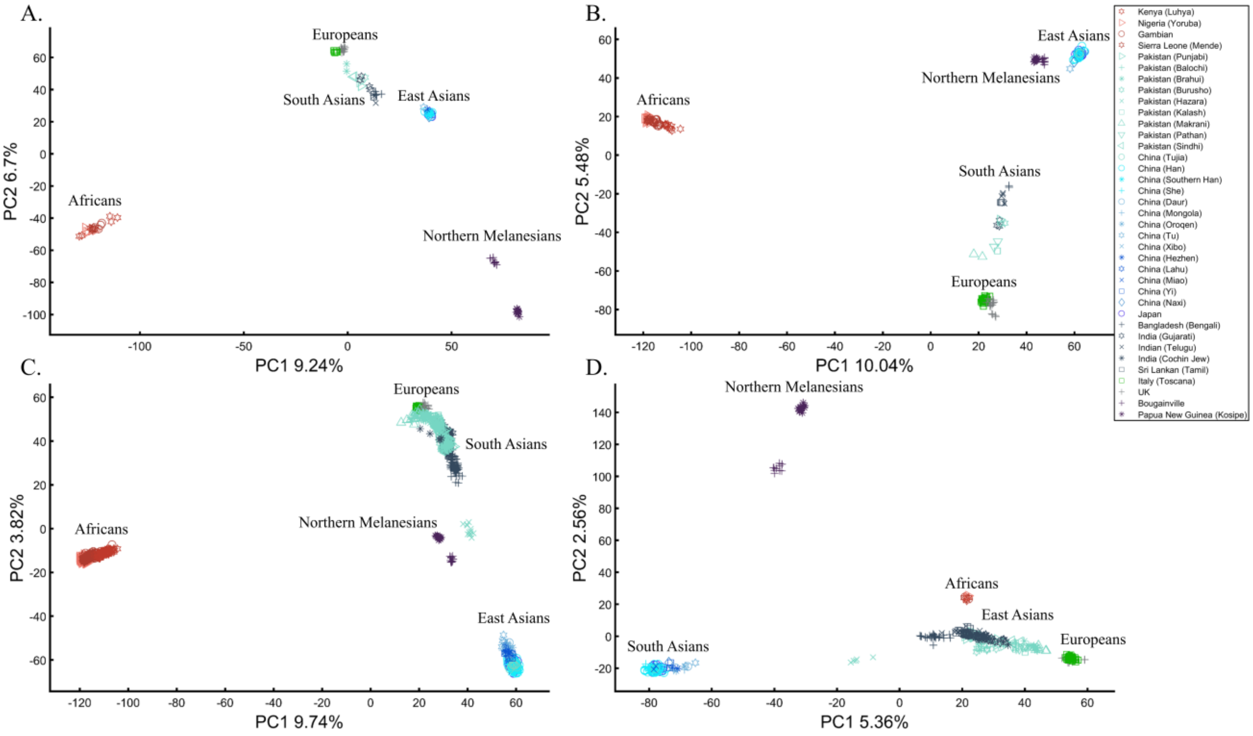

Figure S2.2. Studying the origin of Northern Melanesians using PCA. Five populations are analyzed: Africans (Af), Europeans (Eu), East Asians (EA), South Asians (SA), and Northern Melanesians (NM). Results vary based on the sample size of each population. A) n_{all}=20, B) n_{Af}=n_{Eu}=n_{EA}=50; n_{Sa}=n_{nM}=20, C) n_{Af}=n_{EA}=300; n_{Eu}=50; n_{Sa}=500; n_{nM}=24, D) n_{Af}=10; n_{Eu}=60; n_{EA}=100; n_{Sa}=200; n_{nM}=24.

2.2 The case of pairwise comparisons

Several authors adopted a pairwise comparison scheme to assess the genetic similarity between two cohorts of interest, e.g.,[5,6-8]. This setting is prevalent in case-control analyses that seek overlap between the compared groups (e.g., cases and controls[9-12]). We assessed whether this setting could lead to erroneous conclusions by analyzing two non-overlapping color populations (Figure S2.3A). We found that in the presence of two other samples, they highly overlap (Figure S2.3B) and may appear as part of the same cohort, thus altering the conclusions. Moreover, the latter analysis explains 99% of the variation compared to the former (94%), which may appear more reliable. We next analyzed two cohorts of interest alongside other populations. We found that whether the two cohorts overlap or not depends on the choice of the other populations (Figure S2.3C-D).

Further analysis of the second and third PCs (Figure S6) for the same populations in Figure S2.3 showed that the two Blue sheds overlap in both cases. By contrast, the two Green sheds did not, though both were closer to Red and Yellow than Green, their closest color population. Finally, an analysis of the first and third PCs (Figure S7) showed that the Blue and Green sheds are separate in both cases. In the latter case, the Green shades were closer to both the Green and Yellow populations. Overall, these examples show that PCA outcomes as to whether populations overlap are independent of whether or not the populations are distinct. Instead, they are an artifact of the sampling scheme, for which no rules exist. These examples demonstrate how, when applied in a pairwise setting, PCA can lead to erroneous conclusions concerning clustering, identity, and distance cross-dimensionally.

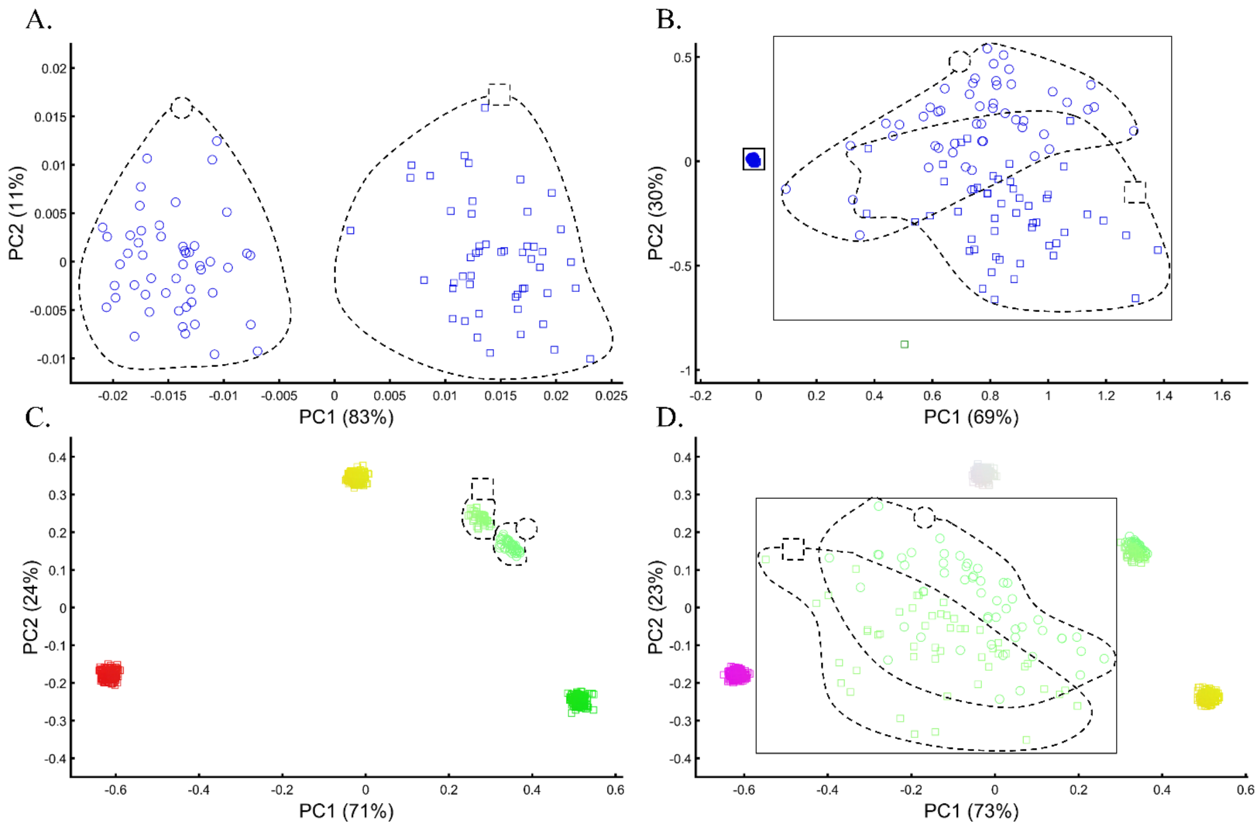

Figure S2.3. Using PCA in a pairwise setting to assess the similarity between two color-population cohorts. Even-size cohorts (*n*=50) of two distinct shades of Blue (circles and squares) do not overlap (A) and mostly overlap (B) when analyzed along with Green and White samples. Even-sized cohorts (*n*=50) of two distinct shades of Green (circles and squares) do not overlap when analyzed with three even-sized (*n*=250) populations (C) but overlap when analyzed with other even-sized (*n*=250) populations (D). Colors include Red [1,0,0], Green [0,1,0], Purple [1,0,1], Yellow [1,1,0], and two shades of Blue ([0.025,0,1], [0,0.025,1]) and Green: ([0.5,1,0.5], [0.6,1,0.5]).

We next tested the performance of PCA in pairwise settings in human populations. In Figure S2.4A, we show that two Chinese populations, which PCA purports are a single homogeneous population, can be split if Japanese are included in the sampling scheme (Figure S2.4B). That is, PCA's outcome for the genetic relationships between Dai and Southern Han Chinese are relative to the inclusion of another population. Likewise, PCA's answer to whether Mexicans and Peruvians share common origins and are comparable in this setting depends on the presence of Africans in the scheme (Figure S2.4C-D), which determines the genetic relationships between the two populations, as per PCA. These examples show that PCA clustering and distances are based on the choice of the reference populations or merely their inclusion or exclusion in an unpredictable manner. PCA results are unreliable when studying the relationships between populations in a pairwise scheme.

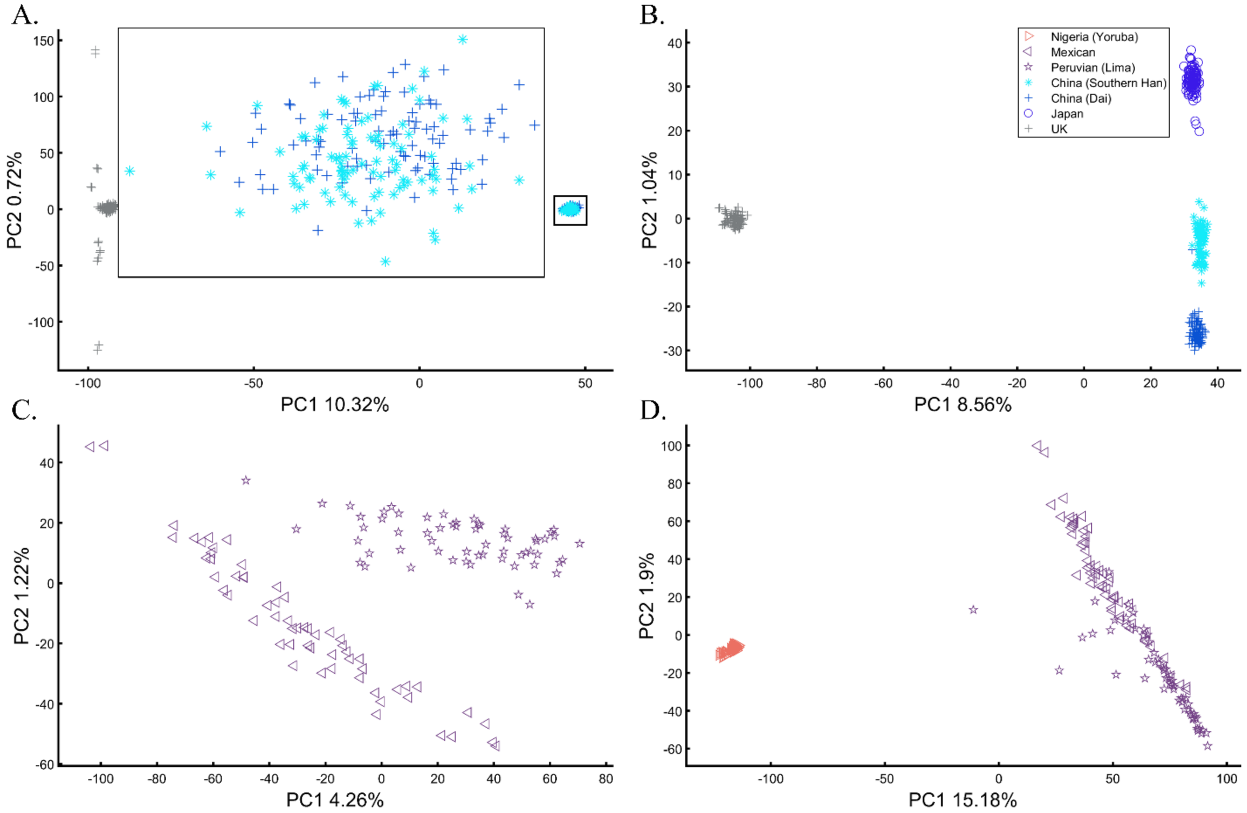

Figure S2.4. Evaluating the reliability of cohort clustering to assess their homogeneity. Southern Han and Dai Chinese appear overlapping when analyzed with UK samples (A) but completely distinct when analyzed alongside Japanese (B). Lima Peruvians and Mexican-Americans cluster distinctively from each other (C) but completely overlap when analyzed with Africans (D). The large square indicates an inset.

Supplementary Text 3: Evaluating the accuracy of correcting for population stratification with PCA

To test whether correcting for population stratification using PCA in a case-control association study improves the accuracy of the results, i.e., identifies the causal markers, we analyzed a dataset of 300 Europeans and 20 Puerto Ricans genotyped over ~129,000 markers as described in the main text. The Europeans were randomly divided into two groups and assigned random disease status. The Puerto Ricans were all assigned to the control group. Overall, we created two equally sized groups of 160 samples each. Ten markers were modified to be causal markers by randomly selecting genotypes that differ in frequencies between the cases and controls using the weights described in Table S3.1. We carried out six analyses, testing five dominant and one heterozygote model (Table S3.1).

		Cases			Controls		
Analysis	Model	AA	AB	BB	AA	AB	BB
1	Dominant	0.2	0.5	0.3	0.4	0.5	0.1
2	Dominant	0.5	0.4	0.1	0.45	0.25	0.3
3	Dominant	0.4	0.2	0.4	0.5	0.4	0.1
4	Dominant	0.3	0.2	0.5	0.5	0.4	0.1
5	Dominant	0.4	0.4	0.2	0.3	0.3	0.4
6	Heterzygote	0.25	0.5	0.25	0.35	0.3	0.35

Table S3.1. Genotype weights used to generate causal markers in six analyses of case-control association studies.

After generating the casual markers, we performed the logistic regression analysis using PLINK 2.0 (www.cog-genomics.org/plink/2.0/)[13] with correction for multiple testing using the Benjamini & Yekutieli (2001)[14] step-up false discovery control before and after adjusting for ten and two principal components. Our findings are shown in Table S3.2.

Comparing the results before and after PCA adjustment shows the advantage of avoiding PCA adjustment in all the analyses. PCA-adjusted results consistently yielded more false negatives and lower significance values for both ten and two PCs and dominant and heterozygote models. This is particularly notable in the fifth analysis, where the two significant causal markers were lost after PCA adjustment. The fourth analysis allows comparing between the *p*-value reported before and after PCA adjustment (Figure S3.1). The *p*-values before PCA adjustment were lower than those after adjusting for two PCs, with the highest *p*-values calculated after adjusting for ten PCs. Similar results were obtained for random assignment of case-control status to all the samples (results not shown).

		Chr	ID	Unadjsted	FDR (BY)	Is causal SNP
**Analysis 1**	**No PCA correction**					
		1	rs2765021	1.04E-10	1.62E-04	Yes
		12	rs10844115	1.04E-07	8.10E-02	No
		1	rs6603803	1.67E-07	8.63E-02	Yes
	**PCA (10 PCs)**					
		1	rs2765021	1.64E-10	2.55E-04	Yes
		2	rs309137	8.17E-08	6.35E-02	No
		12	rs10844115	1.62E-07	8.41E-02	No
	**PCA (2 PCs)**					
		1	rs2765021	2.00E-10	3.11E-04	Yes
		12	rs10844115	6.04E-08	0.0469269	No
**Analyasis 2**	**No PCA correction**					
		1	rs2765021	8.11E-13	7.89E-07	Yes
		1	rs13303344	1.02E-12	7.89E-07	Yes
		1	rs7511905	1.96E-12	1.02E-06	Yes
		1	rs3813199	1.85E-11	6.63E-06	Yes
		1	rs6685064	2.13E-11	6.63E-06	Yes
		1	rs6605081	2.36E-10	6.10E-05	Yes
		1	rs3094315	1.12E-09	0.000221032	Yes
		1	rs6603791	1.14E-09	0.000221032	Yes
		1	rs6603803	1.47E-09	0.000253353	Yes
	**PCA (10 PCs)**					
		2	rs309137	8.16E-08	8.92E-02	No
		12	rs10844115	1.61E-07	8.92E-02	No
		7	rs17139491	2.07E-07	8.92E-02	No
		1	rs2765021	2.70E-07	8.92E-02	Yes
		5	rs11948492	4.83E-07	8.92E-02	No
	**PCA (2 PCs)**					
**Analyasis 3**	**No PCA correction**					
		12	rs10844115	1.04E-07	1.50E-01	No
		1	rs3094315	1.93E-07	1.50E-01	Yes
		1	rs6605081	1.10E-06	4.54E-01	Yes
	**PCA (10 PCs)**					
		2	rs309137	8.13E-08	1.05E-01	No
		12	rs10844115	1.63E-07	1.05E-01	No
		7	rs17139491	2.36E-07	1.05E-01	No
	**PCA (2 PCs)**					
		12	rs10844115	6.04E-08	9.38E-02	No

**Analyasis 4**	**No PCA correction**					
		1	rs6605081	3.65E-14	5.68E-08	Yes
		1	rs3094315	6.40E-13	4.97E-07	Yes
		1	rs6685064	1.63E-12	8.44E-07	Yes
		1	rs6603791	6.84E-12	2.66E-06	Yes
		1	rs6603803	9.23E-12	2.87E-06	Yes
		1	rs3813199	3.42E-11	8.87E-06	Yes
		1	rs7511905	3.31E-08	0.00733734	Yes
		1	rs13303344	4.68E-08	0.00908493	Yes
		1	rs9442372	5.40E-08	0.00932276	Yes
		12	rs10844115	1.04E-07	0.0161954	No
		1	rs2765021	2.34E-07	0.0330396	Yes
	**PCA (10 PCs)**					
		1	rs6605081	2.40E-13	3.73E-07	Yes
		1	rs6685064	1.79E-12	1.39E-06	Yes
		1	rs3094315	4.38E-12	2.27E-06	Yes
		1	rs6603791	6.16E-11	2.39E-05	Yes
		1	rs3813199	1.44E-10	4.48E-05	Yes
		1	rs6603803	1.80E-10	4.67E-05	Yes
		2	rs309137	7.86E-08	0.0174528	No
		1	rs13303344	1.62E-07	0.0263707	Yes
		12	rs10844115	1.67E-07	0.0263707	No
		1	rs7511905	1.70E-07	0.0263707	Yes
		1	rs9442372	2.37E-07	0.0321543	Yes
		7	rs17139491	2.48E-07	0.0321543	No
	**PCA (2 PCs)**					
		1	rs6605081	1.81E-13	2.81E-07	Yes
		1	rs6685064	8.08E-13	6.04E-07	Yes
		1	rs3094315	1.17E-12	6.04E-07	Yes
		1	rs3813199	3.87E-11	1.51E-05	Yes
		1	rs6603791	5.73E-11	1.78E-05	Yes
		1	rs6603803	7.28E-11	1.89E-05	Yes
		12	rs10844115	6.06E-08	0.0124488	No
		1	rs7511905	6.41E-08	0.0124488	Yes
		1	rs13303344	1.18E-07	0.0203814	Yes
		1	rs9442372	1.35E-07	0.0210163	Yes
**Analyasis 5**	**No PCA correction**					
		12	rs10844115	1.04E-07	1.54E-01	No
		1	rs13303344	1.98E-07	1.54E-01	Yes
		1	rs2765021	4.04E-07	2.09E-01	Yes
	**PCA (10 PCs)**					
	**PCA (2 PCs)**					

**Analaysis 6**	**No PCA correction**					
		1	rs2765021	6.26E-09	9.73E-03	Yes
		1	rs13303344	3.05E-08	2.37E-02	Yes
		1	rs6685064	1.69E-07	8.73E-02	Yes
	**PCA (10 PCs)**					
		2	rs17031003	1.48E-60	2.30E-54	No
		7	rs7799581	7.81E-53	6.07E-47	No
		3	rs17019970	2.22E-14	1.15E-08	No
		1	rs2765021	1.41E-08	0.00547939	Yes
		1	rs13303344	1.48E-07	0.0458773	Yes
	**PCA (2 PCs)**					
		7	rs7799581	8.80E-12	1.37E-05	No
		2	rs17031003	1.32E-09	0.00102926	No
		1	rs2765021	2.41E-08	0.0124696	Yes

Table S3.2. Results of the six case-control association analyses. In each analysis, the data were calculated before and after adjusting for PCA using ten or two components. Only results with *p*<0.05 after correcting for multiple tests are shown. The last column indicates whether the causal markers found are the ones originally simulated.

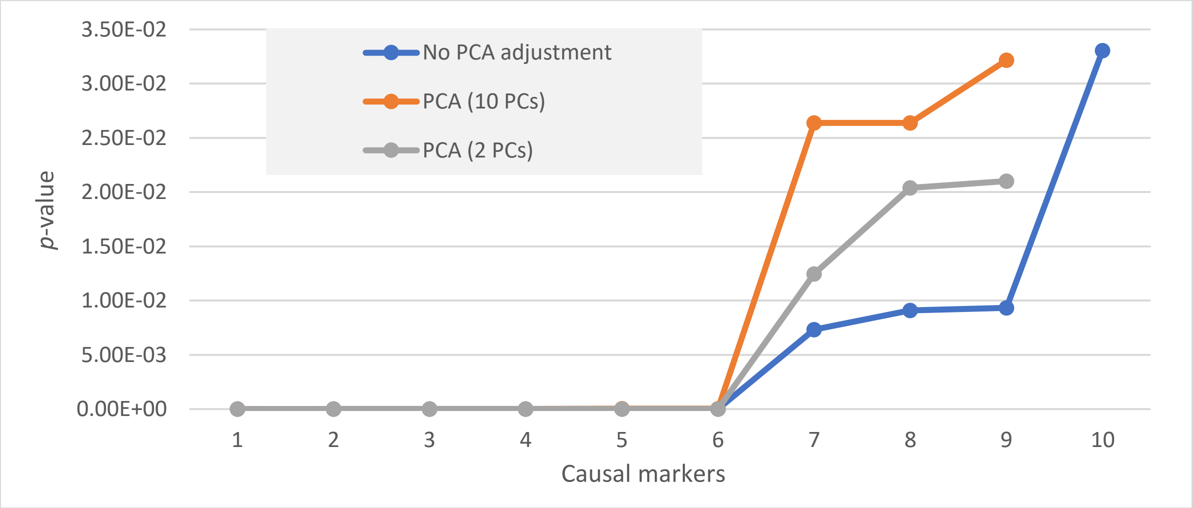

Figure S3.1. Comparing the *p*-values of casual markers identified before and after PCA adjustment.

References

1 Isshiki, M. *et al.* Admixture and natural selection shaped genomes of an Austronesian-speaking population in the Solomon Islands. *Sci. Rep.* **10**, 6872, doi:10.1038/s41598-020-62866-3 (2020).

2 Hudjashov, G. *et al.* Investigating the origins of eastern Polynesians using genome-wide data from the Leeward Society Isles. *Sci. Rep.* **8**, 1823, doi:10.1038/s41598-018-20026-8 (2018).

3 Pugach, I. *et al.* The Gateway from Near into Remote Oceania: New Insights from Genome-Wide Data. *Mol. Biol. Evol.* **35**, 871-886, doi:10.1093/molbev/msx333 (2018).

4 McEvoy, B. P. *et al.* European and Polynesian admixture in the Norfolk Island population. *Heredity* **105**, 229-234, doi:10.1038/hdy.2009.175 (2010).

5 Need, A. C., Kasperaviciute, D., Cirulli, E. T. & Goldstein, D. B. A genome-wide genetic signature of Jewish ancestry perfectly separates individuals with and without full Jewish ancestry in a large random sample of European Americans. *Genome Biol.* **10**, R7, doi:10.1186/gb-2009-10-1-r7 (2009).

6 O'Connor, T. D. *et al.* Rare variation facilitates inferences of fine-scale population structure in humans. *Mol. Biol. Evol.* **32**, 653-660, doi:10.1093/molbev/msu326 (2015).

7 Chiang, C. W. K. *et al.* Rapid assessment of genetic ancestry in populations of unknown origin by genome-wide genotyping of pooled samples. *PLoS Genet.* **6**, e1000866, doi:10.1371/journal.pgen.1000866 (2010).

8 Rahman, A., Hallgrímsdóttir, I., Eisen, M. & Pachter, L. Association mapping from sequencing reads using k-mers. *eLife* **7**, e32920, doi:10.7554/eLife.32920 (2018).

9 Genovese, G. *et al.* A risk allele for focal segmental glomerulosclerosis in African Americans is located within a region containing APOL1 and MYH9. *Kidney Int.* **78**, 698-704, doi:10.1038/ki.2010.251 (2010).

10 Luca, D. *et al.* On the use of general control samples for genome-wide association studies: Genetic matching highlights causal variants. *Am. J. Hum. Genet.* **82**, 453-463, doi:10.1016/j.ajhg.2007.11.003 (2008).

11 Willis, J. *et al.* Genome-wide analysis of the role of copy-number variation in pancreatic cancer risk. *Front. Genet.* **5**, doi:10.3389/fgene.2014.00029 (2014).

12 Mobuchon, L. *et al.* A GWAS in uveal melanoma identifies risk polymorphisms in the CLPTM1L locus. *npj Genomic Medicine* **2**, 5, doi:10.1038/s41525-017-0008-5 (2017).

13 Chang, C. C. *et al.* Second-generation PLINK: rising to the challenge of larger and richer datasets. *GigaScience* **4**, doi:10.1186/s13742-015-0047-8 (2015).

14 Benjamini, Y. & Yekutieli, D. The control of the false discovery rate in multiple testing under dependency. *The Annals of Statistics* **29**, 1165-1188, 1124 (2001).
